# Immune counter-evolution: immortalized B cell clones can undergo ex vivo directed evolution to counteract viral escape

**DOI:** 10.3389/fimmu.2025.1648717

**Published:** 2025-08-18

**Authors:** Casper Marsman, Jurgen Heinen, Vanessa Clerico Mosina, Kelly Maijoor, Arjen Q. Bakker, Michael Koslowski, Alessandra Villa, Stefano Gullà

**Affiliations:** Kling Biotherapeutics, Amsterdam, Netherlands

**Keywords:** human B cell, antibody discovery, affinity maturation, directed evolution, biparatopic antibodies

## Abstract

**Introduction:**

Amid the persistent threat of future pandemics, the continuous evolution of SARS-CoV-2 exposed critical challenges for vaccine efficacy and therapeutic interventions, highlighting the need for rapid and adaptable approaches to respond to immune escape variants.

**Methods:**

Here, we report the use of immortalized B cell libraries from human peripheral blood mononuclear cells (PBMCs) and tonsil tissues to uncover B cell clones exhibiting cross-reactive neutralization against various SARS-CoV-2 variants and perform directed evolution of immortalized B cell clones to produce antibodies with improved binding and neutralization against emerging SARS-CoV-2 variants.

**Results:**

Immortalization of PBMC and tonsil-derived human B cells was achieved through transduction with retroviral vectors encoding apoptosis inhibitors, yielding transduction efficiencies of 67.5% for PBMCs and 50.2% for tonsil-derived cells. Analysis revealed that immortalized B cell libraries produced with this method retain diverse immunoglobulin isotype representations. Through high-throughput functional screening of approximately 40,000 B cells per library, we identified 12 unique clones with neutralization activity for SARS-CoV-2, leading to selection of monoclonal antibodies with robust neutralization activity against Delta and BA.5 variants. We applied our directed evolution approach to libraries generated by ex vivo AID-induced somatic hypermutation (SHM) of immortalized B cell clones to enhance the affinity and cross-reactivity, resulting in improved binding and neutralization potency to escape variants such as EG.5.1 and JN.1. Furthermore, we engineered a bi-paratopic antibody combining KBA2401, a broadly neutralizing antibody binding to highly conserved epitope on Spike-RBD, and KBA2402, a broadly binding non-neutralizing antibody, resulting in enhanced potency against SARS-CoV-2 variant JN.1 and KP.3.

**Discussion:**

Our findings illustrate the use of immortalized B cell libraries for development of therapeutics that adapt to viral evolution and highlight the application of ex vivo directed evolution in refining antibody responses against emerging immune escape SARS-CoV-2 variants. The approach here described offers a promising pathway for rapid therapeutic development in the face of evolving viral threats.

## Introduction

B cells play a crucial role in recognizing and neutralizing pathogens via the adaptive immune response providing for long-term protective memory against infections. B cell receptor (BCR) and antibody diversity are initially generated by the combination of V(Variable), D(diversity), and J(Join) germline genes during lymphopoiesis in the bone marrow, followed by somatic hypermutation (SHM) in the germinal centers of secondary lymphoid tissues, which introduces mutations for improved antigen recognition (1, 2). This process is highly regulated to allow for the generation of antibody binders specific to *non-self* antigens. Memory B cells from patients exposed to an infection are therefore a valuable source of binding and neutralizing antibodies with wide specificities and offer an opportunity to mine for immune-functional epitopes relevant for controlling and neutralizing viral infectivity (3, 4).

Combining the diversity generated from recombination of germ line elements with antigen driven selection during SHM produces a staggering array of reactivity that serves to protect the organism from initial infection but also to adapt to viral immune escape. An example of the evolutionary chess game at play between viral evolution and immune counter-evolution is the emergence of SARS-CoV-2 in 2019 (3). The fast pace of appearance of viral escape variants has led to a decrease in the effectiveness of vaccine and antibody-based therapeutics for the prevention and treatment of COVID-19 infections (5, 6). B cells from exposed individuals respond to escape variants by evolving new reactivity leading to several monoclonal antibodies that have been isolated from individuals who have been exposed to SARS-CoV-2 infection (3). Human-derived neutralizing antibodies have helped to deepen our understanding of the adaptive immune response and identify epitopes relevant for development therapies to treat and prevent infections (4, 7). The primary target of SARS-CoV-2-neutralizing antibodies is the homotrimeric spike (S) protein, which enables the virus to enter host cells and contains various domains, such as the receptor-binding domain (RBD), the N-terminal domain (NTD), and the S2-stem domain (8, 9).

Despite the central importance of B cells in identifying treatments and vaccine epitopes, *ex vivo* manipulation of their adaptive capacity remains hampered due to the limited proliferative lifespan of primary human and animal B cells (10). Our group has built a technology platform based on the B cell transduction with a vector to express Bcl6 and Bcl-xL, which through the highly efficient isolation and immortalization of recovered patients-derived B cells, our approach captures the entire B cell diversity and all Ig-isotypes from all tissues (7). As opposed to the labor-intensive and costly approach of ex vivo BCR sequencing and subsequent recombinant antibody production and validation, immortalized B cell libraries secrete antibodies into the culture supernatant while retaining indefinite expansion capabilities, allowing for immediate functional screening of antibodies and rapid identification of unique virus-specific B cell clones (Kling-SELECT Technology) (7, 10, 11). Additionally, immortalized B cells retain the ability to undergo somatic-hypermutation, allowing for *in vitro* directed evolution of B cell clones improving several properties such as specificity towards multiple SARS-CoV-2 variants, affinity and neutralization potency (Kling-EVOLVE Technology) (11).

Here we describe the application of B cell immortalization to identify and evolve antibodies with broad reactivity against SARS-CoV-2 RBD variants. Starting from human PBMCs and tonsil tissue, we select B cells with reactivity to Wild-type and Delta variants. We identified a panel of antibodies with diverse reactivity profile against SARS-CoV-2variants going from broadly cross-reactive neutralizers to binders with specific reactivity against early strains. Further, we performed Kling-EVOLVE protocol on one clone to increase pan-variant neutralization potency and on a second clone to restore binding to recent variants. We identified the epitope of the broadly neutralizing antibody showing that it corresponds to well conserved amino acid residues of the RBD. Finally, we produced a bi-paratopic antibody by combining the variable regions of the antibodies resulting from our directed evolution approach and show that the resulting bi-paratopic antibody has stronger affinity and neutralizing properties as compared to clinical benchmark.

## Materials and methods

### Donor characteristics, B cell isolation, culture and immortalization

A single peripheral blood mononuclear cell (PBMC) sample and a single tonsil tissue sample were purchased at the commercial vendor Discovery Life Sciences, All procedures were approved by the Institutional Review Board (IRB), with standard consent forms included as an integral part of the approved study documentation. The PBMC sample is derived from a male with a self-reported SARS-CoV-2 Wuhan infection and a vaccination in 2021, serum tested positive for anti-N IgG. Tonsil donor is female with a self-reported SARS-CoV-2 Delta infection, vaccination status unknown, serum tested positive for anti-N IgG.

Fresh tonsil was dissociated with GentleMACS dissociation program additives DNAse and Collagenase per manufacturer’s instructions. Fresh Human tonsil were mechanically and enzymatically dissociated using the gentleMACS Dissociator (Miltenyi Biotec) per manufacturer’s instructions. In short, up to 4000 mg of pre-cut tonsil tissue was processed per tube in presence of pre-warmed 0.5 mg/mL Collagenase D (Merck, C4-28-100mg) and 0.1 mg/mL DNase I (Stemcell Technologies, 100-0762). Following enzymatic digestion and dissociation (Program C), cells were filtered through 70 uM Easystrain filter (Greiner, 542070), washed with medium, and counted on the CASY cell counter (OMNI Life Science).

B cells were isolated from patient peripheral blood mononuclear cells (PBMCs) or dissociated tonsil via FACS sort or EasySep Human B cell isolation kit (Stemcell tech, 17954). B cells were cultured in RPMI1640 (Gibco, 21875-034) supplemented with 8% fetal calf serum, Penicillin/Streptomycin, GlutaMAX (Thermo Fisher, 3505006), MEM Non-essential AAs (Westburg Life sciences, CA NEAA-B), Sodium pyruvate (Westburg Life sciences, CA NPY-B), Normocin (Invivogen, ant-nr-2) and Insulin-Transferrin-Selenium (Thermo Fisher, 41400045).

B cells were immortalized as previously described (11). In short, B cells were activated on hCD40L-expressing L-cells with IL-21 (50ng/ml) for 36 hours before transduction with retrovirus carrying a bicistronic construct coding for Bcl6 and Bcl-xL with a GFP fluorescence marker. After transduction B cells were cultured for 3–5 days before immortalization efficiency readout of GFP+ cells. Immortalized cells were seeded in small pools of 25 cells in 384w TCT culture plates (Spectraplate, Revvity) and cultured for 3–4 weeks to generate supernatant for screening.

B cell cultures were routinely tested for presence of mycoplasma using the MycoStrip^®^ - Mycoplasma Detection Kit (Invivogen, #rep-mys-10).

### Ig-Isotype specific staining of PBMC and Tonsil derived B cell libraries

Transduced or freshly isolated cells were washed 2-times with PBS/0.1%BSA prior to 30 minute incubation with BV421 AffiniPure Goat anti-hu IgG (Jackson ImmunoResearch) diluted 1:500, Goat F(ab’)2 Anti-Human IgA-PE (Southern Biotech) diluted 1:20.000, and Goat F(ab’)2 Anti-Human IgM-AF647 (Southern Biotech) diluted 1:5000. Afterwards samples were washed 2-times with PBS/0.1%BSA before measuring on a LSR Fortessa X-20 flow cytometer (BD).

### SARS-CoV-2 structural protein plasmids

Expression vectors were designed and cloned in a similar way as previously described (12).

SARS-CoV-2 expression constructs were designed and synthesized by GenScript (Piscataway, NJ, USA) and cloned into the pcDNA3.1 mammalian expression vector (Invitrogen) using standard molecular biology techniques. Each gene or gene combination was driven by a cytomegalovirus (CMV) or EF-1α promoter as specified, and cloned into the vector using the indicated restriction sites.

The nucleocapsid construct (pcDNA3.1-N) encoded the N gene with the R203M mutation and was cloned as a 1309 bp fragment between the NheI and PmeI sites under control of the CMV promoter. The membrane and envelope proteins were expressed from a bicistronic construct (pcDNA3.1-M-IRES-E), which included an internal ribosomal entry site (IRES) to allow co-expression. The M gene was inserted as a 2232 bp fragment (NheI–PmeI) with a downstream Woodchuck Hepatitis Virus Posttranscriptional Regulatory Element (WPRE) to enhance expression, while the E gene was cloned separately into the same vector backbone as a 2249 bp NheI–PmeI fragment, also downstream of a CMV promoter and WPRE.

The spike protein construct (pcDNA3.1-S) encoded the S gene containing the N501Y and D614G mutations, cloned as a 5140 bp fragment between MluI and XhoI sites under the control of the EF-1α promoter and without a WPRE element.

Finally, a bicistronic construct (pcDNA3.1-ORF3a-IRES-ORF7a) was generated to co-express ORF3a and ORF7a using an IRES element. ORF3a was inserted as a 1862 bp fragment using the NheI and XhoI sites, while ORF7a was cloned as a 1881 bp fragment between the NheI and PmeI sites. All constructs were sequence-verified and propagated in *E. coli* DH5α prior to transfection in mammalian cells.

### Spike-expressing HEK293T cell generation

HEK293T/17 cells were seeded at 3.0 × 10^6^ cells per 10 cm dish (Corning, 430167) in 10 mL DMEM (Gibco) supplemented with 8% FBS (Gibco, Cat# 10270-106) supplemented with Penicillin/Streptomycin (Roche, Cat# 11074440001) and incubated overnight at 37°C, 5% CO_2_. Cells at 60-70% confluency were transfected using X-tremeGENE 9 DNA Transfection Reagent (Roche, 6365809001). HEK293T cells were transfected with Spike and other surface exposed structural (envelope) proteins including M, E, N, ORF3a and ORF7a. For transfection, plasmids pcDNA3.1-N_R203M (6.6), pcDNA3.1-M-IRES-E (3.3), pcDNA3.1-EF1_Spike WPRE (0.25) and pcDNA3.1-ORF3a-IRES-ORF7a-WPR (0.125) at indicated mass ratios for a total of 10 µg of DNA were diluted in 1 mL Opti-mem (1 µg plasmid DNA/100 µl medium (12)). The plasmids were mixed with 30 µL X-tremeGENE 9 in 1 mL OptiMEM, incubated for 15 minutes, and added dropwise to cells. Cells were incubated overnight at 37°C, 5% CO_2_. The next day, the transfection medium was replaced with fresh DMEM + 8% FCS. After 48–72 hours, cells were detached using Accutase (BioLegend, 423201), resuspended, and centrifuged at 300 × g for 3 minutes. The cell pellets were resuspended in FACS buffer. The M-E-N-S-ORF3a-7a transfected cells (40 mL) were pooled, and cells were counted using the CASY counter.

### Live SARS-CoV-2 neutralization assays

The antiviral activity of antibodies and bi-paratopic constructs was evaluated *in vitro* using a cytopathic effect-based live SARS-CoV-2 neutralization assay in VeroE6 cells overexpressing transmembrane serine protease 2 (TMPRSS2).

#### Cell line and culture conditions

Vero E6/TMPRSS2 cells were employed for live SARS-CoV-2 neutralization assays due to their enhanced susceptibility to infection. TMPRSS2 expression enables efficient spike protein cleavage and robust viral replication kinetics, providing a physiologically relevant model of human airway infection. This system is well-established for neutralization studies, demonstrating high permissiveness, clear cytopathic effects, and strong correlation with other hACE2-expressing models. Its use aligns with standardized protocols adopted by other virology laboratories [e.g (13–15)]. Vero E6/TMPRSS2 cells were cultured as a monolayer at 37°C in a humidified incubator with 5% CO_2_. Cells were maintained in DMEM supplemented with 10% Fetal Bovine Serum, 1% pyruvate, 1% antibiotic cocktail (penicillin, streptomycin, and geneticin). Cells were passaged with trypsin-EDTA and seeded in 96-well plates at 20,000 cells/well in 200 µL complete media approximately 18 hours prior to infection.

#### Virus preparation

All mentioned SARS-CoV-2 variants were amplified in VeroE6/TMPRSS2 cells. Each variant was titrated against a positive and negative neutralization controls to ensure equivalent infection efficiency across variants and allowing for sensitive testing of antibodies.

#### Neutralization procedure

Each antibody was serially diluted as follow: 11-point, 3-fold dilutions or 7-point, 4-fold dilutions. Diluted antibodies were pre-incubated with the virus at 37°C for 30 minutes to allow binding and potential neutralization. Neutralizing controls (Bebtelovimab/Pemivibart) or negative controls (anti-HA antibodies) were taken along. The antibody-virus mixture was then added to pre-plated cells and incubated for 2 hours at 37°C. After incubation, the mixture was removed and replaced with fresh complete growth medium.

#### Readout

Cell viability was assessed using the MTS/PMS assay. At 48 hours post-infection, 20 µL of MTS/PMS reagent (20:1 ratio) was added directly to each well of the 96-well plate containing 200 µL of culture medium. Plates were incubated at 37°C for 4 hours, and absorbance was measured at 490 nm using a microplate reader.

#### Data analysis

Raw absorbance values were collected using an ELISA plate reader and analyzed using standard curve fitting models to interpolate neutralization thresholds.

### SARS-CoV-2 pseudovirus neutralization assay

HEK293T/hACE2 cells (VectorBuilder, Cat# CL0014) were cultured in DMEM (Gibco) supplemented with 8% FBS (Gibco, Cat# 10270-106) and Penicillin/Streptomycin (Roche, Cat# 11074440001). On the first day, ~20,000 cells/well were seeded in 50 µL DMEM into White, TCT, 96-well plates (Revvity, CulturPlate-96, Cat# 6055680). Plates were incubated overnight at 37°C. The following day, antibodies were prepared at 300 nM (3× final concentration) in Opti-MEM™ I Reduced Serum Medium (Thermo Fisher, Cat# 31985070). An 8-point 1:5 serial dilution was performed. 25 µL of each dilution was transferred to 96-well FACS plates. Replication-deficient Moloney Murine Leukemia Virus (MLV) pseudotyped with the SARS-CoV-2 spike protein (eEnzyme) was used as the viral input. 50 µL of virus was added per well and incubated 1 h at 37°C. After removing media from the cell plates, 75 µL of the virus-antibody mix was added per well. Plates were centrifuged at 700 rpm for 15 min (RT) and incubated at 37°C. On Day 3, 25 µL of DMEM was added to each well (final volume: 100 µL). On the last day (day 4), luciferase activity was measured using the Firefly Luciferase Assay Kit (eEnzyme, Cat# CA-L165-10). The working solution was prepared according to manufacturer’s instructions. 100 µL of reagent was added per well, incubated for 3–5 min on a shaker (600 rpm), and luminescence was measured using an Envision fluorescence plate reader.

### Protein expression and purification

#### SARS-CoV-2 spike proteins

Recombinant SARS-CoV-2 spike proteins were expressed in HEK293 cells. Constructs included a C-terminal His-tag for affinity purification and an Avi-tag for enzymatic biotinylation. Following transient transfection, supernatants were harvested, and the spike proteins were purified using affinity chromatography. Biotinylation was subsequently performed enzymatically using the BirA ligase to target the Avi-tag.

#### Monoclonal antibodies

Monoclonal antibodies were expressed in Chinese Hamster Ovary (CHO) cells via transient transfection. Cell culture supernatants were collected post-expression and antibodies were purified by Protein A affinity chromatography with acidic elution (acetate pH 3.4). Final purity and integrity of KBA2401, KBA2402, KBA2403, KBA2404 and KBA2401_EVO variants were confirmed by SDS-PAGE and size-exclusion chromatography. For KBA2402_EVO variants only 1 ml of CHO supernatant was produced, quality control (QC) included concentration measurement: IgG concentration in CHO cell culture supernatant was measured using the Gator Bio GatorPlus instrument with Protein A (ProA) biosensor tips. Samples were diluted in Gator Q-buffer and loaded into GatorMax 96-well plates according to the manufacturer’s protocol. Binding responses were recorded in real-time, and IgG concentrations were quantified using a standard curve generated from known IgG standard in the same buffer and plate.

#### Bi-paratopic antibodies

Bispecific and bi-paratopic antibodies were also produced in CHO cells. After expression, antibodies were isolated from the culture medium using Protein A affinity purification. Final purity and integrity of all productions were confirmed by SDS-PAGE and size-exclusion chromatography.

All monoclonal and bi-paratopic sequences (NT and AA) and QC data can be found in **Supplementary Table 1** and **Supplementary Figure S5**.

### SARS-CoV-2 Spike specific staining of immortalized B cells

Spike-streptavidin complexes were generated as previously described with minor modifications. (https://www.sciencedirect.com/science/article/pii/S2666166722007821). In short, mono-biotinylated Spike proteins (Acro Biosystem, or custom order at BioIntron) were added at a 4: 1 molar ratio to fluorescently labeled streptavidin (PE or PE-Cy7 Thermo Fisher) and incubated 30 min at RT, afterwards 2 uM d-biotin (Avidity) was added to block any free sites on the streptavidin. Spike-streptavidin tetramers were added directly to immortalized B cells and incubated for 30 min on melting ice. Afterwards cells were washed 2-times with 10x volume of culture medium. Cells were kept cold until sorting using a FACS ARIAIII cell sorter set to cold (4 °C) setting, see below.

### FACS ARIA based cell sorting

#### Single cell sorting of minipools

Minipool cultures were harvested, washed with 1 ml complete medium (300 × g, 5 min), and resuspended in 300 μl complete medium. The BD FACSAria III was calibrated according to the manufacturer’s instructions, including CS&T setup and configuration for 384-well plate sorting. A 100-μm nozzle was used, with the sort rate kept below 5,000 events/sec to minimize cell stress. Single-cell mode was used to ensure clonal outgrowth. GFP-positive immortalized B cells were sorted into 384-well plates pre-coated with L-cells in 25 uL Complete medium. For each minibulk, half of a 384-well plate was sorted, resulting in 20 plates for 40 bulks. After sorting, 25 μl of complete B cell medium containing 100 ng/ml rmIL-21Fc was added to each well, resulting in a final volume of 50 μl per well with 50 ng/ml IL-21. Plates were cultured for 3 weeks to allow for outgrowth. During expansion, cultures were fed with L-cell and Il-21 each week.

#### Antigen-based single-cell sorting for affinity maturation

Spike-Streptavadin complex stained B cells were resuspended in 0.3 ml medium without FCS, kept on ice. The BD FACSAria III was prepared as described above with minor adjustments: The sort mode was set on purity mode and the machine was calibrated for single-cell deposition into 96-well PCR plates (Bio-Rad, MLL9601).

PCR plates were preloaded with 9.2 μl ddH_2_O containing 0.5 μl each of 10 μM VH and VL family-specific RT-PCR primers (final 0.25 μM per primer).

Single Spike variant-high binding (PE/PE-Cy7+) B cells were sorted (1 cell/well) into the prepared 96-well PCR plates according to the plate layout, targeting the top 0.1% of binders for each clone. Sorted cells were immediately processed for RT-PCR and sequencing.

### HTP sequencing of heavy and light chain BCRs

B cell receptor (BCR) sequencing was conducted on up to 10,000 single B cells per sample. Single-cell BCR libraries were generated using either the Singleron sCircle BCR sequencing kit according to the manufacturer’s instructions or custom-designed in-house primers targeting immunoglobulin heavy (VH) and light (VL) chain variable regions. cDNA synthesis and amplification were performed according to standard protocols. Amplified products were barcoded and prepared for sequencing using the Oxford Nanopore Technologies (ONT) Native Barcoding Kit 24 V14 (SQK-NBD114.24). Sequencing was performed on ONT Flow Cell (R10.4.1) FLO-MIN114. Base calling and demultiplexing were carried out using ONT’s MinKNOW software. BCR Heavy and Light sequence analysis was performed using the IgX Platform (ENPICOM).

## Results

### Unbiased screening of immortalized B cell libraries derived from human PBMCs and tonsils produce B cell clones with cross reactive neutralization to SARS-CoV-2

**Figure 1:**
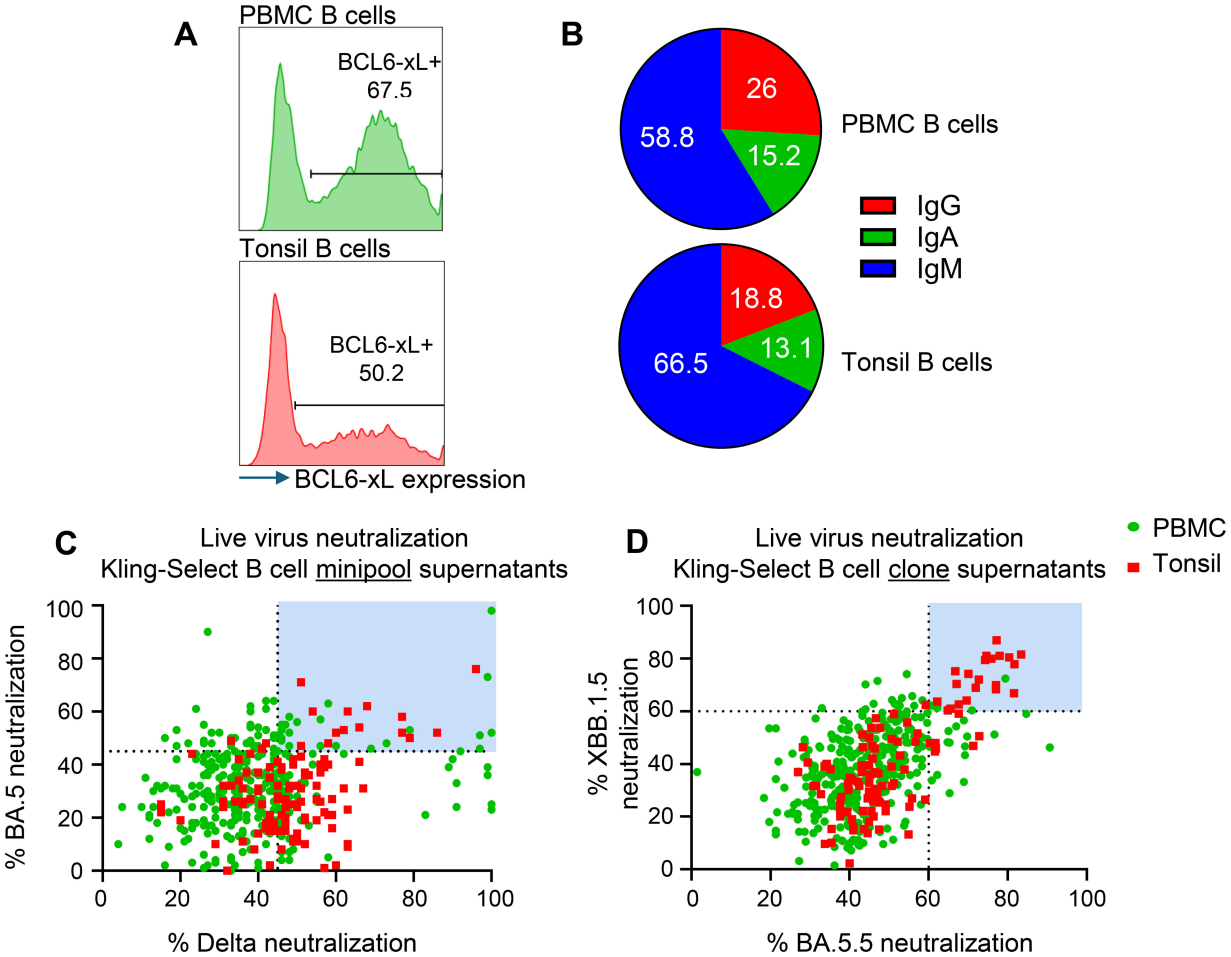
Generation and functional screening of immortalized B cell libraries derived from human PBMCs and tonsils for SARS-CoV-2 neutralizing activity. **(A)** Immortalization efficiency in PBMC derived (green histogram) and tonsil derived B cells (red histogram). **(B)** Pie charts depicting the Ig isotype distribution of IgM- (Blue), IgG- (red) and IgA- (green) expressing B cells in the PBMC derived and Tonsil derived immortalized samples. **(C)** Live virus neutralization of Delta and BA.5 variants by antibody-containing supernatants from immortalized minipools. Shown are individual wells of PBMC derived (green) and Tonsil derived (red) minipools. Light blue panels indicate selected MPs carried forward for monoclonal screening. **(D)** Live virus neutralization of XBB.1.5 and BA.5 variants by antibody-containing supernatants from immortalized clones. Shown are individual wells of PBMC derived (green) and Tonsil derived (red) clones. Blue panel shows selected minipools for sequencing.

We produced immortalized B cell libraries from fresh human PBMCs and frozen human tonsil tissue by transduction with retrovirus carrying apoptosis inhibitor genes BCL-6 and BCL-xl genes and GFP as a marker of positive transduction. We observed immortalization efficiency by measuring the percentage GFP+ cells 4 days after transduction and found that 67.5% and 50.2% transduction efficiency for PBMC and tonsil derived libraries, respectively (**Figure 1A**). The different immortalization efficiency might be due to the handling of the two samples: the PBMCs were used fresh, while the tonsil sample was obtained from frozen tissue. Using flow cytometry we measured the relative abundance of IgG, IgM and IgA positive B cell receptors of each of the libraries. All three Ig isotypes were present in the immortalized libraries, albeit at different relative abundances (**Figure 1B**). For both PBMC and tonsils, we noted that the majority of B cells to be of the IgM subtype, with IgG being represented by 26% and 19% of clones in the PBMC library and tonsil library, respectively. The higher IgM representation in the tonsil sample may reflect the nature of lymphoid organ as reservoir of naïve B cells. Moreover, IgA positive B cells were detected on 15% in the PBMC library and 13% in the tonsil library (**Figure 1B**), showing representative capture of the Ig repertoire in both B cell sources.

We produced ‘minipools’ (MPs) from each library by sorting 25 cells per well in a total of ten, 384-well plates. The resulting MPs were cultured for 3 weeks to allow expansion of B cell clone and accumulation of secreted antibodies in the culture medium. We identified MPs with reactivity to SARS-CoV-2 proteins by measuring binding of the conditioned medium to Spike and envelope proteins expressed by HEK293T cells (**Supplementary Figure S1A**). Reactive MPs were identified based on relative binding compared to positive control antibody Bebtelovimab. We found 474 MPs from the PBMC library and 123 from the tonsil library with positive reactivity (**Supplementary Figure S1A**). Selected MPs were tested for live virus neutralization against Delta and BA.5 (**Figure 1C**). The MPs that showed >45% neutralization of both BA.5 and Delta variants were subcloned by single-cell FACS sort into 384-well plates and the monoclonal populations were cultured for 3 weeks to produce supernatant containing monoclonal secreted antibody. We selected 440 monoclonal supernatants, that showed binding to Spike and envelope proteins expressed by HEK293T cells (**Supplementary Figure S1B**), to test in a live virus neutralization assay against two SARS-CoV-2 variants: XBB.1.5 and BA.5.5 (**Figure 1D**). The majority of tested monoclonal supernatants showed greater than 50% neutralization against both variants, with 26/440 showing greater than 60% neutralization against both variants (highlighted in the blue box, **Figure 1D**), 24/440 having neutralization bias for BA.5.5 and 26/440 having neutralization bias for XBB.1.5 (**Figure 1D**). Of note, tonsil derived B cells showed lower relative binding and a lower number of hits (**Supplementary Figure S1**) but increased presence on cross-neutralizing clones on both tested variants (**Figure 1D**). We sequenced the BCRs from all B cell clones with neutralization greater than 60% for both variants and identified 12 unique VH/VL pairs.

### Recombinant antibodies derived from selected immortalized B cell clones display diverse binding and neutralizing profiles against panel of SARS-CoV-2 variants

The stably immortalized B cell clones selected were found to express antibodies of IgA, IgG and IgM isotypes (**Table 1**).

**Table 1:** Overview of the immortalized B cell clones sequenced depicting information on the isotype, Heavy-chain variable gene family usage, light-chain variable gene family usage and mutations found compared to germ line.

Clone	Isotype	IGHV	H-V-gene mutations	IGHD(frame)	H-D-gene mutations	IGHJ	H-J-gene mutations	IGKV/LV	L-V mutations	IGK/LJ	L-J mutations
1B10	IgA1	5-51*01 F	4	5-24*01	0	3*01F or 4*03F	0	LV3-21*02 F	5	LJ2*01 F	1
1E7	IgA1	3-49*03 F	5	5-24*01	0	4*02 F	0	KV2-28*01 F	1	KV5*01 F	0
3D11	IgG1	3-66*01 F	12	3-10*01 F	0	6*02 F	1	KV1-9*01 F	4	KJ4*01 F	2
4H3	IgG1	3-66*01 F	5	3-16*02 F	0	3*02 F	0	KV1-5*04 F	8	KV2*01 F	0
1B4	IgM	3-74*01 F	1	4-17*01 F	0	4*02 F	0	KV4-1*01 F	0	KJ*01 F	0
2F3	IgM	4-34*03 F	1	2-2*01 F	0	6*02 F	0	LV1-40*01 F	10	LJ2*01F	1
2G11	IgM	3-30*018F or 3-30-5	10	4-23*01 F	2	4*02 F	1	LV1-44*02 F	5	LJ2*01F or LJ3*01 F	0
3D10	IgG1	4-61*12 F	8	2-2*01 F	4	3*01 F or 3*02 F	1	KV1-33*01F	5	KJ2*01 F	0
3F9	IgG1	4-34*02F	8	3-10*01F	0	4*02 F	2	LV1-51*01F	6	LJ1*01F	1
3H8	IgG4	5-51*01 F	15	4-17*01F	2	5*01F ir 5*02 F	1	LV3-25*02F	16	LJ2*01F or LJ3*01F	1
4G10	IgG1	4-39*07 F	15	5-18*01F	1	5*02F	2	LV1-51*02F	7	LJ2*01 F or LJ3*01F	1
5G2	IgG1	7-4-1*02F	6	5-24*01F	1	3*02F	0	LV3-19*01F	14	LJ2*01F	1

**Figure 2:**
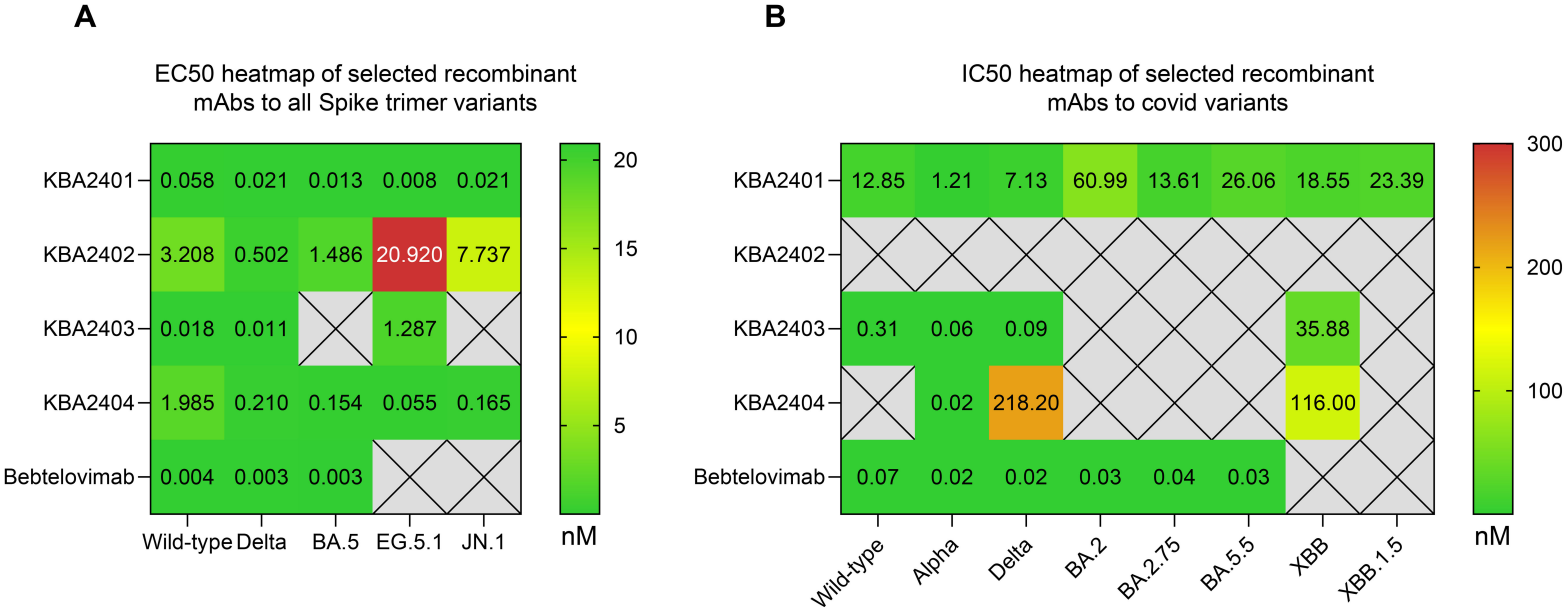
Binding and neutralization of SARS-CoV-2 spike variants by recombinant IgG1 antibodies, shown as heatmaps. **(A)** Heatmap of ELISA-derived EC_50_ values (nM) for antibodies KBA2401–KBA2404, expressed in IgG1 format, tested against spike trimers from SARS-CoV-2 variants including Wild-type, Delta, BA.5, EG.5.1, and JN.1. KBA2401 and KBA2404 bound all tested variants, while KBA2402 and KBA2403 exhibited reduced binding to more recent variants. **(B)** Heatmap of live virus neutralization IC_50_ values for selected antibodies against viruses bearing spike proteins from Wild-type, Alpha, Delta, BA.2, BA.2.75, BA.5.5, XBB, and XBB.1.5.

The variable regions were produced recombinantly in IgG1 format regardless of the original isotype and were tested for binding by ELISA to various SARS-CoV-2 spike trimer protein variants. All analyzed antibodies showed reactivity to spike proteins from the original Wild-type and Delta strains. KBA2401 and KBA2404 were able to bind with high affinity to all spike proteins tested, while KBA2402 and KBA2403 showed a more selective binding profile, with KBA2403 losing binding to BA.5 and JN.1 derived spike. KBA2402 instead showed decreasing reactivity against EG.5.1 and JN.1 derived spike trimer (**Figure 2A**).

We next tested the ability of selected recombinant antibodies to neutralize live virus carrying Wild-type, Alpha, Delta, BA.2, BA.2.75, BA.5.5, XBB and XBB.1.5 spike variants (**Figure 2B**, **Supplementary Figure S2A**). Results confirmed the ability of KBA2401 to broadly neutralize live virus infections across all tested variants and the more selective neutralization of KBA2403 of earlier variants, such as Wild-type, Alpha and Delta, and no neutralization of recent variants, such as BA.5.5. or XBB.1.5. We also noted that KBA2402 did not have neutralization activity at the tested concentrations against any of the tested variants raising the possibility that this antibody binds to a non-blocking epitope on the spike trimer. This assumption was confirmed by testing binding of KBA2402 to Spike Trimer or Spike RBD, showing that KBA2402 does not bind the RBD (**Supplementary Figure S2B**). As KBA2404 showed activity against only a few SARS-CoV-2 variants, this antibody and the KBA2403 antibody were not further investigated.

### *Ex vivo* directed evolution of immortalized B cell clones produces antibodies with improved potency and cross reactivity

**Figure 3:**
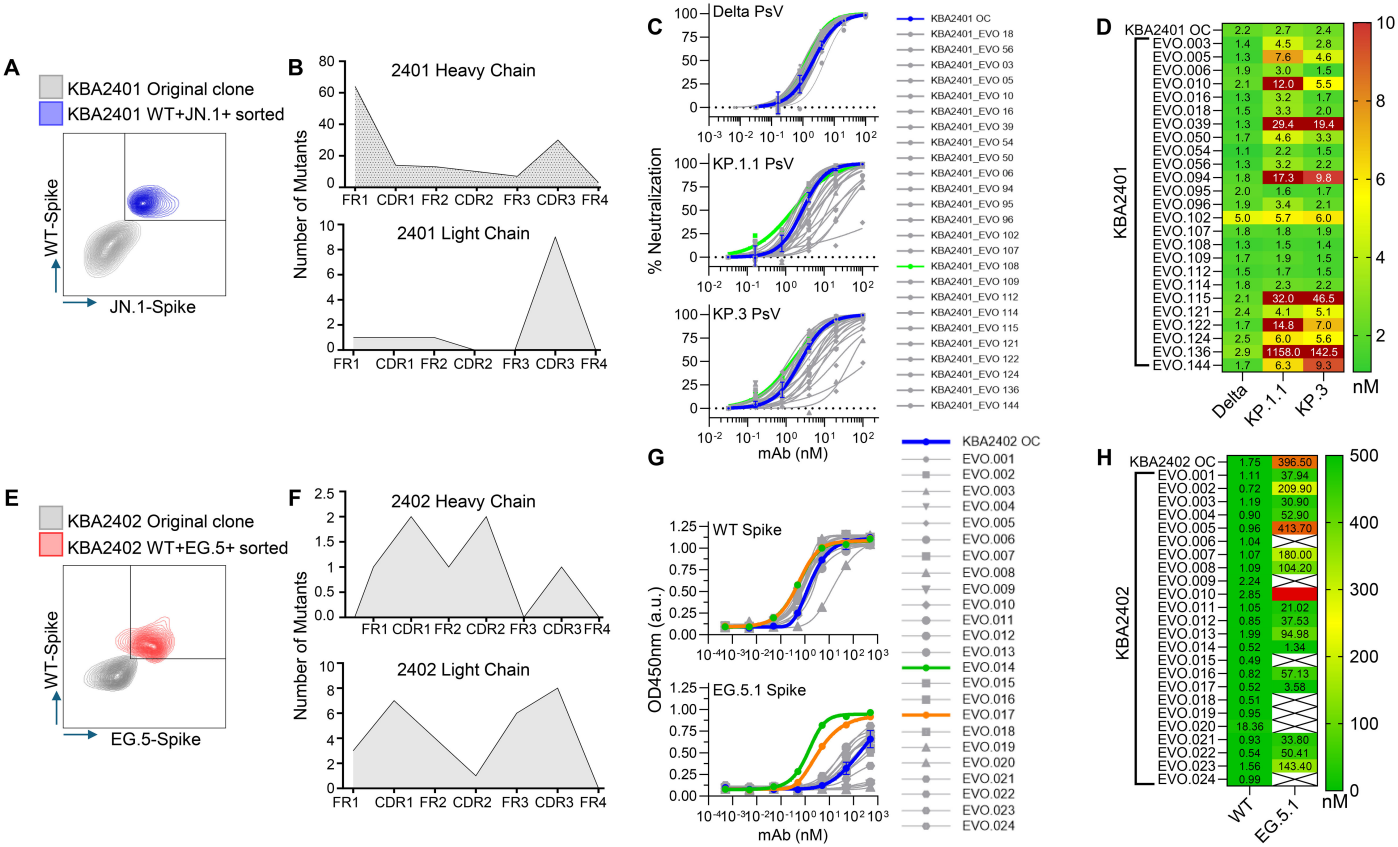
Directed *in vitro* evolution of human B cell clones using the Kling-EVOLVE protocol to enhance antibody potency and variant reactivity.**(A)** Schematic of the Kling-EVOLVE approach applied to clone 1B10 (KBA2401), including three rounds of culture under AID-inducing conditions and antigen-specific sorting to enrich B cells with increased binding to Wild-type and JN.1 spike trimers. The blue population was selected for BCR sequencing and SHM analysis. **(B)** Summary of somatic hypermutation (SHM) across evolved 1B10 populations. The heavy chain acquired 70 unique mutations, with hotspots in framework region 1 (FW1), while the light chain remained more conserved, with 10 unique mutations. A total of 147 recombinant antibody variants (KBA2401_EVO) were generated in IgG1 format by combining selected mutations. **(C)** Overview of the neutralization screening for KBA2401_EVO variants using pseudotyped viruses expressing Delta, KP.1.1, and KP.3 spikes. The KBA2401 original clone is depicted in blue, KBA2401_EVO108 is depicted in light green, all other EVOs are depicted in grey. **(D)** Heatmap of pseudovirus neutralization IC_50_ values for KBA2401_EVO variants compared to the parental KBA2401. Most evolved variants showed improved potency, particularly against KP.1.1 and KP.3. KBA2401_EVO108 demonstrated the greatest improvement, with an average 60% reduction in IC_50_ compared to the parental clone. **(E)** Schematic of the Kling-EVOLVE protocol applied to clone 1E7 (KBA2402), aiming to restore binding to escape variant EG.5.1. Three rounds of AID-induced culture followed by antigen-specific sorting were performed, selecting for B cells with dual reactivity to Wild-type and EG.5.1 spike proteins. The red population was selected for BCR sequencing and SHM analysis. **(F)** Overview of SHM in evolved 1E7 populations. A total of 34 unique CDR mutations were identified across heavy and light chains. These were combined to produce 24 recombinant antibody variants (KBA2402_EVO) in IgG1 format. **(G)** ELISA binding curves comparing KBA2402_EVO variants and parental KBA2402 to Wild-type and EG.5.1 spike trimers. **(H)** Heatmap of pseudovirus neutralization EC_50_ values for KBA2402_EVO variants compared to the parental KBA2402.

The stable B cell clones produced by our B cell immortalization approach retain the ability to undergo AID-induced somatic hypermutation (SHM). We can control the induction of AID by culturing the B cells under certain conditions which we refer to as the Kling-EVOLVE protocol. We explored the possibility of inducing SHM in the selected B cell clones and applying selective pressure to generate higher affinity B cell clones against their respective targets. To do so, we titrated down antigen-complex concentration to near-background staining levels and sorted only on the top 0.1 - 1% binding clones to derive new antibody variants with increased potency and cross reactivity. While a similar approach has been shown before (11) to increase affinity of B cells we selected B cell clones 1B10 and 1E7 corresponding to antibodies KBA2401 and KBA2402 respectively to test two ovel applications of directed evolution: 1) for 1B10 we aimed to increase binding and neutralization while maintaining broad cross reactivity, and 2) for 1E7 we aimed to restore binding to recent SARS-CoV-2 variants where the original clone had lost reactivity (**Figure 3**).

#### Directed evolution of 1B10 clone for increased affinity and cross-reactivity

We cultured clone 1B10 under Kling-EVOLVE protocol and used antigen specific sorting to isolate clones with increased reactivity against Wild-type and JN.1 spike trimers. We performed 3 rounds of sorting, sequentially selecting for B cells with increased binding, followed by HT-sequencing of the highest binding double positive population to identify new antibody variants (**Figure 3A**, left panel).

Heavy and light chains were found to contain mutations at different frequencies (**Figure 3B**). The heavy chain analysis yielded a total of 70 single mutants across the variable region. Hotspot mutations were present in FW1 potentially indicating a stabilizing amino acid substitution, which conferred expression advantage for the clone. The light chain by contrast was found to be better conserved with fewer total unique clones represented by 10 mutations compared to parental. Altogether we identified a total of 81 mutations in the evolved population compared to the parental. We further combined mutations to produce a total of 147 new antibody sequences based on the parental clone. All evolved variants (EVO) were recombinantly produced in IgG1 format.

We then tested the KBA2401_EVO variants in a pseudovirus assay to determine the neutralization potency (**Figure 3C**). We find that the majority of clones showed improved IC_50_ values against Delta, KP.1.1 and KP.3 pseudovirus (**Figure 3D**) while some clones lose potency particularly against KP.1.1 and KP.3 compared to Delta. KBA2401_EVO108 showed the most improvement compared with parental with an average 60% improvement in the IC50 going from an average of 2.43nM for the parental to 1.53nM for the three pseudoviruses tested (**Figure 3D**).

#### Directed evolution of 1E7 clone for increased binding to escape variant EG.5.1

We find the binding profile of clone 1E7 interesting in the context of viral escape. This clone shows strong reactivity to early SARS-CoV-2 strains such as Wild-type and Delta but loses binding to more recent Omicron strains such as EG.5.1 and JN.1. We wanted, therefore, to test whether our Kling-EVOLVE protocol might be able restore 1E7 reactivity to more recent SARS-CoV-2 variants. We applied our EVOLVE protocols to 1E7 by culturing the clone for 2–3 weeks under AID inducing conditions in an effort to generate sequence diversity via SHM, before sorting the cells with labeled spike-streptavidin tetramers. This cycle of culturing the cells for 2–3 weeks and subsequent clone sorting was repeated 3 times. During each sort, clones with increased double reactivity to Wild-type and EG.5 spike were selected in order to retain the binding to both early and later spike variants (**Figure 3E**). After 3 rounds of sorting, expansion and resorting, we performed HTP-sequencing of the highest binding population to identify new antibody variants.

The sorted population displayed mutations across CDRs and framework regions of both heavy and light chain variable domains (**Figure 3F**). We isolated a total of 34 unique mutations across the CDRs of the Heavy and Light chains, combined these and produced a total of 24 recombinant KBA2402_EVO antibodies as IgG1.

We measured binding of KBA2402_EVO mutants and KBA2402 parental antibody against recombinant spike protein from the Wild-type and EG.5.1 strains by ELISA. Several KBA2402_EVO antibodies showed improved binding to both Wild-type and EG.5.1 spike (orange and green line in **Figure 3G**). Of note, two KBA2402_EVO antibodies (EVO14, EVO17) show 100 to 400 fold improved EC50s towards EG.5.1 spike as compared to KBA2402 parental, while also showing increased binding to Wild-type spike (**Figure 3F**).

### Broadly neutralizing KBA2401 antibody binds to conserved epitope on spike RBD

**Figure 4:**
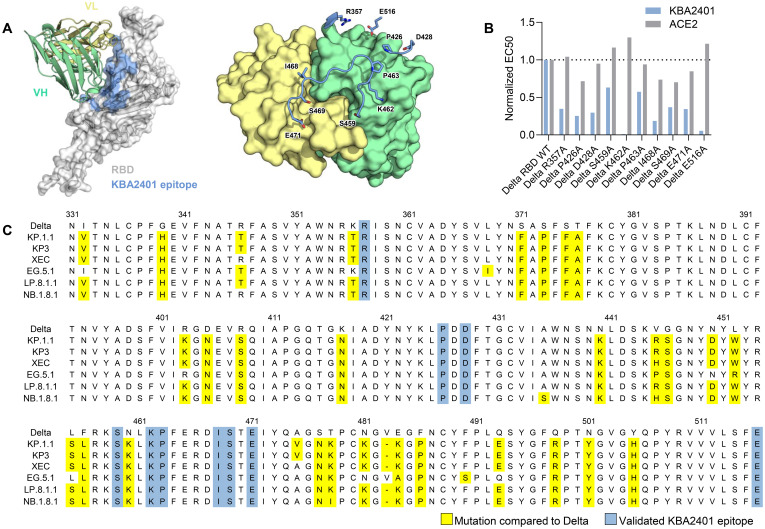
KBA 2401 epitope analysis. **(A)** 3D PyMOL rendering of VH and VL of KBA2401 with spike RBD, based on AlphaFold 2 simulation. Left panel: RBD in grey (surface representation with ribbon backbone); right panel VH in cyan and VL in light gold (ribbon representation). **(B)** Normalized ELISA data of the binding of ACE2 receptor (soluble domain) in grey, and of KBA2401 in green to Alanine-mutants RBD. **(C)** Primary structure analysis of the conserved residues among different SARS-CoV-2 virus strains (in yellow) in comparison to the amino acids relevant for KBA2401 binding (in green).

We investigated KBA2401 to determine the binding epitope. We used AlphaFold2 to predict the binding interface between the variable regions of KBA2401 and an RBD monomer (modeling score = 0.83). In order to test the validity of the predicted epitope we produced alanine substitutions of the sites most likely buried in the binding interface. We identified 10 amino acids on spike-RBD that are normally solvent exposed but are predicted to directly interact with the KBA2401 paratope (**Figure 4A**).

We recombinantly produced the single alanine-RBD mutants and tested binding of KBA2401 by ELISA. We also tested binding of spike RBD alanine mutants to human ACE2 soluble receptor protein to confirm that the RBD-alanine mutants maintained native binding (**Figure 4B**). We find that 8 of the 10 alanine substitutions on Delta-RBD inhibit KBA2401 binding by more than 50% compared binding observed with Delta WT-RBD. In particular we find that alanine substitutions at site K462 and E516 completely abrogate KBA2401 binding while maintaining ACE2 binding, confirming the AlphaFold2 binding prediction. As comparison for this analysis, we run in parallel Pemivibart antibody (**Supplementary Figure S4**), where P426, I468, and S469 play a major role in binding abrogation.

RBD spike residues involved in KBA2401 binding are conserved between ancestral and recent strains, including the latest ‘Nimbus’ variant NB.1.8.1 (**Figure 4C**), thus explaining the broad binding and neutralization activity of the antibody. Interestingly, the epitope does not appear to directly compete with ACE2 binding, rather it may neutralize viral infection allosterically by stabilizing a non-productive form of the spike RBD.

### Bi-paratopic antibody combining broadly neutralizing and broadly binding non-neutralizer shows improved potency against recent JN.1 SARS-CoV-2 escape variant

**Figure 5:**
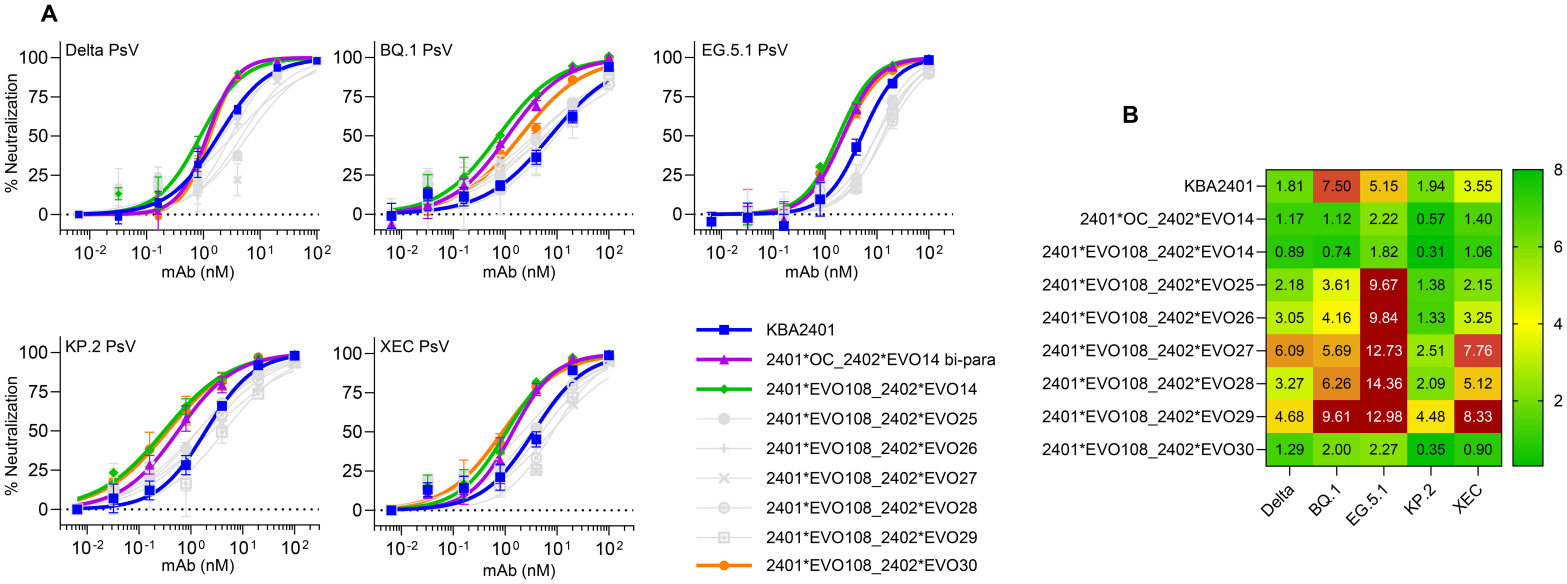
Enhanced neutralization of SARS-CoV-2 variants by bi-paratopic antibodies combining KBA2401 and KBA2402-derived EVOLVED paratopes. **(A)** Pseudovirus neutralization curves for Delta, BQ.1, EG.5, KP.2, and XEC spike variants comparing monovalent and bi-paratopic antibodies. KBA2401 is shown in blue; bi-paratopic antibody 2401*OC_2402*EVO14 in purple; 2401*EVO108–2402*EVO14 in green; and 2401*EVO108-2402*EVO30 in orange. Additional bi-paratopic constructs are shown in grey. bi-paratopic antibodies were generated by fusing a KBA2402 scFv to the light chain of KBA2401 or KBA2401_EVO108 via a glycine-serine linker, resulting in tetravalent antibodies targeting distinct spike epitopes. **(B)** Heatmap of IC_50_ values derived from pseudovirus neutralization assays.

Upon discovery and subsequent affinity optimization of the KBA2401 and KBA2402 monoclonal antibodies, and after showing that these bind distinct regions of the Spike protein (**Supplementary Figure S2B**), we reasoned that combining the two binding domains of KBA2401 and KBA2402 into a Bi-paratopic construct would allow for binding of multiple epitopes on the same antigen with the potential benefits of increasing potency and reducing risk of loss of binding due to escape variants. We tested the combination of KBA2401 with KBA2402 in bi-paratopic constructs by expressing the KBA2402 as an scFv fused to the light chain of KBA2401 via a glycine-serine linker. The resulting tetravalent antibody contained binding paratopes of both the pan-neutralizing antibody KBA2401 and the non-neutralizing, but cross-variant binding, antibody KBA2402. We tested the ability of the bi-paratopic antibody to neutralize *in vitro* Delta, BQ.1, EG.5.1, KP.2 and XEC Pseudovirus (**Figure 5**) and live virus infection of SARS-CoV-2 JN.1 (**Supplementary Figure 4**) compared to KBA2401. The neutralization assays showed that the bi-paratopic antibody had more potent cross-variant neutralizing activity compared to KBA2401.

## Discussion

The rapid evolution of viruses, in particular RNA viruses, contributes to their ability to cause recurring seasonal illnesses, as they can often evade existing immunity in the population. SARS-CoV-2, influenza (flu), rhinovirus are examples of viruses which are globally present, endemic to human populations and have shown to be difficult to eradicate due to their ability to adapt to natural immunity, vaccination or therapeutic approaches (4, 16–18). Due to a combination of rapid mutation, short generation times, large host population size and selective pressure from host immune defenses they pose an ever-changing public health threat. In addition to causing widespread seasonal infectivity, viral evolution has the potential to produce hyper-virulent variants that may have greater morbidity and mortality rates compared to the parental strain. New flu vaccines are developed each year to match the circulating strains, however seasonal vaccines are not always effective at producing sufficiently broad immune protection to avoid escape variants from spreading (4, 16). The human immune system is constantly engaged in adapting to emerging viral strains via a process of counter-evolution, whereby B cells undergo their own evolution via SHM to produce neutralizing antibodies better suited at responding to new strains (3). Here, we show how immortalized human B cells can be used *ex vivo* via a directed evolution approach to restore activity against SARS-CoV-2 viral escape variants.

Memory B cells are an exceptional source for discovery of therapeutic antibodies and their cognate epitopes. By interrogating the reactivity of antibodies produced from natural infections we can gain insights into immuno-functional epitopes that represent potential targets for both vaccines and therapeutics (3, 10). The B cell immortalization technology employed for this work relies on the transduction of Bcl-6 and BcL-xL, enabling the immortalization of human B cells with unprecedented efficiency (7). Immortalized B cells produced by this method overcome the limited *ex vivo* lifespan and proliferation of primary B cells and therefore are suitable for high-throughput screening. Our approach aims to identify B cell clones expressing functional antibodies and leverage the innate adaptive capacity of B cells to evolve via AID-induced SHM diversity and selective pressure (7). In this work, immortalization of B cell from PBMCs and from dissociated tonsil samples originating from SARS-Cov-2 exposed individuals led to (i) the identification of immortalized B cell clones producing neutralizing antibodies against SARS-Cov-2; (ii) *ex vivo* directed evolution of selected clones to improve affinity and cross reactivity to SARS-CoV-2 escape variants (iii) determination of a highly conserved epitope displaying broad neutralizing activity (iv) production of a potent bi-paratopic antibody with broad neutralization activity of ancestral and recent SARS-CoV-2 variants.

### Immortalized B cell libraries as source of immuno-functional epitopes

The immortalization technology here described captures entire B cell repertoires, mirroring the different subtypes present in the two different tissue samples. IgM clones were the predominant population in both PBMC and tonsil-derived samples, while IgG were slightly higher in the blood-circulating population. Interestingly, more SARS-CoV-2 specific B cell binders were detected in the circulating PBMCs, while the majority of neutralizing clones were identified from the tonsil sample, potentially due to somatic hypermutation and selection of highest affine clones through repeated viral exposure. Investigation on the epitope of KBA2401 antibody showed that its neutralization potency was not due to direct binding competition with ACE2 receptor, but to an epitope potentially triggering an allosteric conformational change thus hindering the binding to ACE2 and consequent infection.

Furthermore, alanine scanning highlighted some relevant RBD residue recognized by KBA2401. Some of those, i.e., P426 and I468, also affected Pemivibart binding to RBD (**Supplementary Figure S3**), revealing that KBA2401 and Pemivibart epitope are partially overlapping. A recent study has shown that Pemivibart has a 6.6-fold reduced potency against JN.1 sublineage variants, such as KP.3 (19). We see a similar drop in potency of Pemivibart in our Pseudovirus assays (**Figure 3D**). In contrast, KBA2401 and selected EVO variant retain full potency against KP.3 indicating that the KBA2401 epitope is in fact more conserved than the epitope of the current therapeutic antibody, Pemivibart.

### Combining directed evolution of immortalized B cells and antibody engineering to combat viral escape variants

The ability of stably immortalized B cell clones to undergo AID-induced somatic hypermutation allows *ex vivo* directed evolution of the clones, mimicking the counter-evolution function of the immune system *in vitro*. In this evolution algorithm, the B cell clones were generating sequence diversity in the BCRs, while the selective pressure was applied through our screening methods focused on SARS-CoV-2 binding and neutralization properties. In both *ex vivo* B cell directed evolution campaigns, we identified mutations beyond CDR loops, indicating potential stabilization contributions from residues in the framework regions, which would have been hard to explore by rational design or conventional affinity maturation approaches. Recently, a phenomenon termed ‘anticipatory memory’ was described, wherein SHM in B cell clonotypes generate antibodies that show neutralizing capabilities against viral variants that have not yet emerged (20, 21). An interesting approach would be to mimic anticipatory memory by monitoring the evolution of the immortalized B cells *in vitro*, this approach may shed light on whether this process is truly stochastic or that there are certain variable gene families more inclined to participate in anticipated memory (21). Additional strategies have been proposed to combat viral escape by addressing the narrow epitope specificity of monoclonal antibodies (22). One promising strategy in therapeutic applications is to combine neutralizing and non-neutralizing monoclonal antibodies. Such combinations, including bispecific antibodies, can broaden the spectrum of protection by additionally utilizing Fc-mediated effector functions (22), and by targeting multiple conserved sites these approaches enhance resilience of therapeutics against emerging variants. Indeed, to this end we investigated the possibility of a bi-paratopic antibody including KBA2401 (1B10), evolved for higher neutralization potency, and KBA2402 (1E7) for increased binding capacity towards new escape variants. By combining the resulting antibodies from our selection and evolution campaigns, we generated a bi-paratopic molecule with both broad neutralization properties and pan-variant binding ability.

In conclusion, we report on the use of B cell immortalization for the comprehensive functional screening of the human B cell repertoires from PBMC and tonsil tissue to produce valuable insights on the immune response to the rapidly evolving SARS-CoV-2 virus. We describe how combining immortalized B cell screening with directed B cell evolution and antibody engineering offers a powerful workflow to enable rapid response to evolving pandemic threats. Furthermore, the B cell technology has proven to be a powerful tool to discover functional antibodies and have insights on *in vivo* accessible, *non-self* epitopes. High efficiency immortalization and functional screening of B cells combined with directed evolution and protein engineering can accelerate the discovery of new therapeutics and provides an effective means to counter act the emergence of immune escape variants.

## Data Availability

The raw data supporting the conclusions of this article will be made available by the authors, upon direct request to corresponding author.

## Supplementary material

Supplementary Figure 1 Ratio of median fluorescence intensity (MedFI) from PBMC-derived (green) and tonsil-derived (red) minipools plotted by descending signal, measured by flow cytometry. A total of 7680 antibody-containing supernatants were screened for binding. Hits were defined based on signal thresholds relative to background, yielding 123 hits from tonsil libraries and 474 from PBMC libraries. Of these, 440 minipools were selected for downstream live virus neutralization assays. Bebtelovimab (blue dot) at 1 μg/mL served as a positive control.

Supplementary Figure 2 (A) Live virus neutralization curves of KBA2401, KBA2402, KBA2403, KBA2404 and Bebtelovimab against live SARS-CoV-2 variants WT (blue line), Alpha (red line), Delta (green line), BA.2 (purple line), BA.2.75 (orange line), BA.5.5 (black line), XBB (brown line) and XBB.1.5 (dark blue line). (B) ELISA evaluation of KBA2401, KBA2402 and Bebtelovimab binding to SARS-CoV-2 Delta RBD (red) or Spike trimer (green). KBA2401 and Bebtelovimab show binding curves to both proteins while KBA2402 only shows binding to trimer.

Supplementary Figure 3 ELISA evaluation of Pemivibart binding to spike RBD wild type and Alanine-mutants. Control experiment for epitope mapping analysis for KBA2401.

Supplementary Figure 4 Live virus neutralization of JN.1 by KBA2401 OC compared to bi-paratopic 2401*OC_2402*EVO14. A) depicts titration neutralization curve while B) depicts the heatmap of IC50s showing a clear improvement of the bi-paratopic construct.

Supplementary Figure 5 List of SDS-Page and SEC results for each antibody production. Includes reference to the main figure where the produced antibody was used.
