# Supplementary figures and images for: Immune counter-evolution: immortalized B cell clones can undergo ex vivo directed evolution to counteract viral escape
Source: Front Immunol. 2025 Aug 18;16:1648717. doi: 10.3389/fimmu.2025.1648717 (PMC12399645; doi:10.3389/fimmu.2025.1648717)

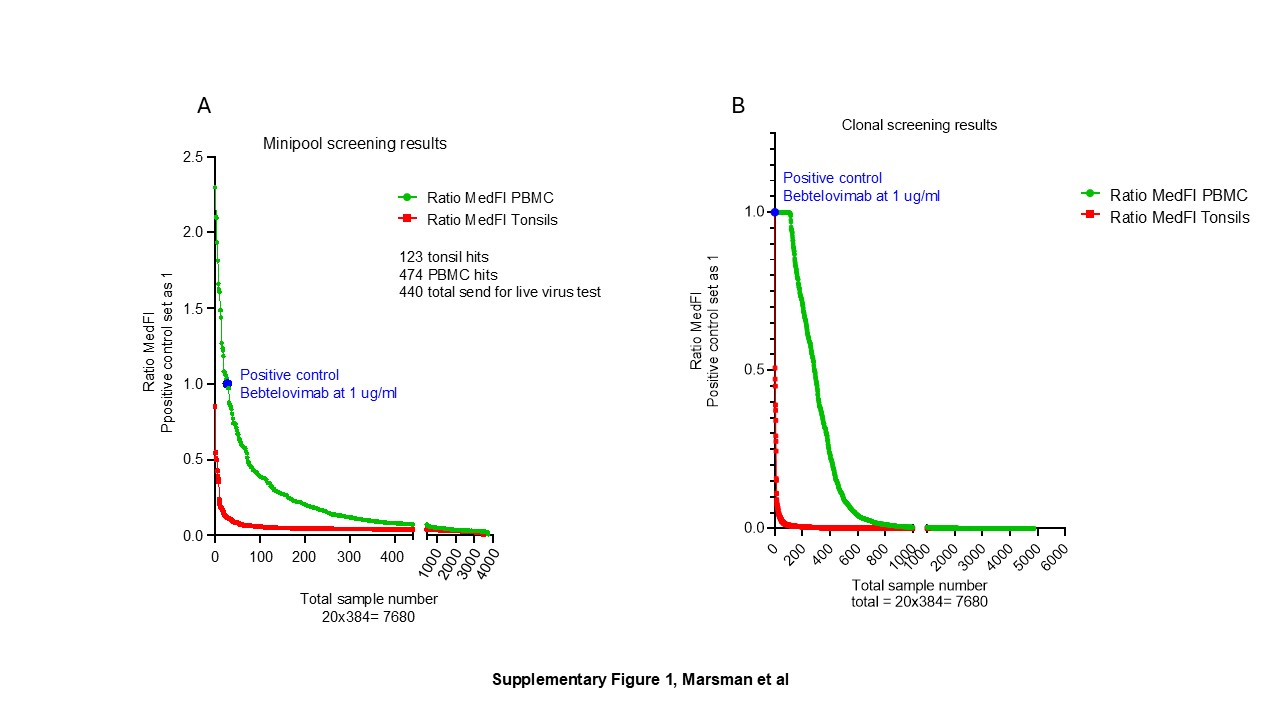

Supplement: Supplementary Figure 1 — Ratio of median fluorescence intensity (MedFI) from PBMC-derived (green) and tonsil-derived (red) minipools plotted by descending signal, measured by flow cytometry. A total of 7680 antibody-containing supernatants were screened for binding. Hits were defined based on signal thresholds relative to background, yielding 123 hits from tonsil libraries and 474 from PBMC libraries. Of these, 440 minipools were selected for downstream live virus neutralization assays. Bebtelovimab (blue dot) at 1 μg/mL served as a positive control. [file Image1.jpeg]

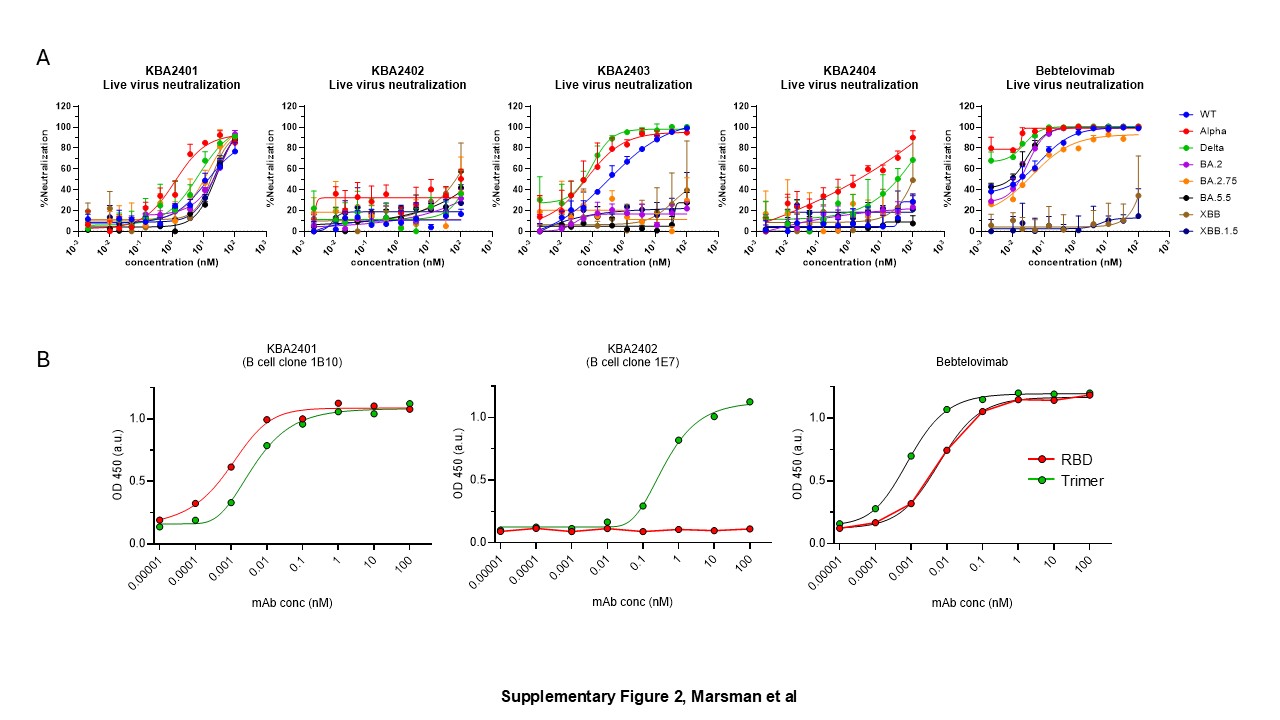

Supplement: Supplementary Figure 2 — (A) Live virus neutralization curves of KBA2401, KBA2402, KBA2403, KBA2404 and Bebtelovimab against live SARS-CoV-2 variants WT (blue line), Alpha (red line), Delta (green line), BA.2 (purple line), BA.2.75 (orange line), BA.5.5 (black line), XBB (brown line) and XBB.1.5 (dark blue line). (B) ELISA evaluation of KBA2401, KBA2402 and Bebtelovimab binding to SARS-CoV-2 Delta RBD (red) or Spike trimer (green). KBA2401 and Bebtelovimab show binding curves to both proteins while KBA2402 only shows binding to trimer. [file Image2.jpeg]

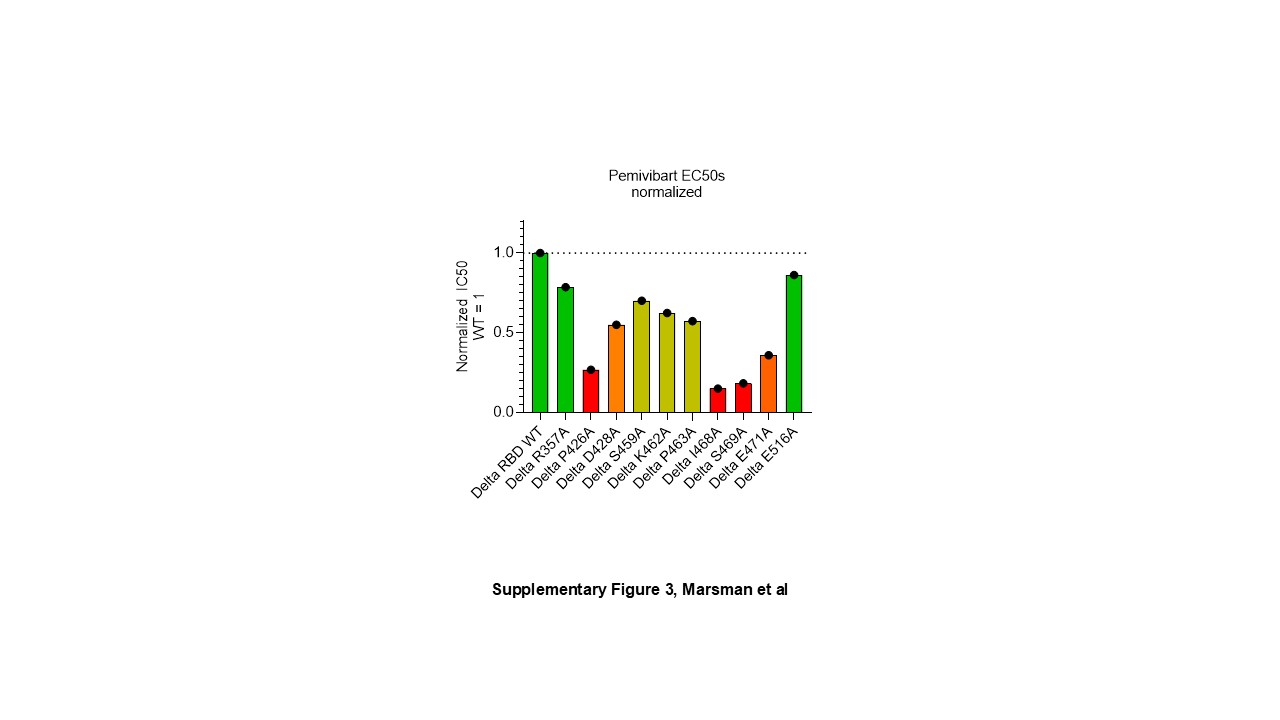

Supplement: Supplementary Figure 3 — ELISA evaluation of Pemivibart binding to spike RBD wild type and Alanine-mutants. Control experiment for epitope mapping analysis for KBA2401. [file Image3.jpeg]

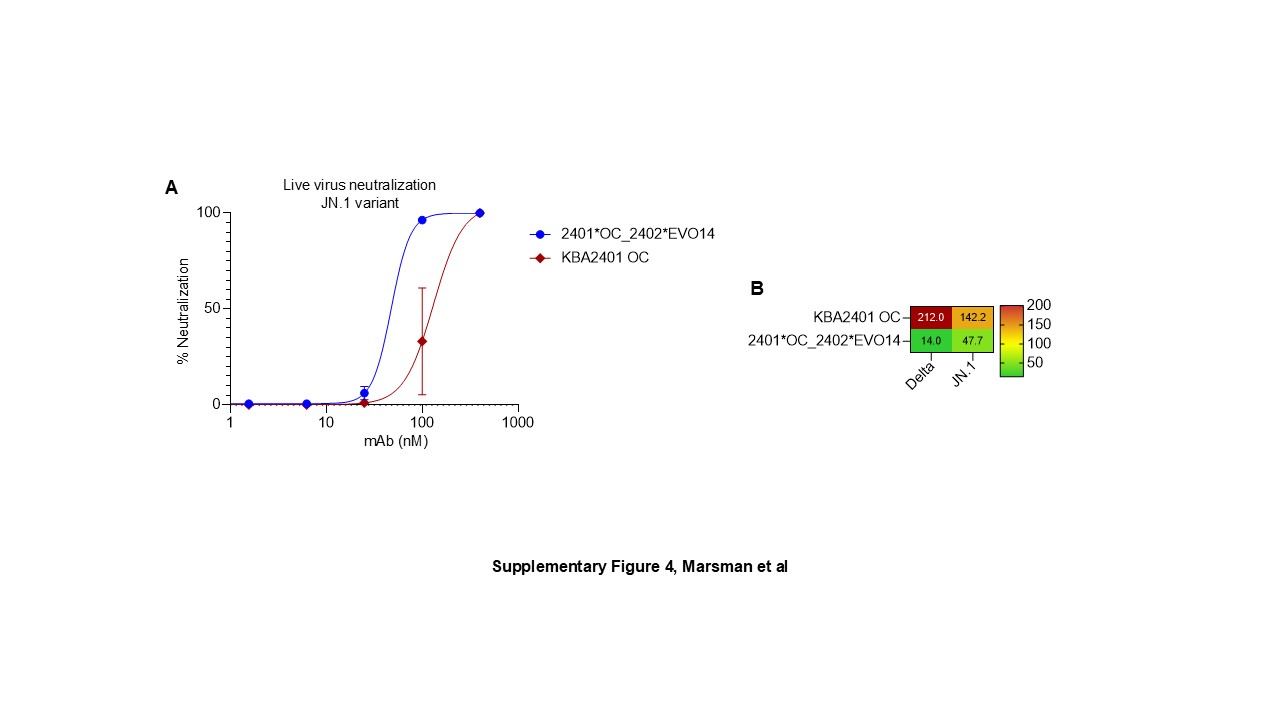

Supplement: Supplementary Figure 4 — Live virus neutralization of JN.1 by KBA2401 OC compared to bi-paratopic 2401*OC_2402*EVO14. A) depicts titration neutralization curve while B) depicts the heatmap of IC50s showing a clear improvement of the bi-paratopic construct. [file Image4.jpeg]
