# Supplementary material for: Immune counter-evolution: immortalized B cell clones can undergo ex vivo directed evolution to counteract viral escape
Source: Front Immunol. 2025 Aug 18;16:1648717. doi: 10.3389/fimmu.2025.1648717 (PMC12399645; doi:10.3389/fimmu.2025.1648717)

KBA2401 (Figure 2)

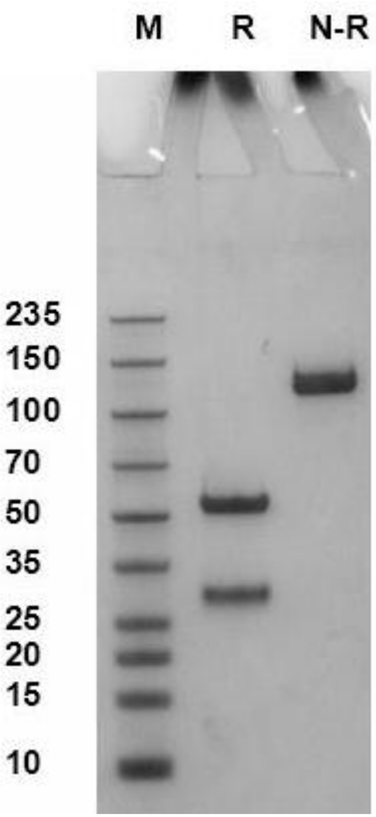

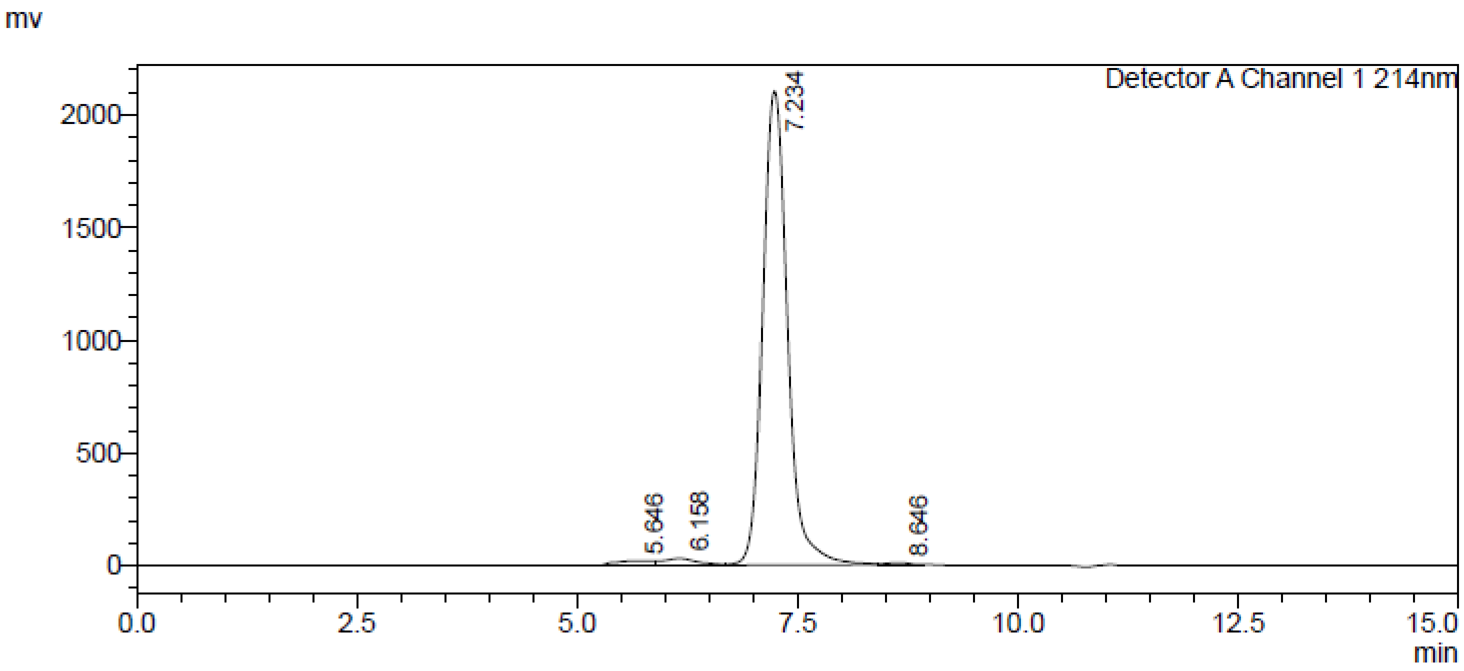

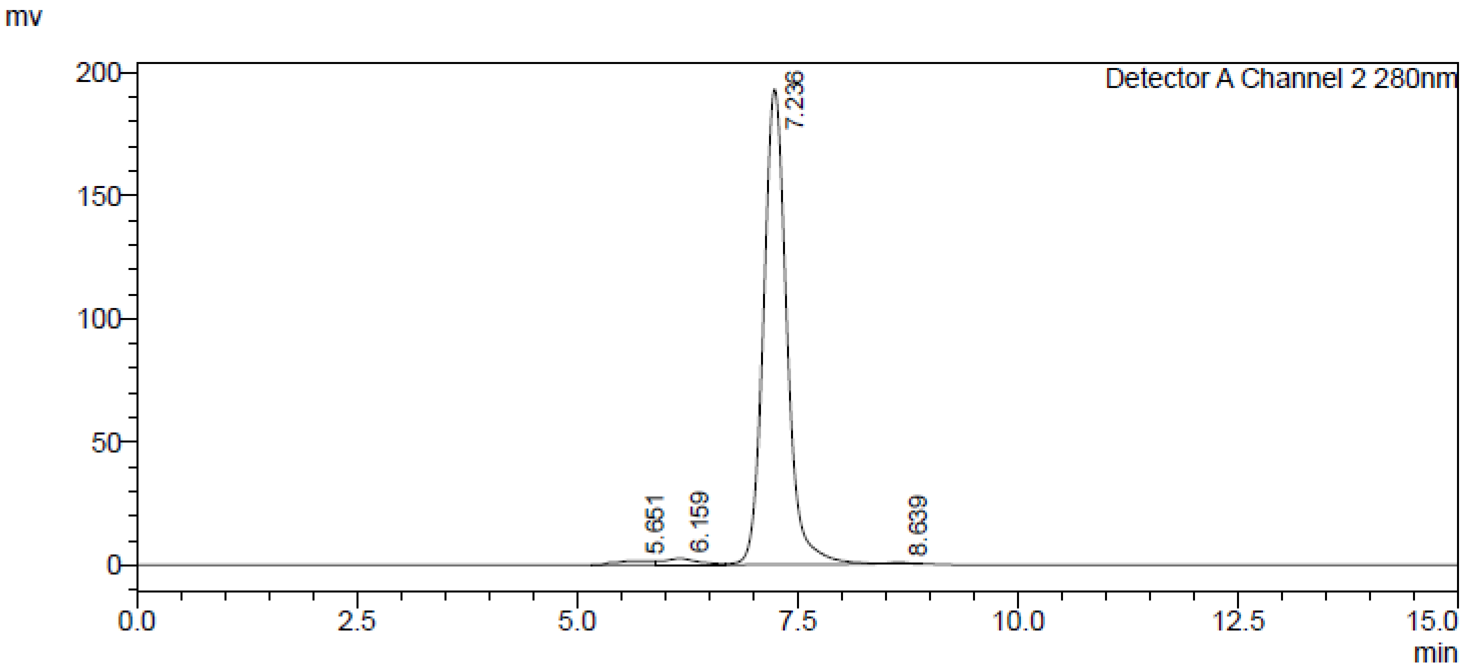

KBA2402 (Figure 2)

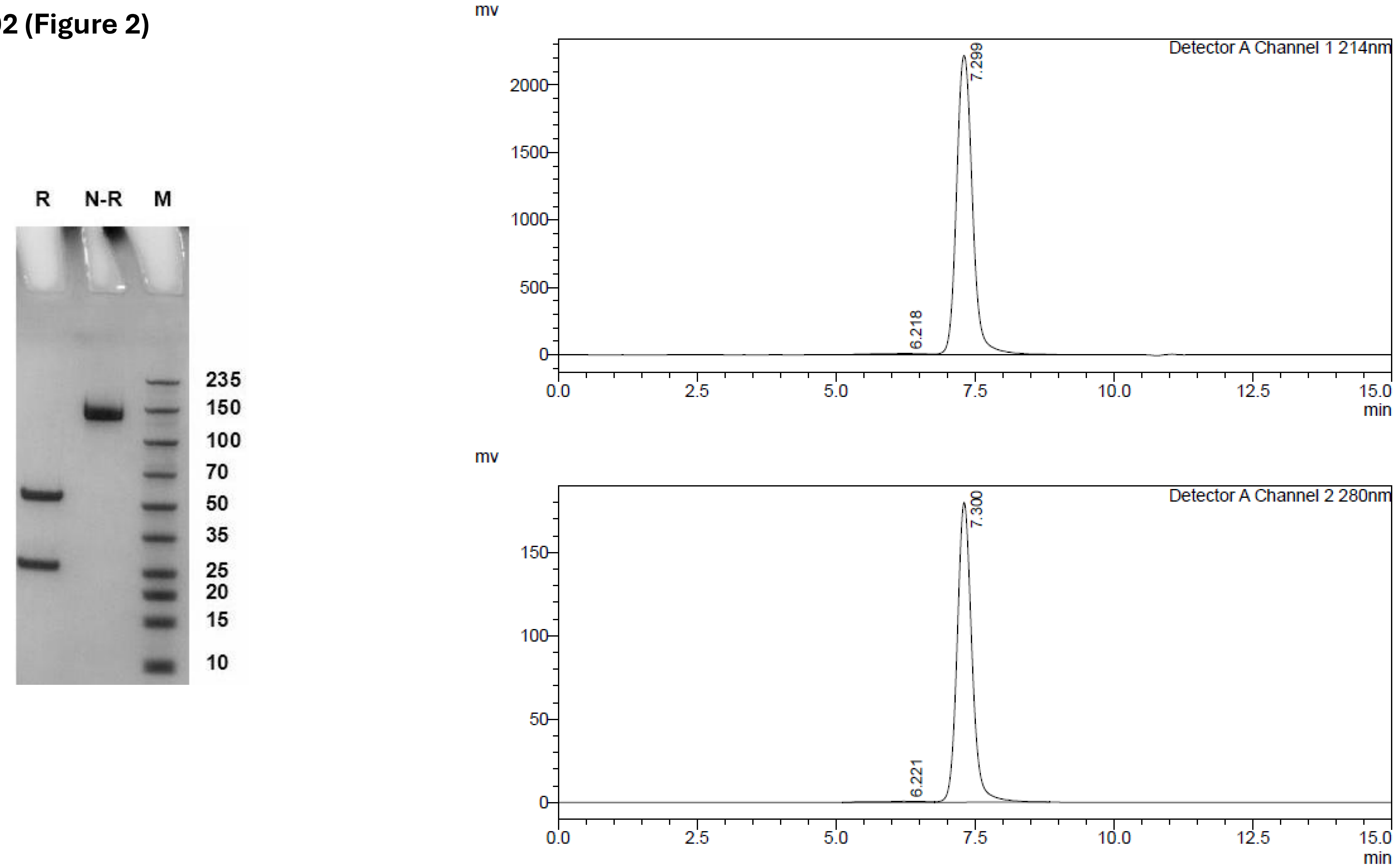

KBA2403 (Figure 2)

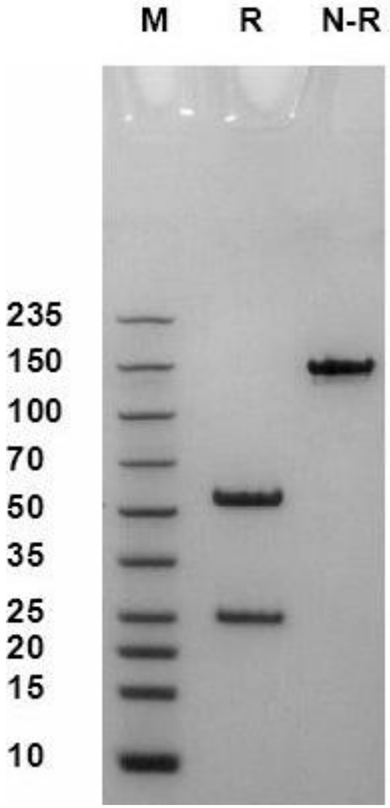

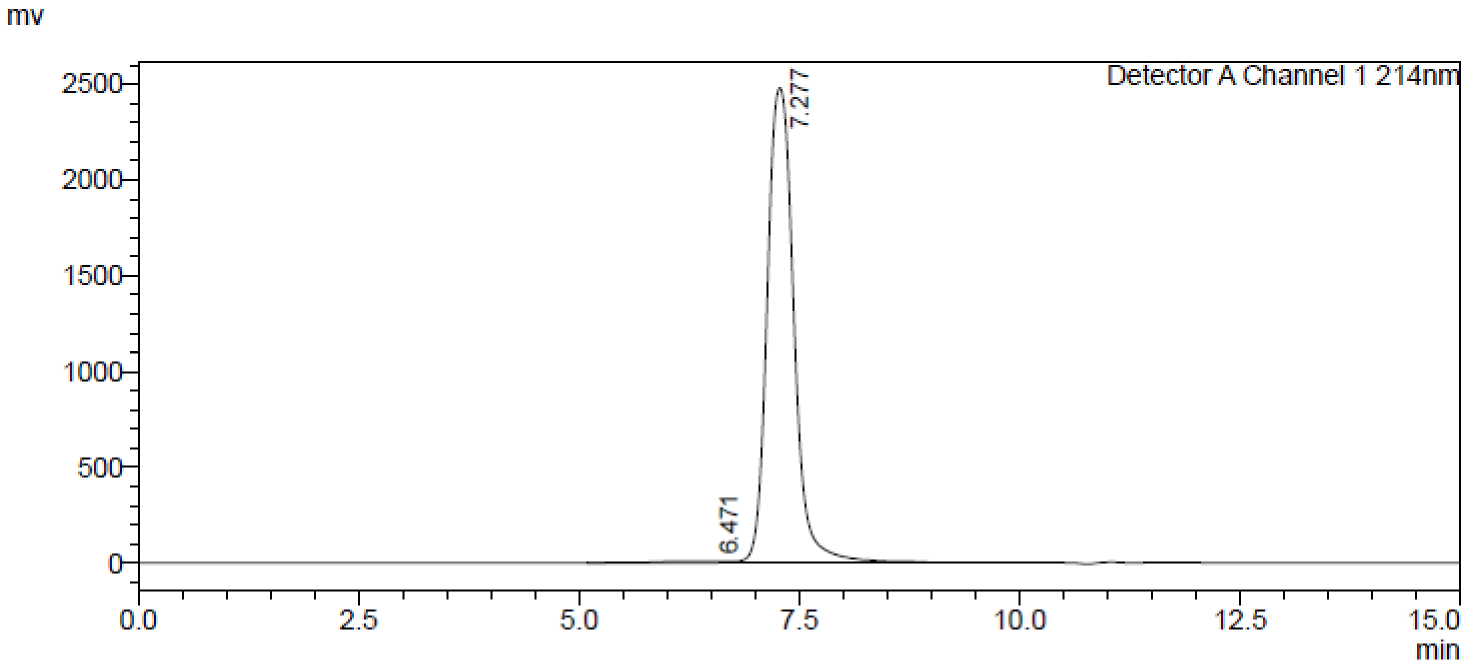

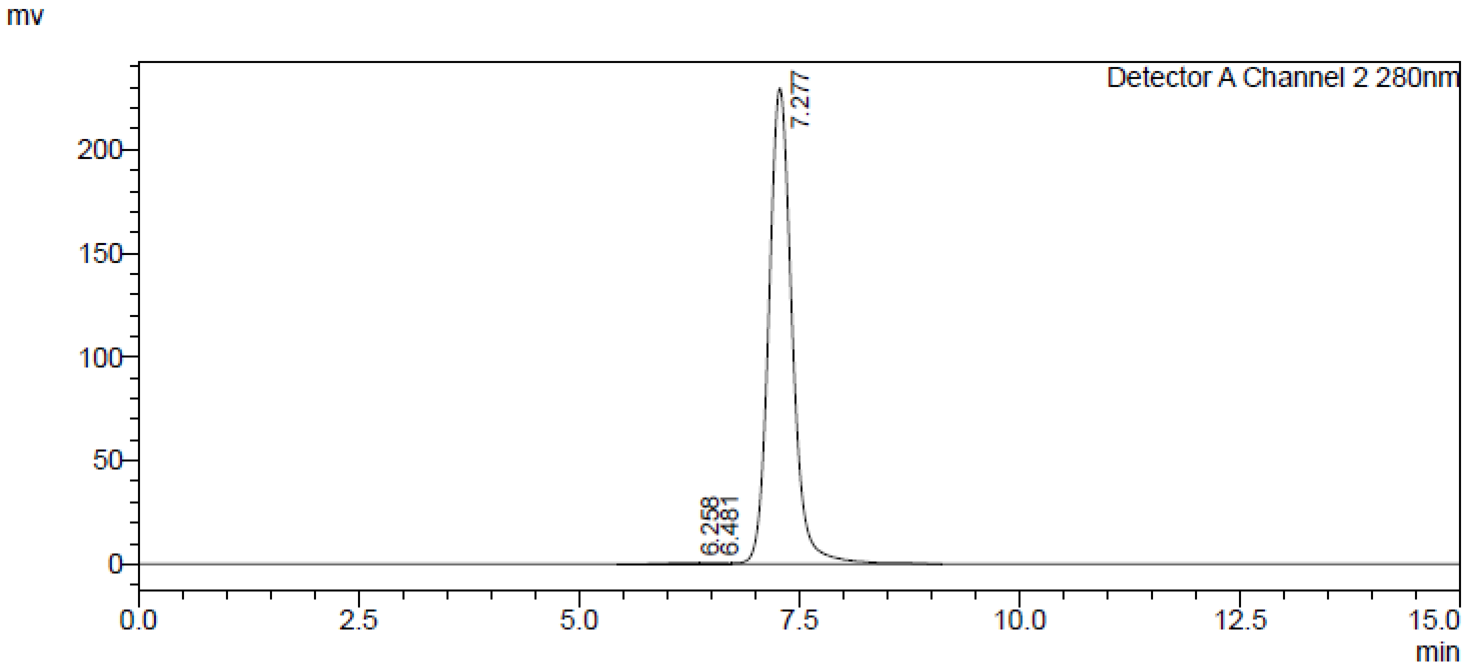

KBA2404 (Figure 2)

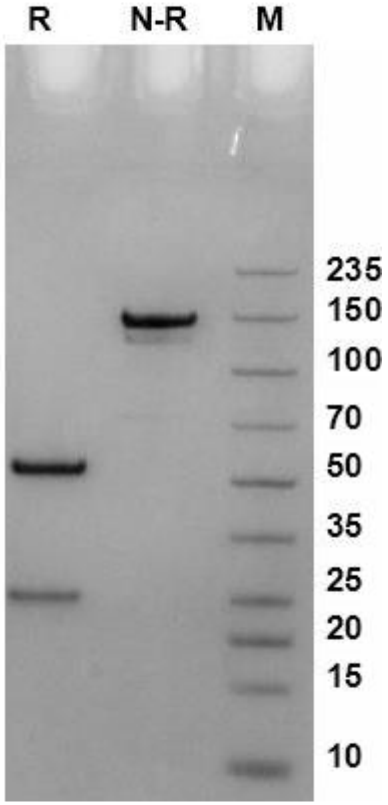

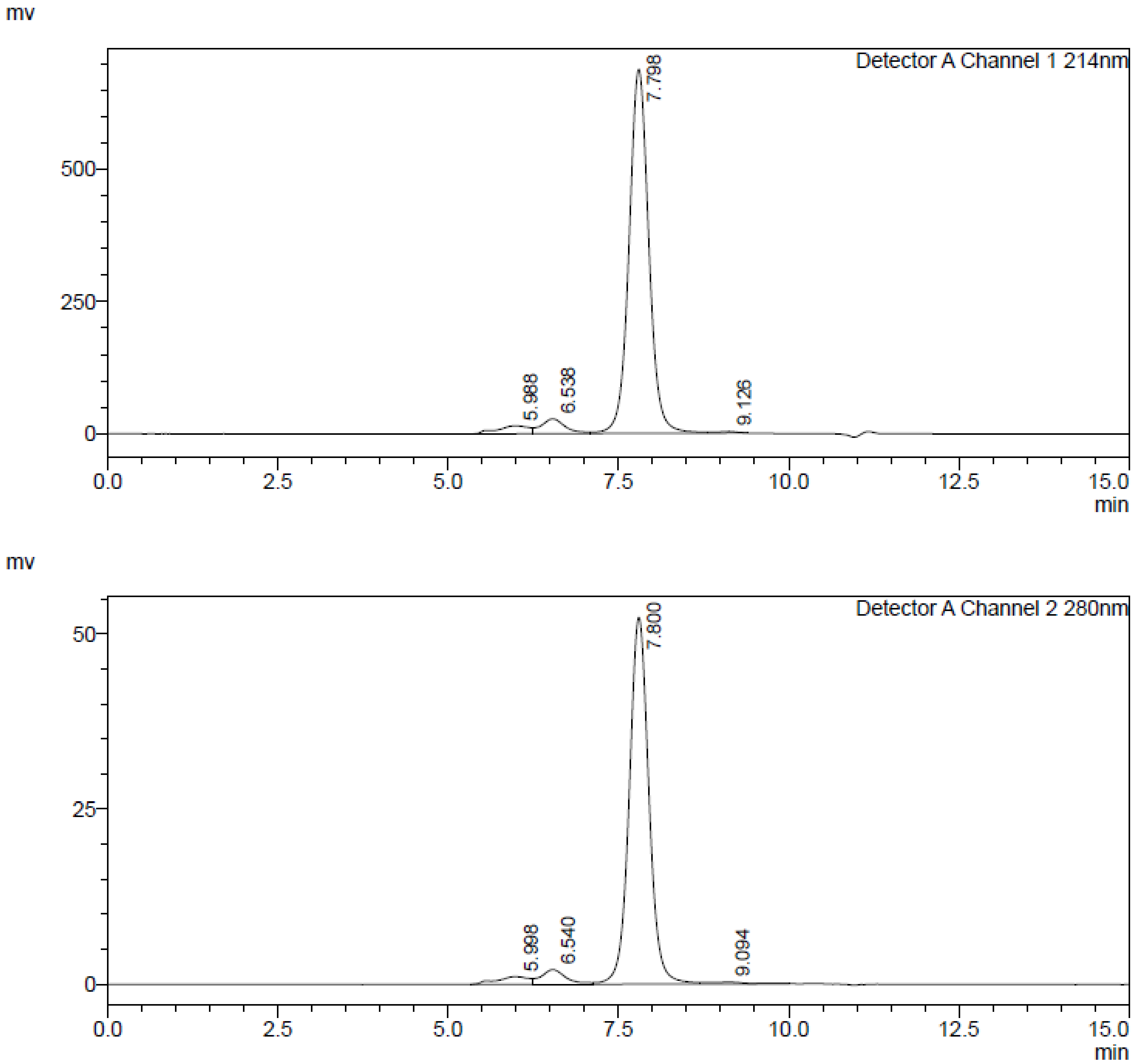

KBA2401_EVO03

Figure 3

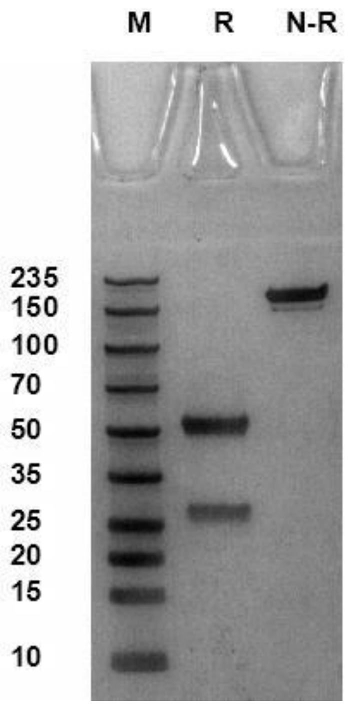

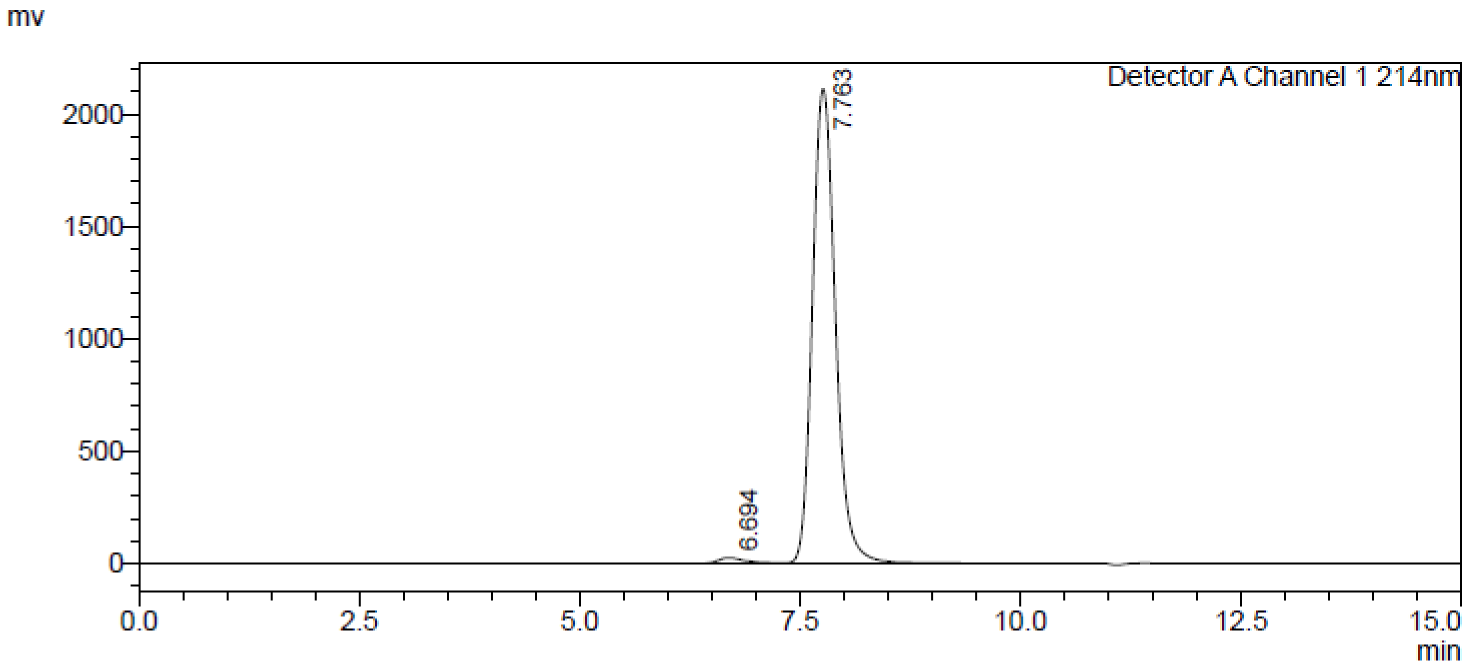

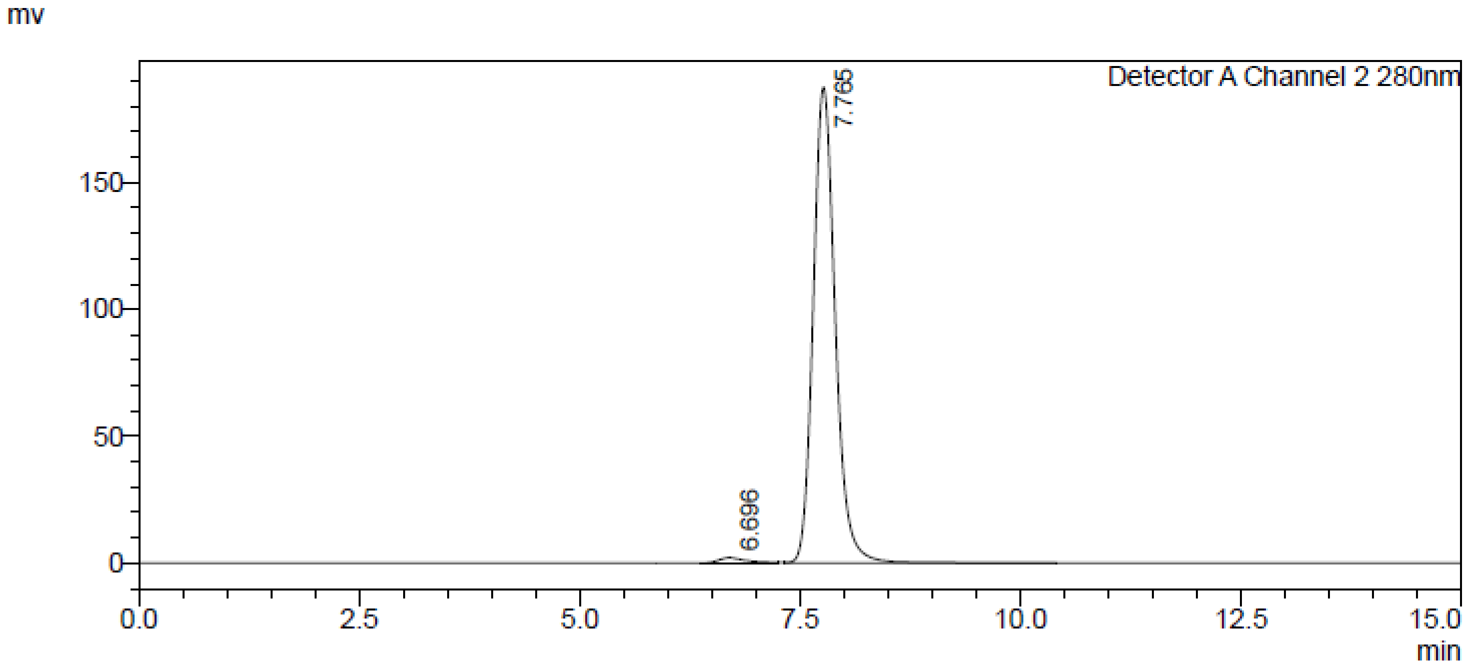

KBA2401_EVO05
Figure 3

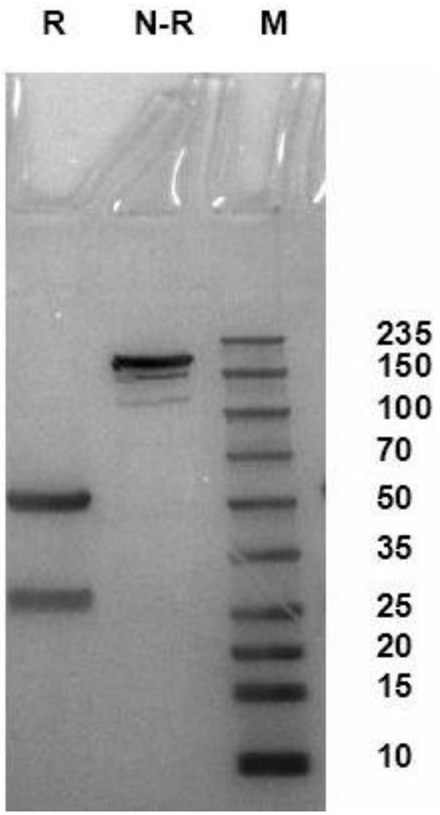

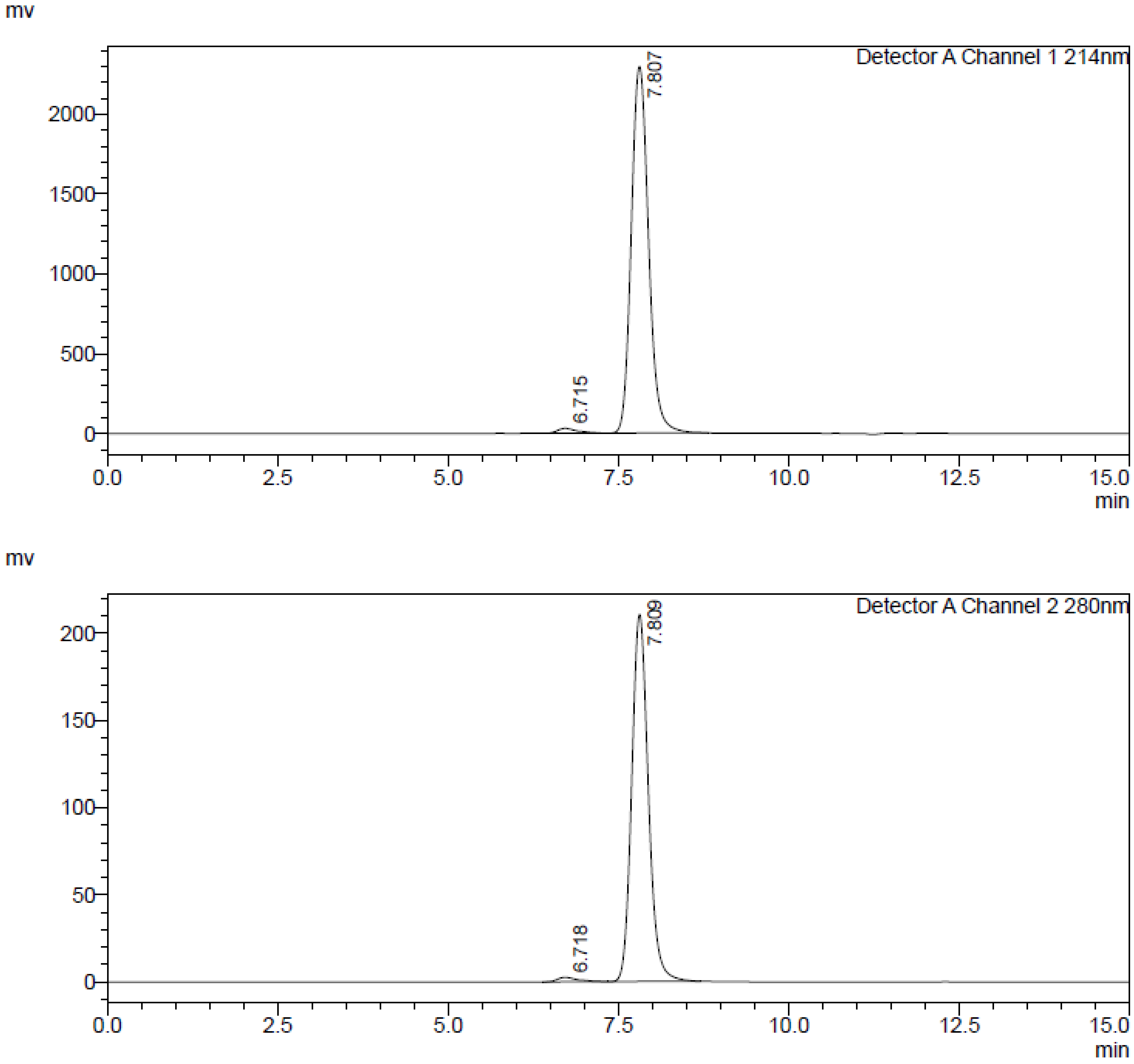

KBA2401_EVO06
Figure 3

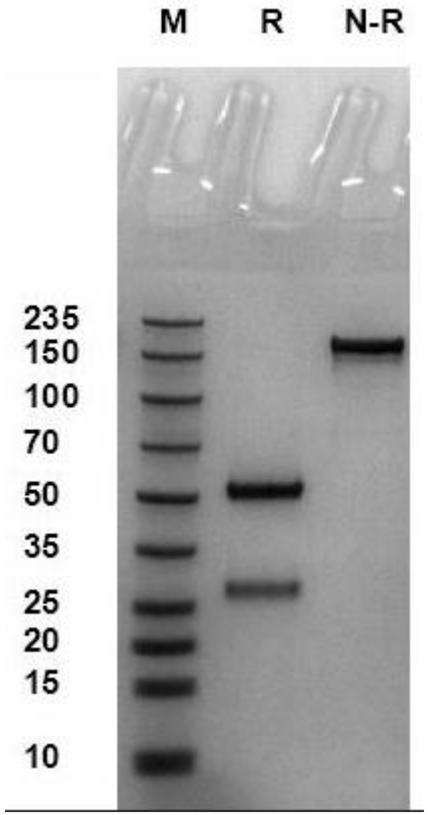

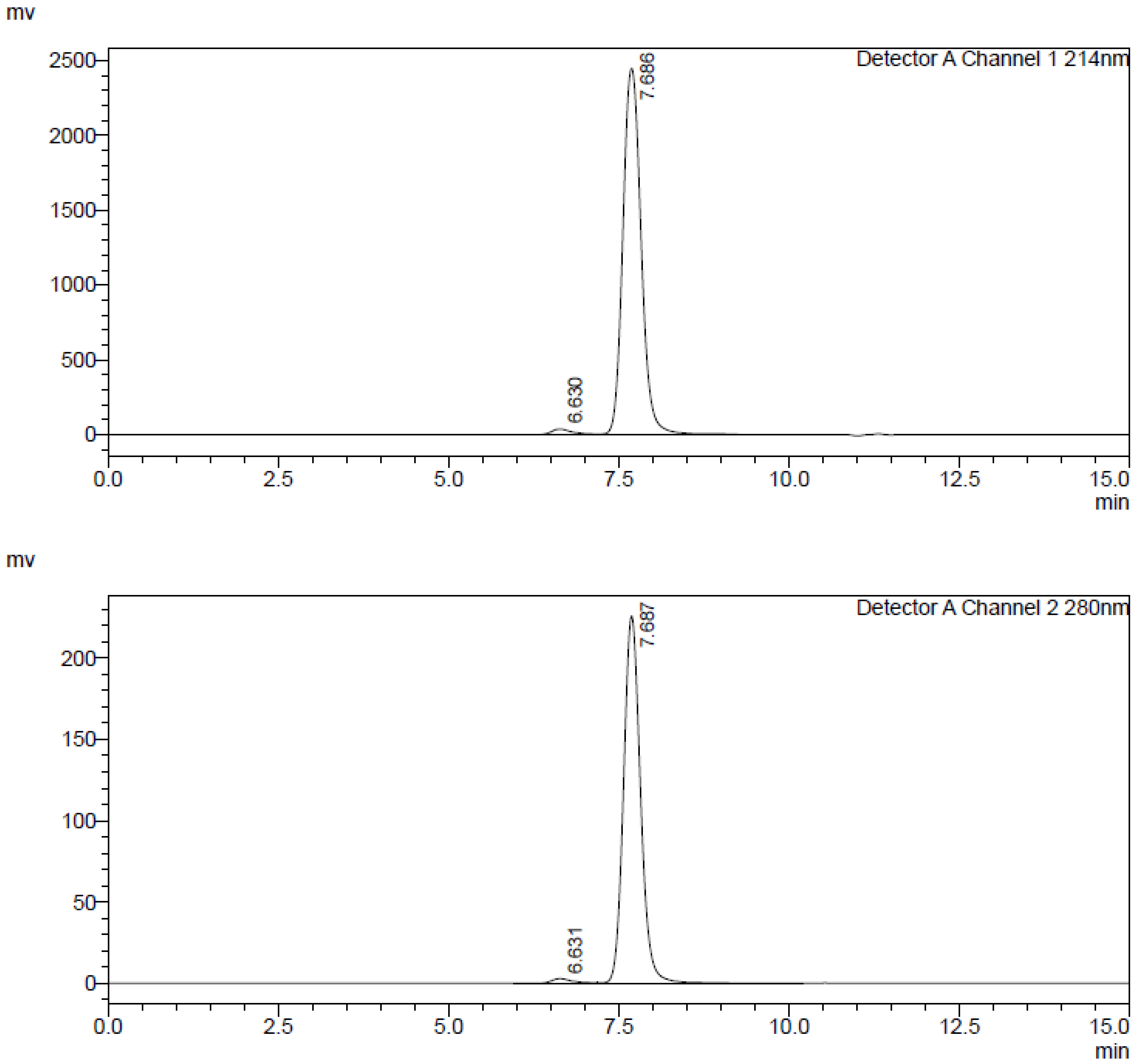

KBA2401_EVO10

Figure 3

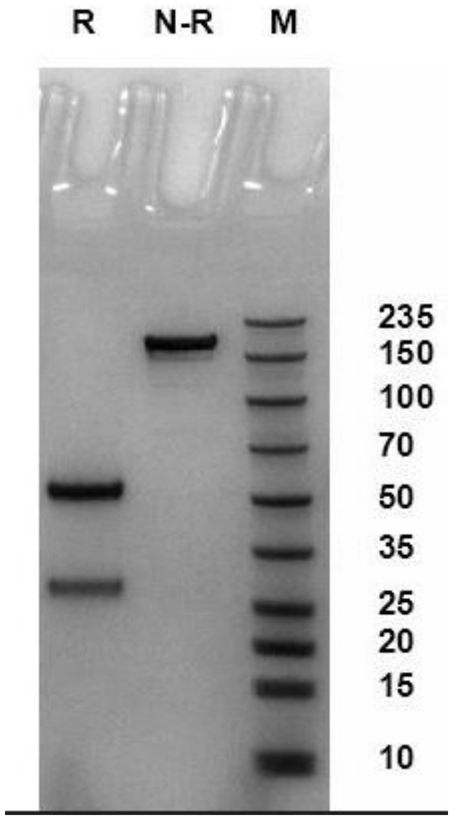

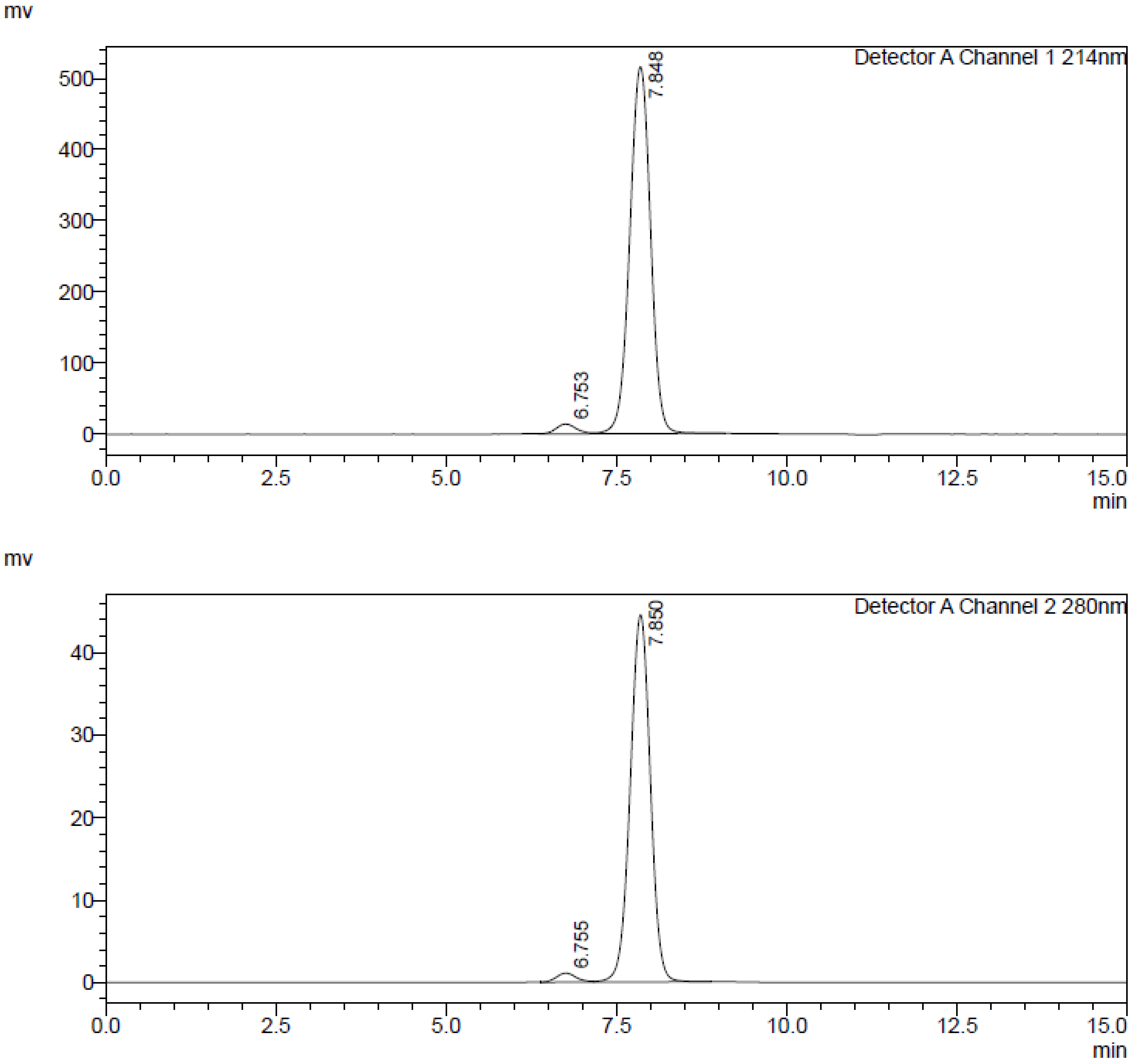

KBA2401_EVO16

Figure 3

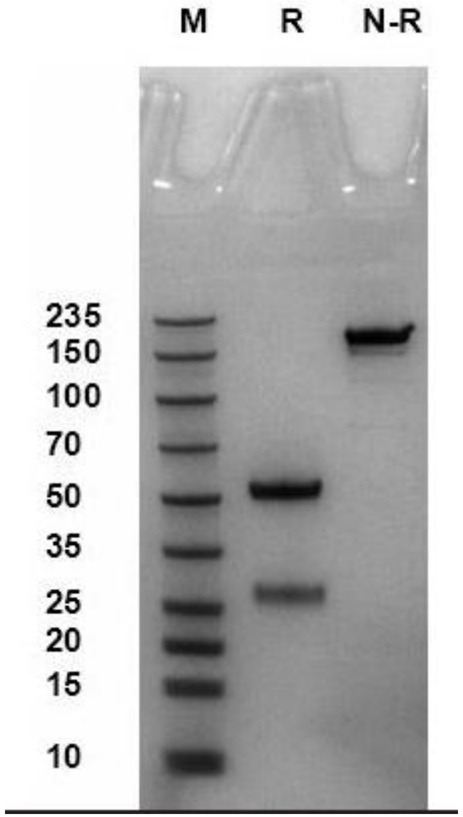

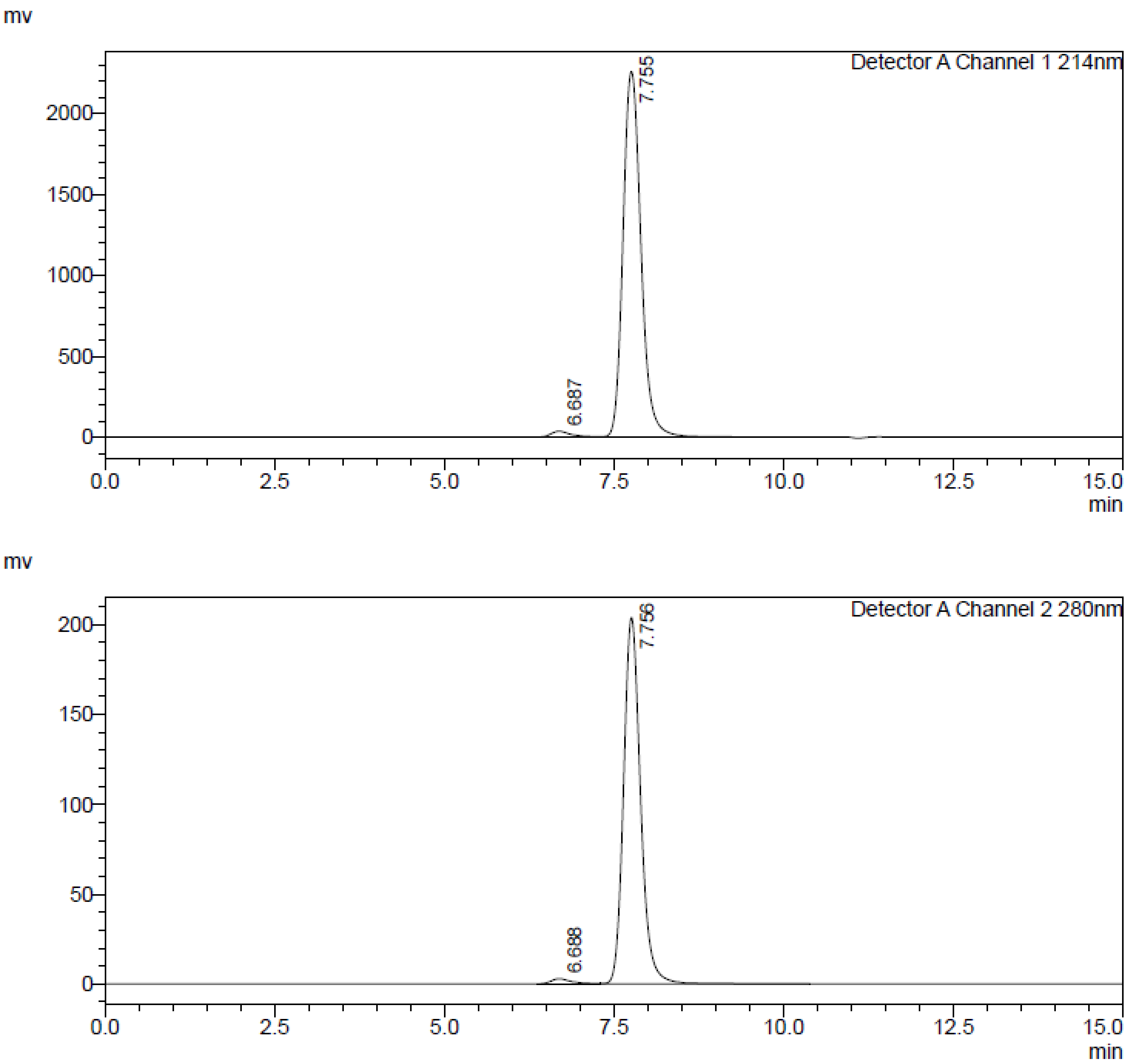

KBA2401_EVO18

Figure 3

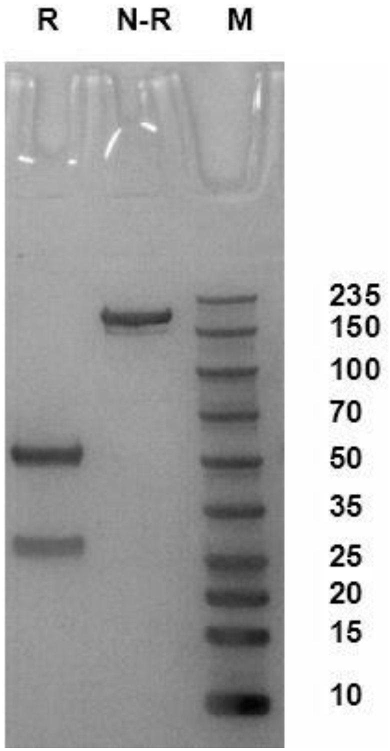

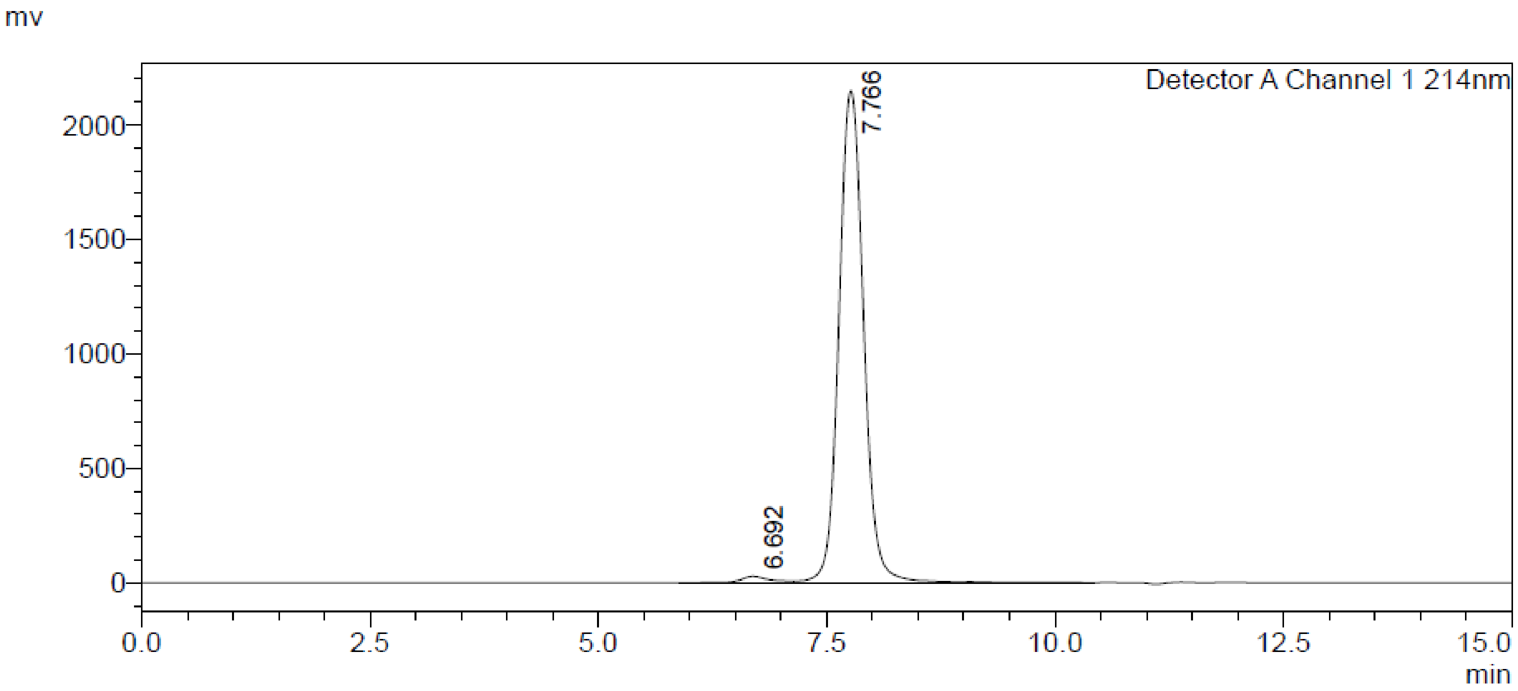

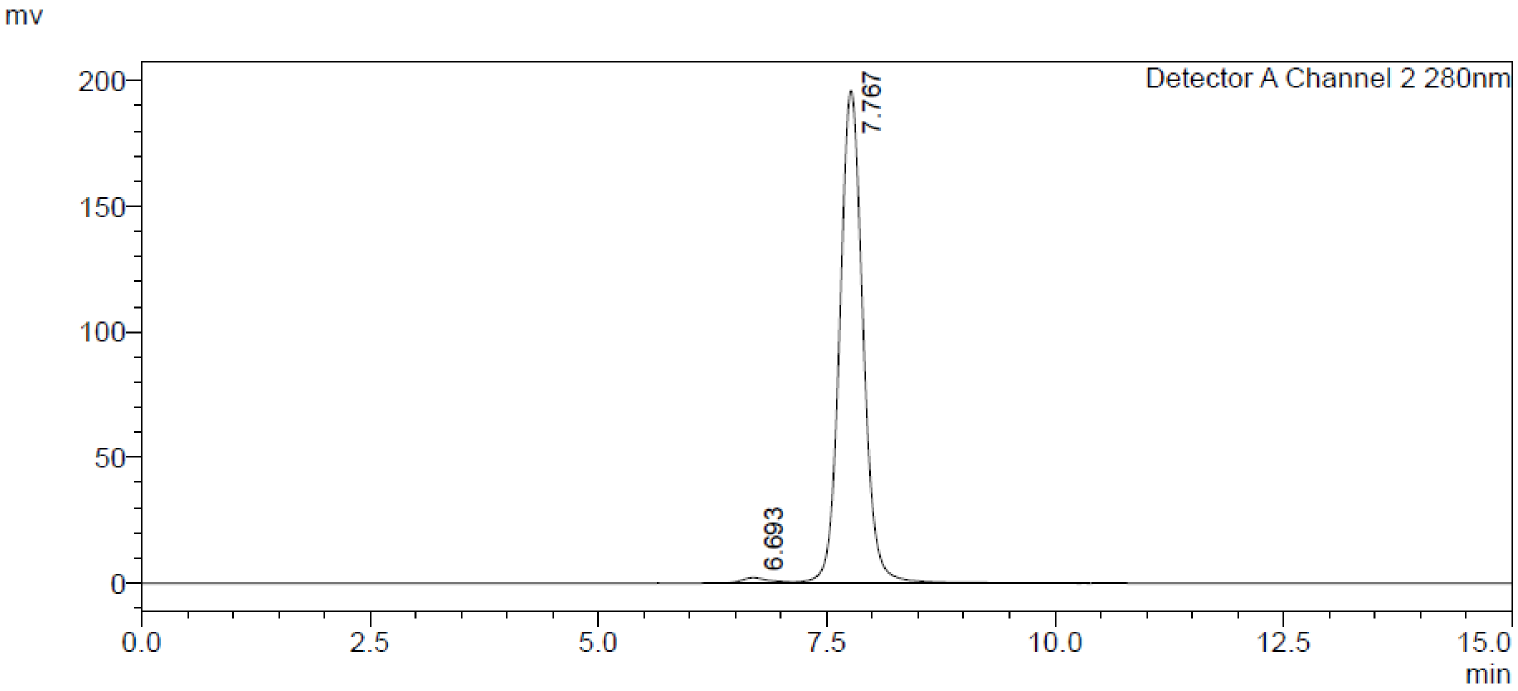

KBA2401_EVO39

Figure 3

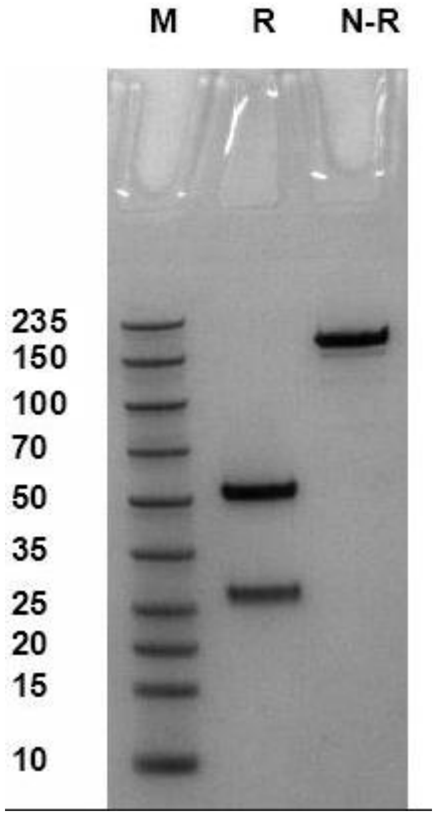

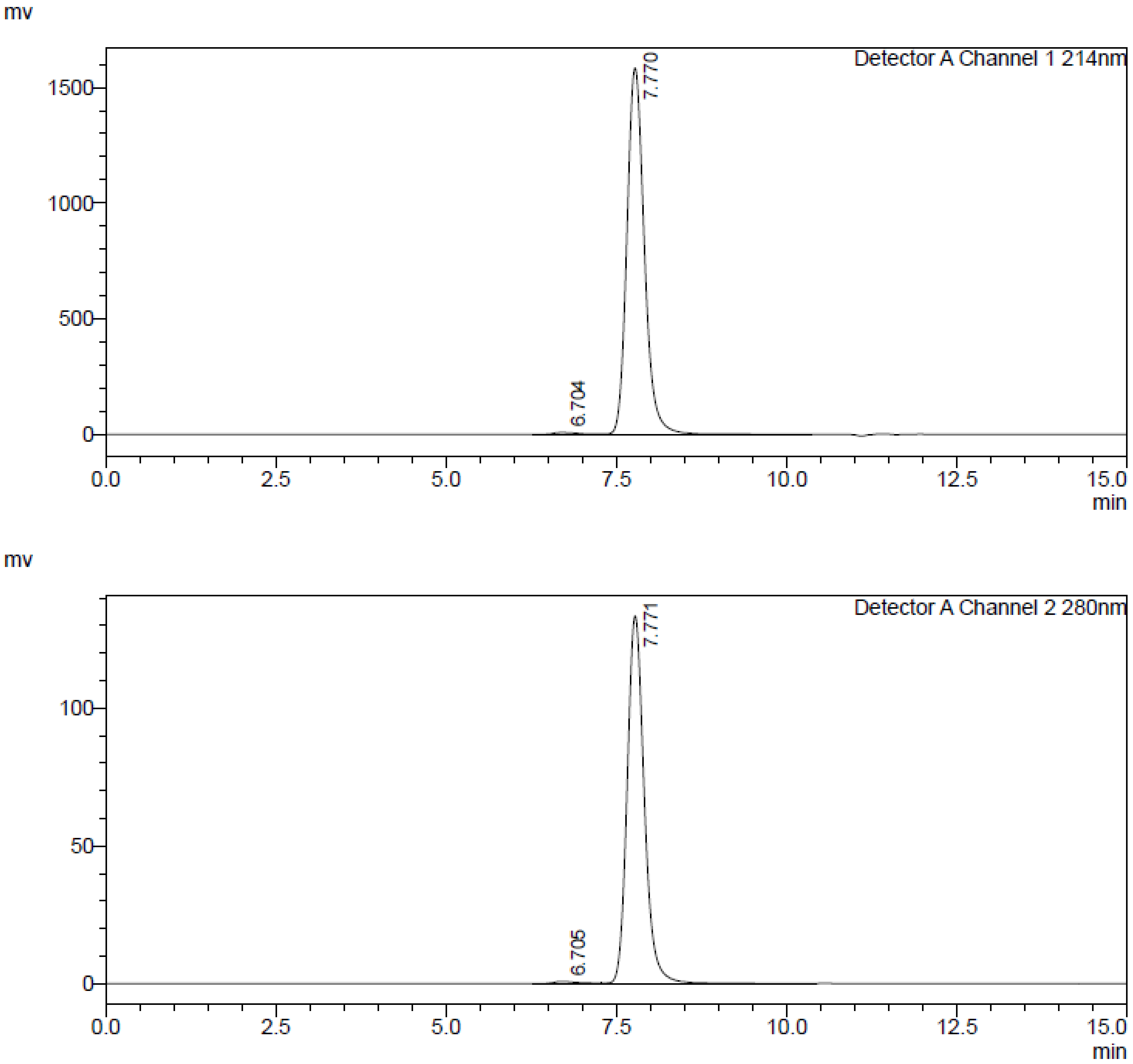

KBA2401_EVO50

Figure 3

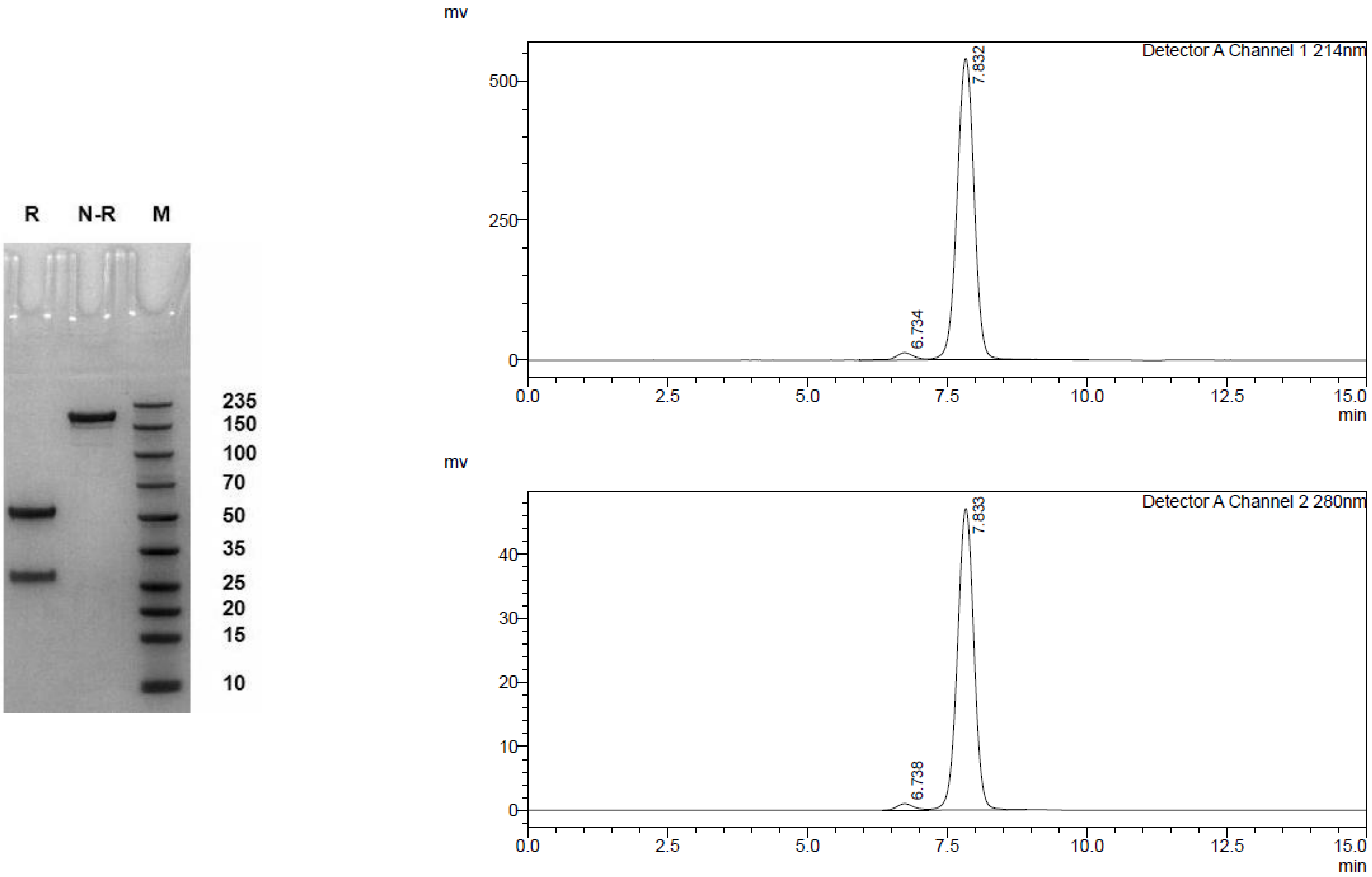

KBA2401_EVO54

Figure 3

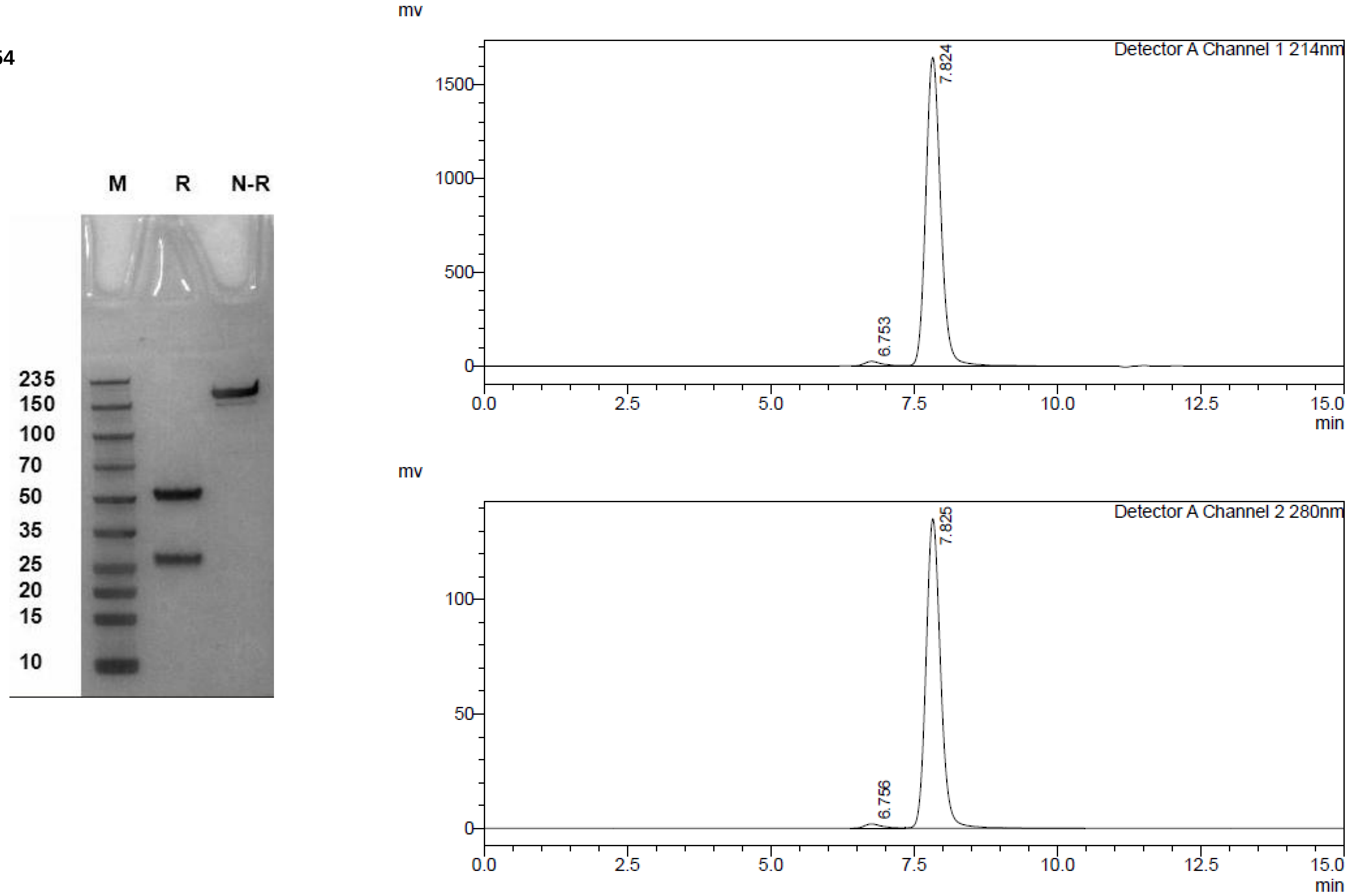

KBA2401_EVO56

Figure 3

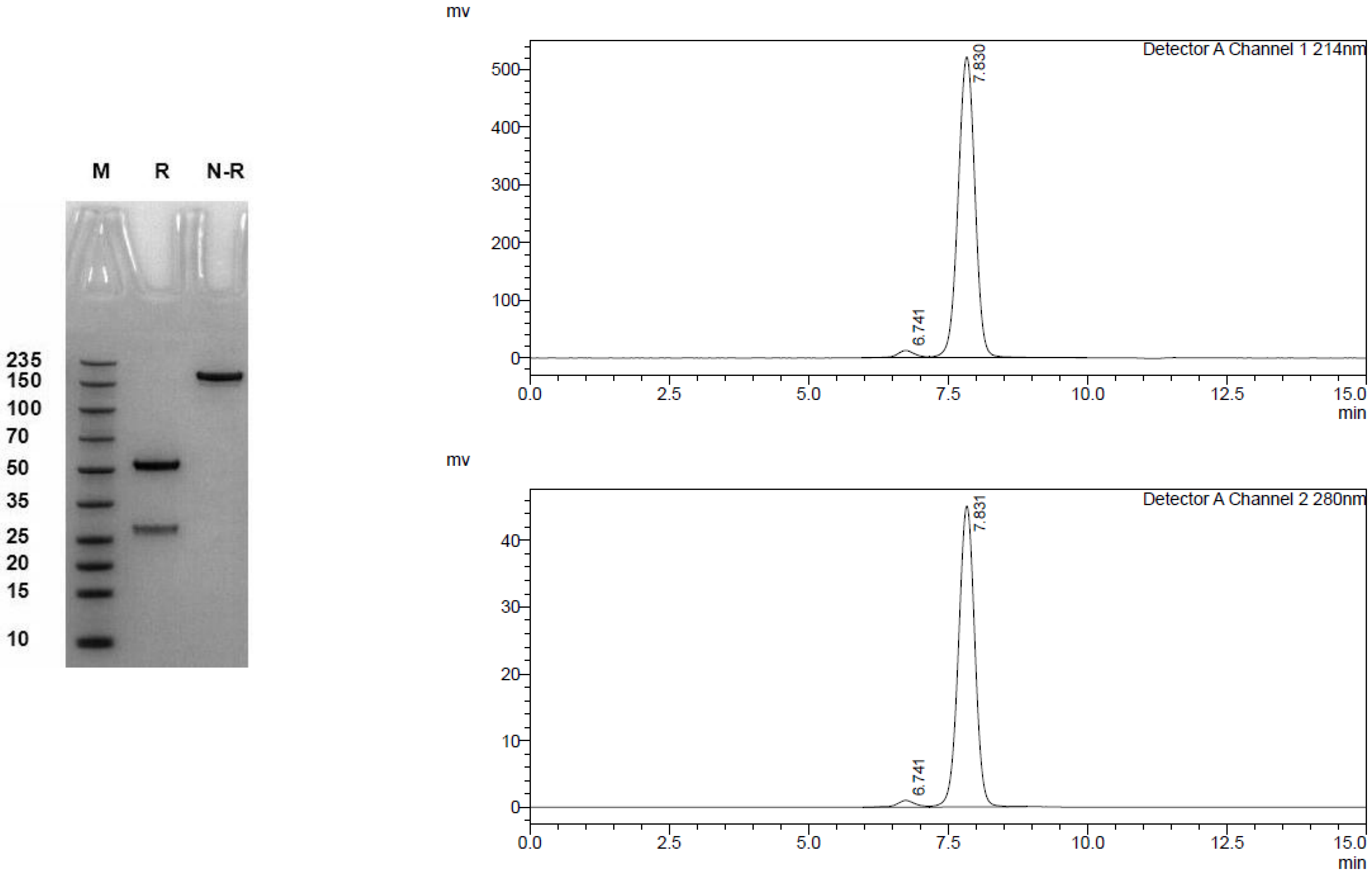

KBA2401_EVO94

Figure 3

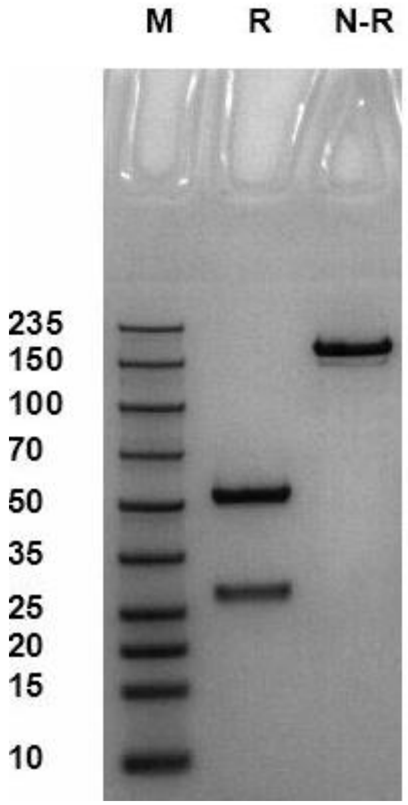

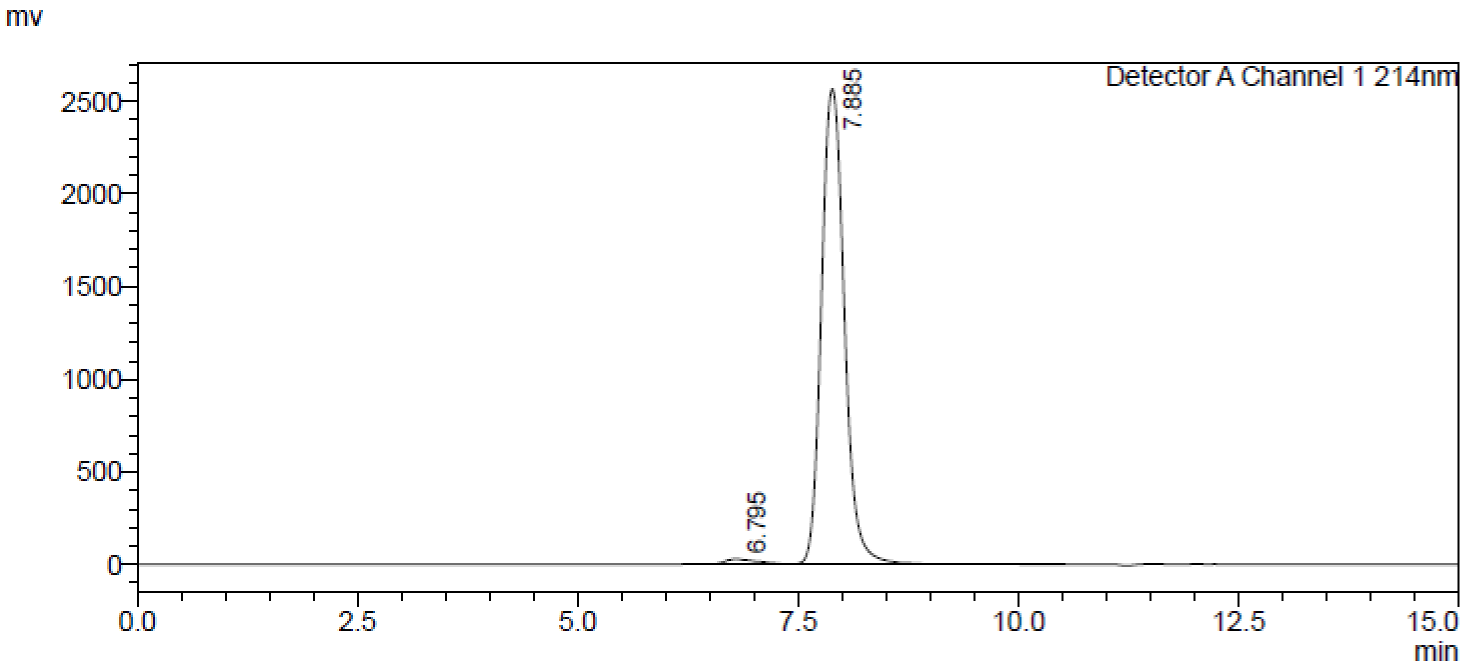

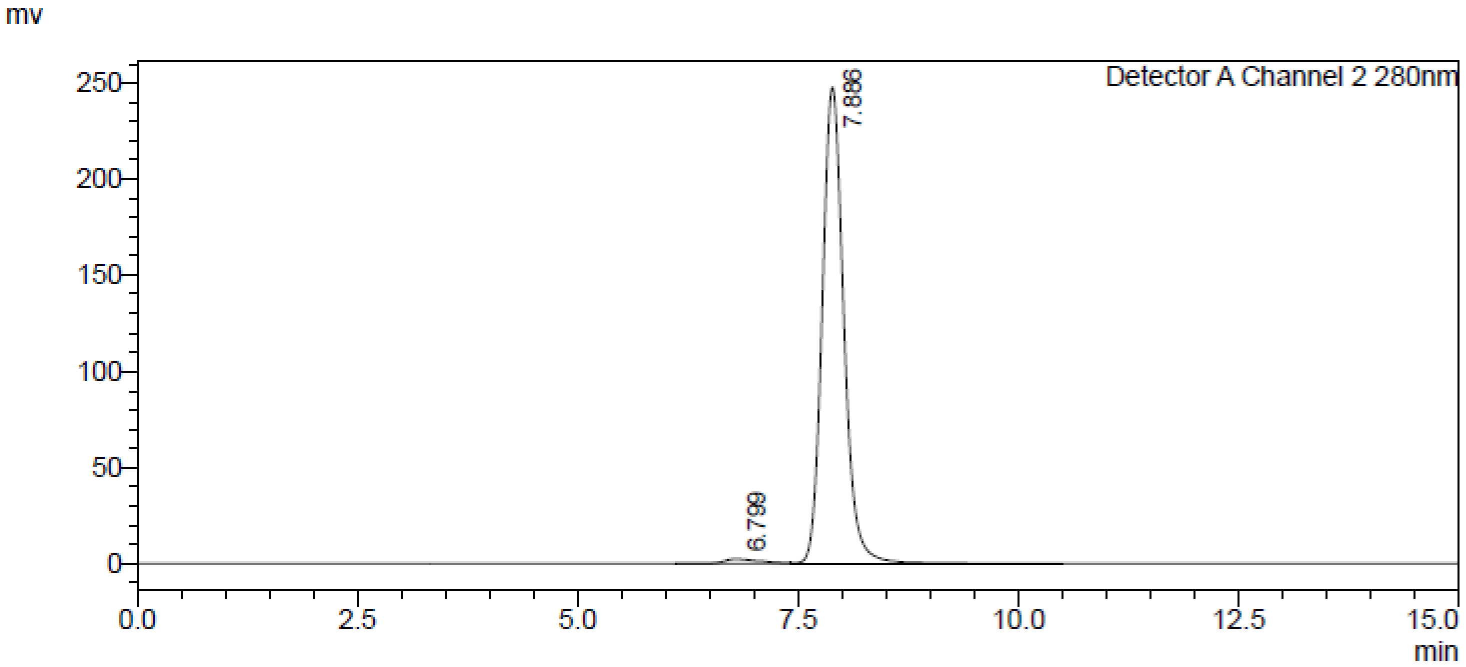

KBA2401_EVO95

Figure 3

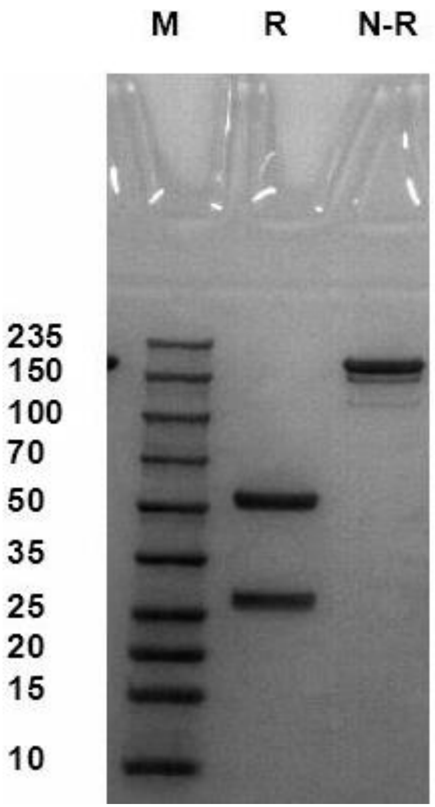

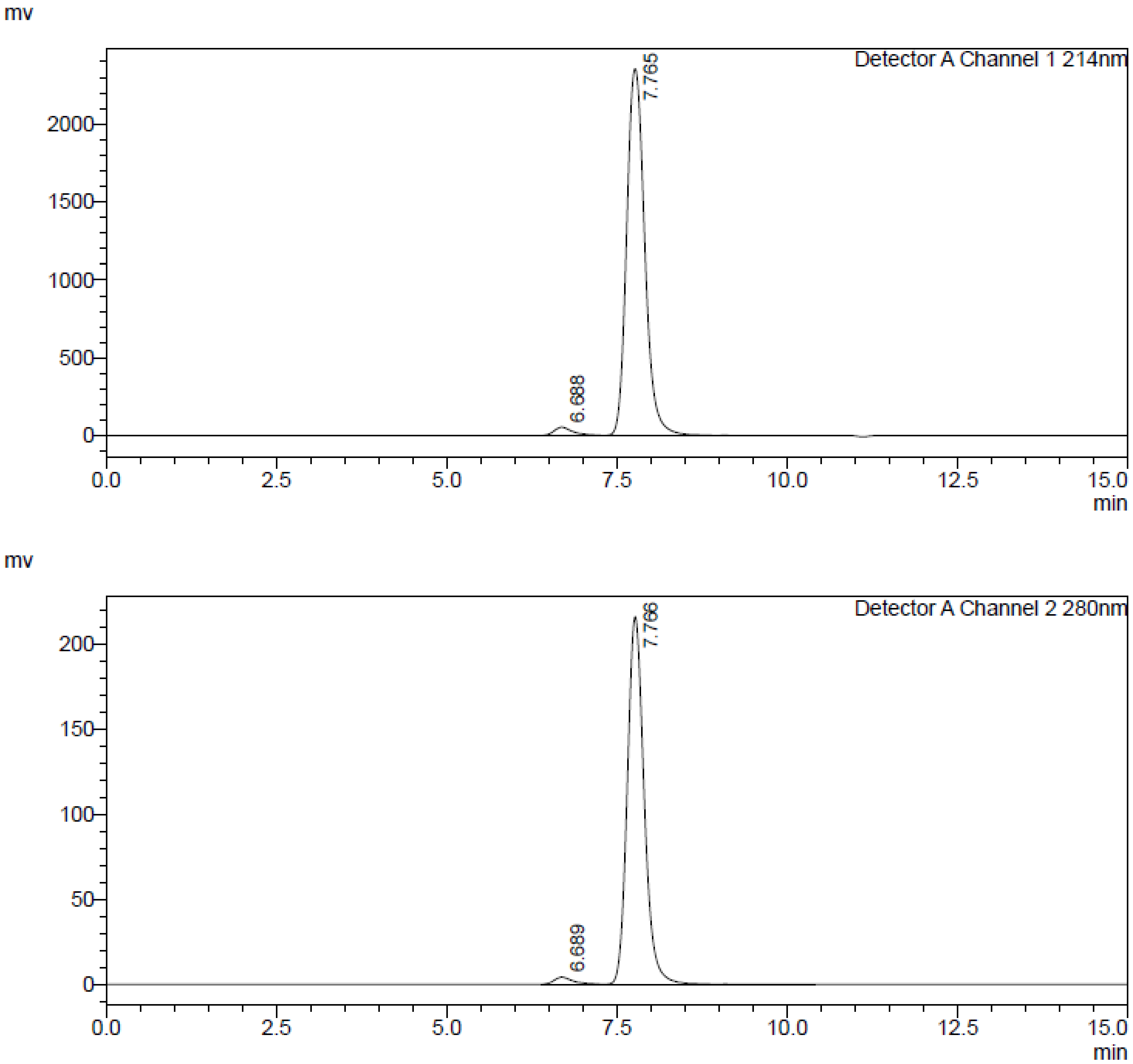

KBA2401_EVO96

Figure 3

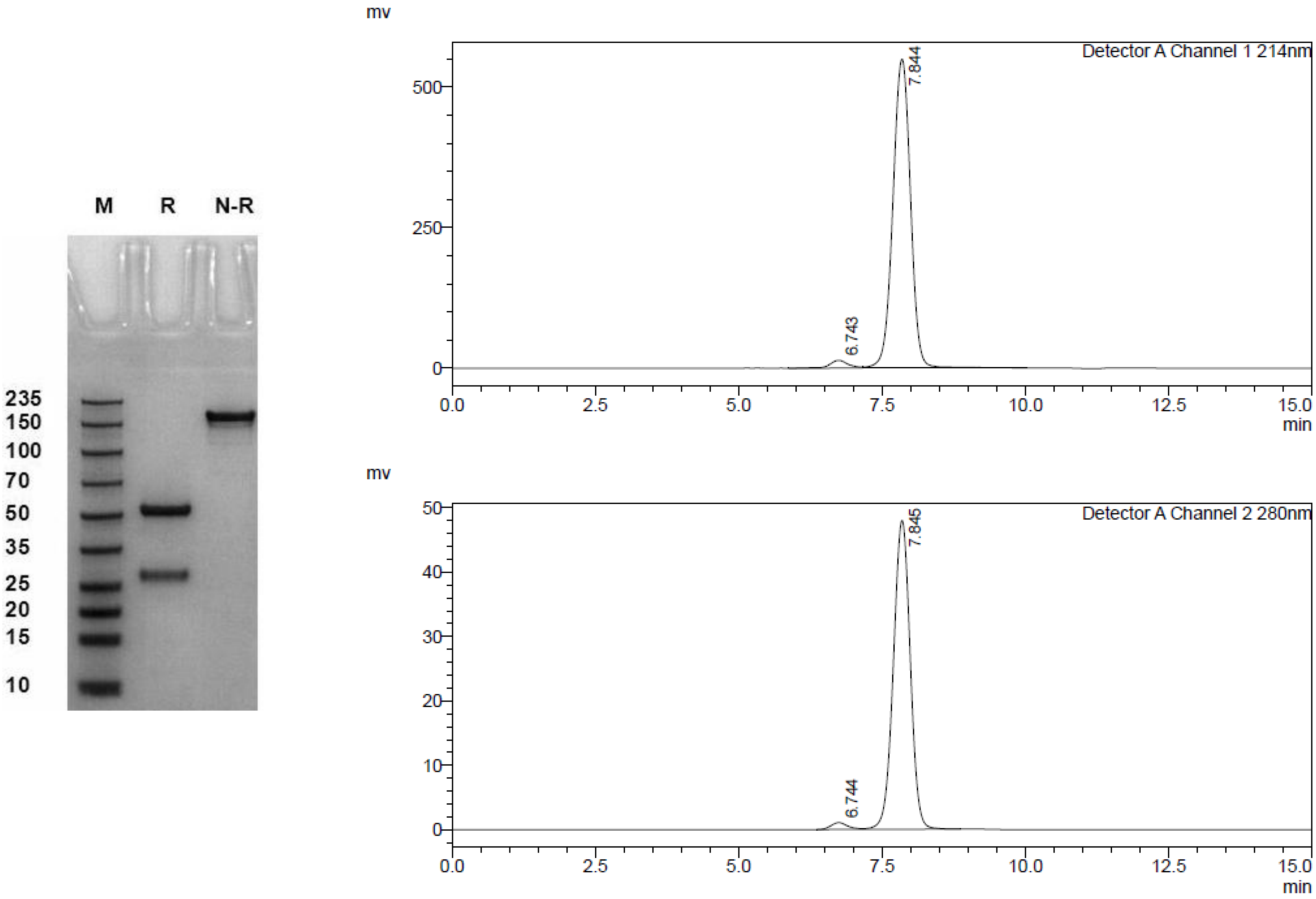

KBA2401_EVO102

Figure 3

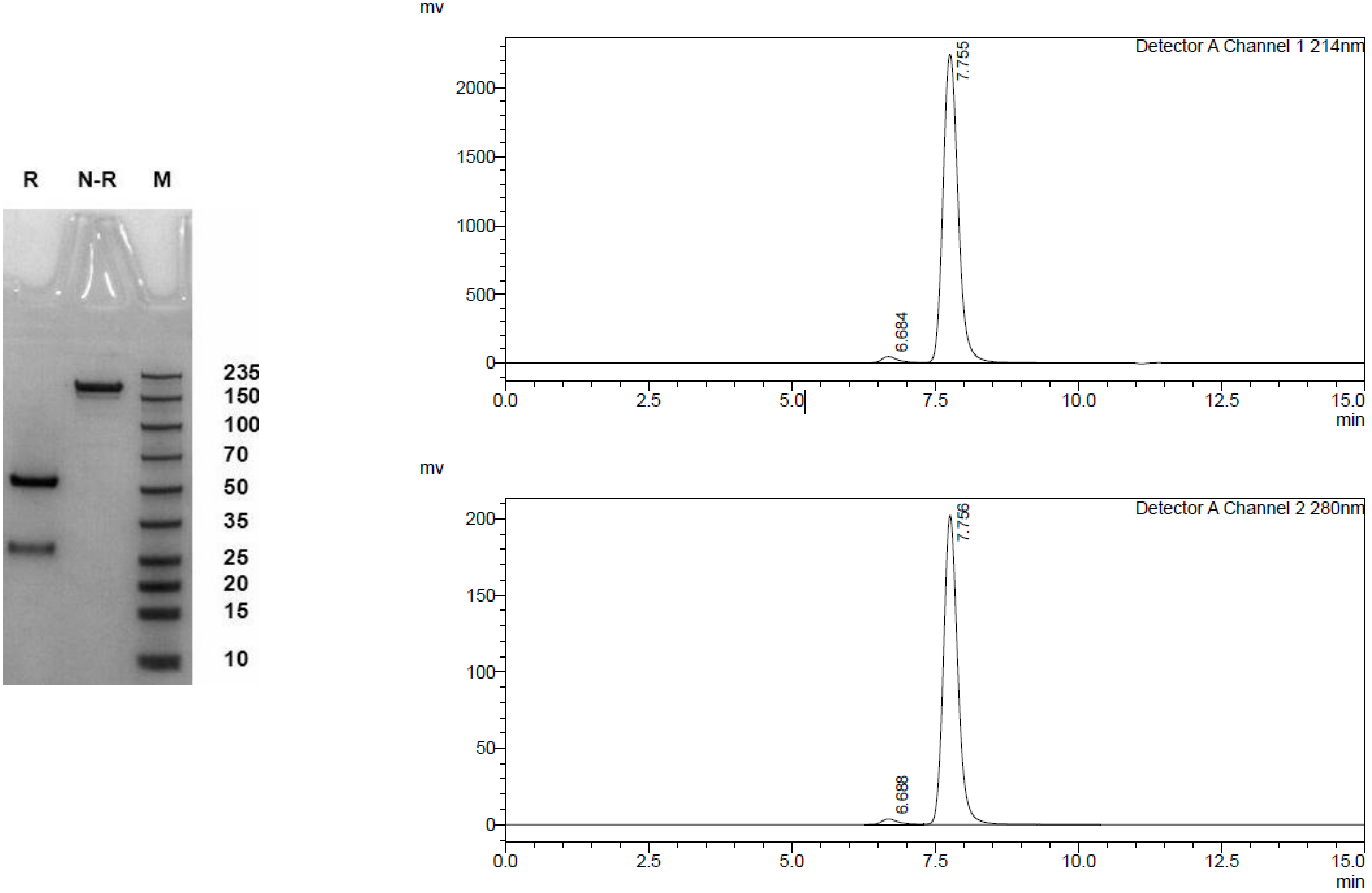

KBA2401_EVO107

Figure 3

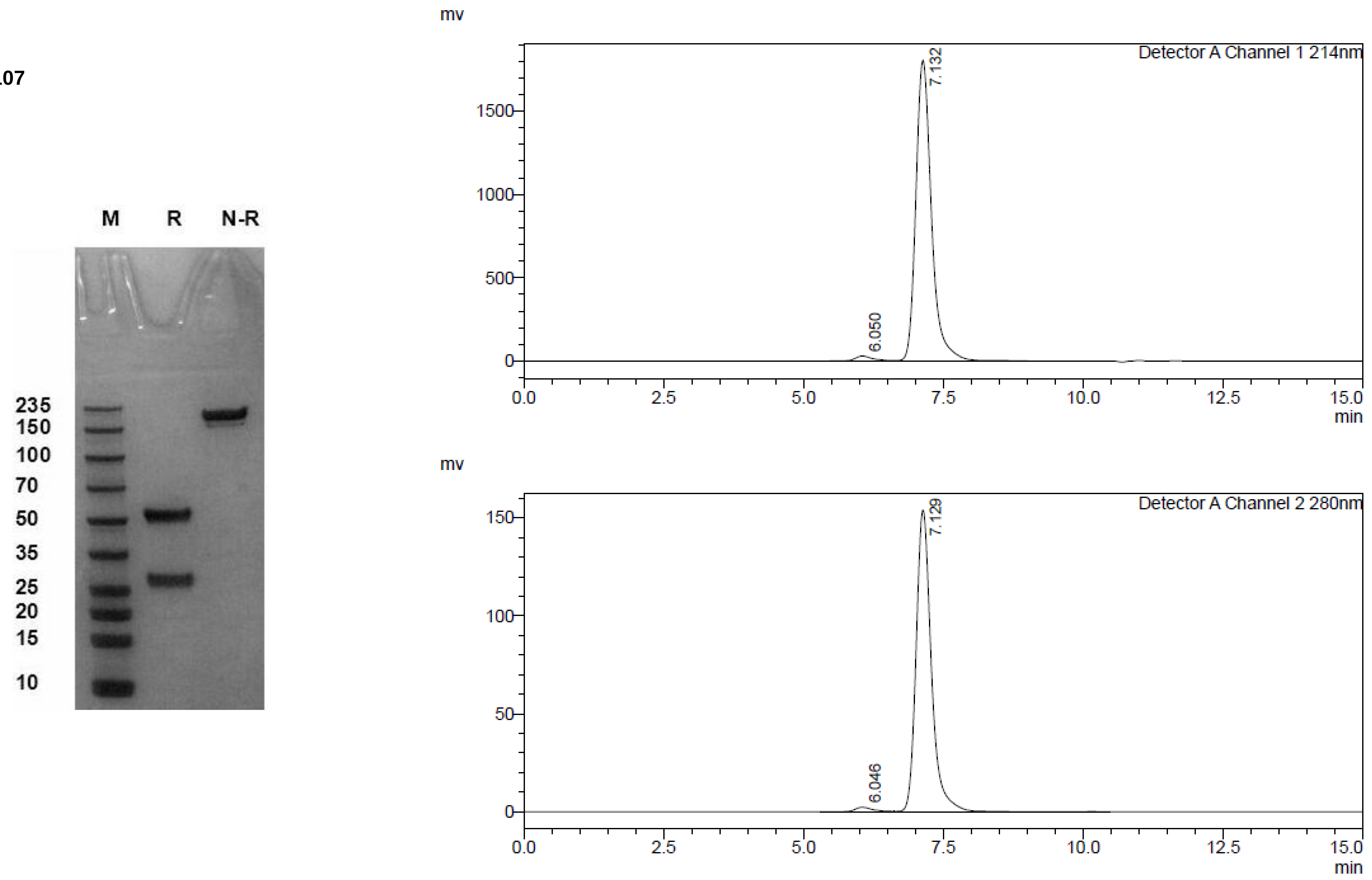

KBA2401_EVO108
Figure 3

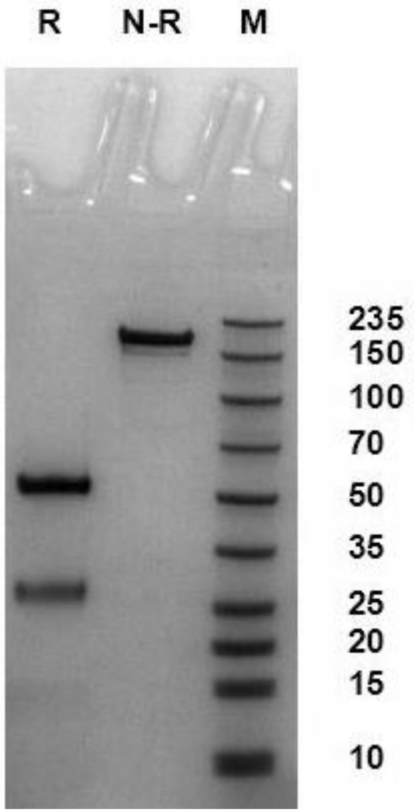

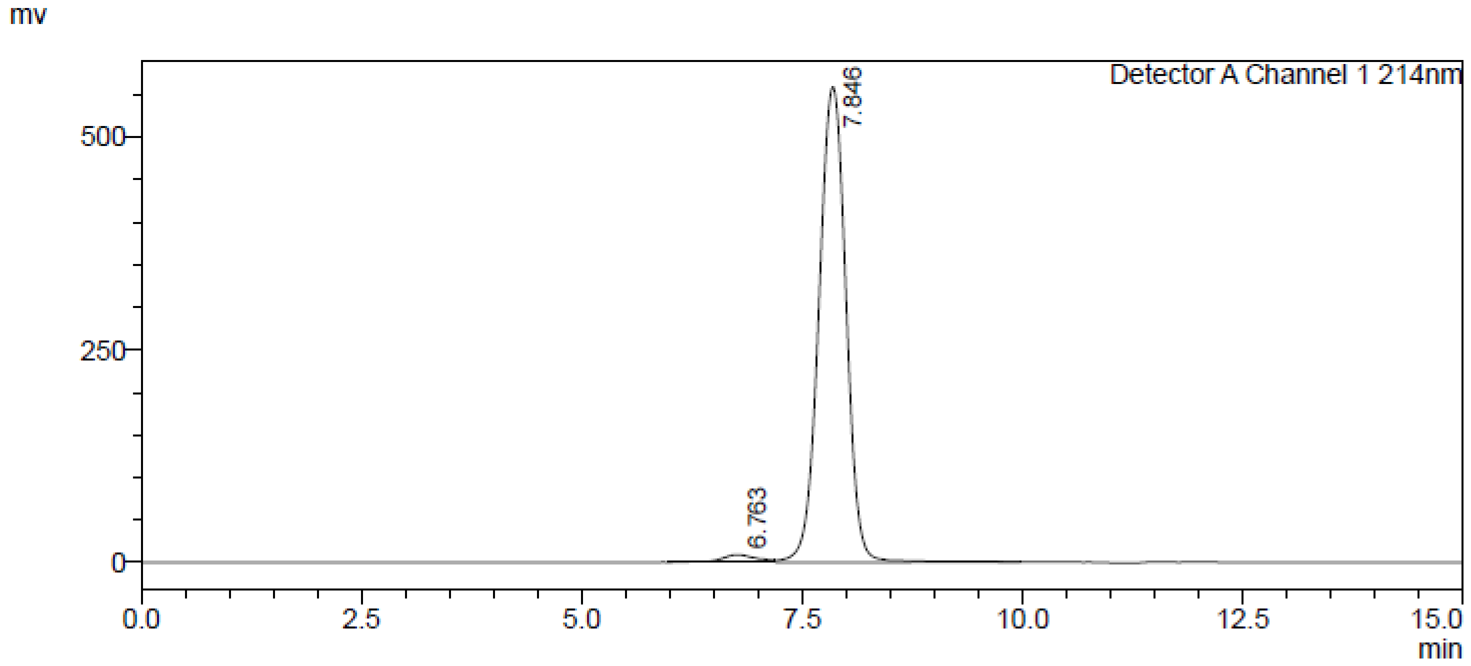

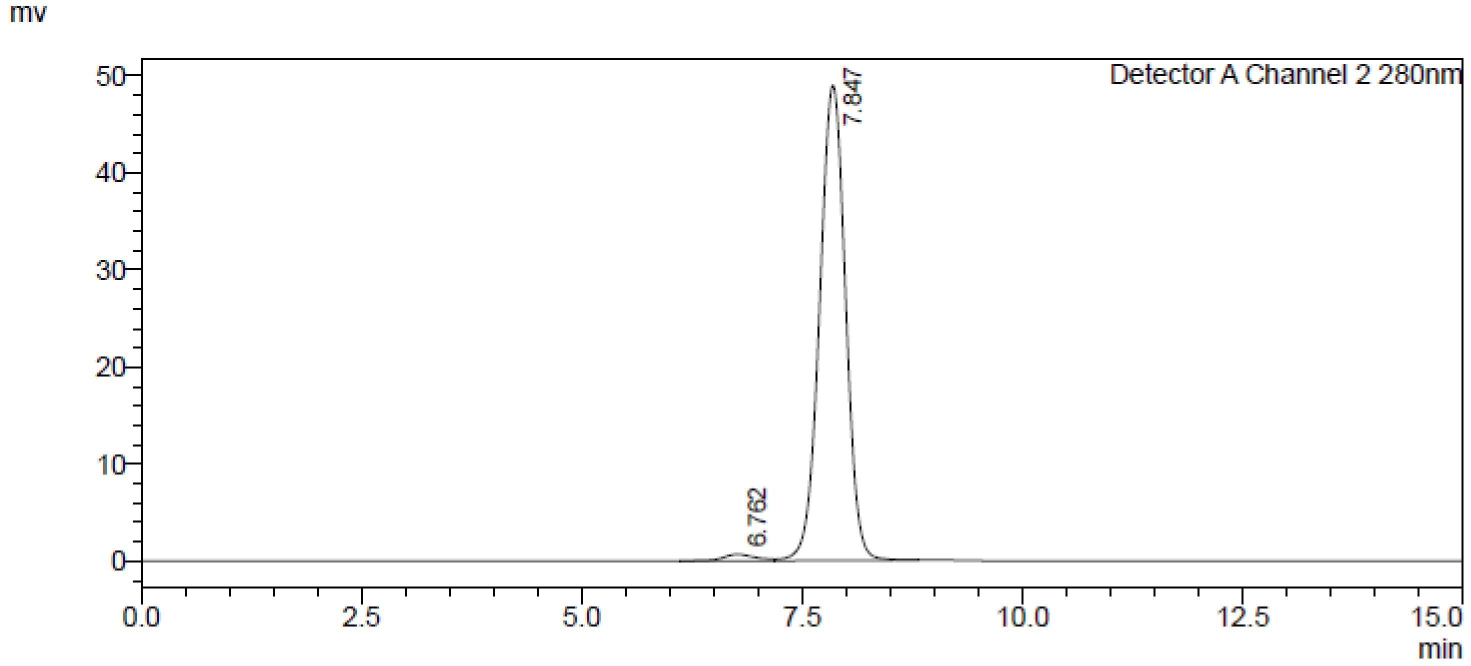

KBA2401_EVO109

Figure 3

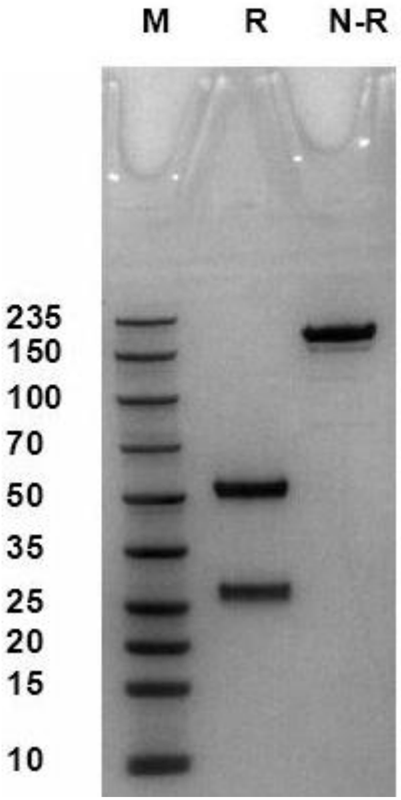

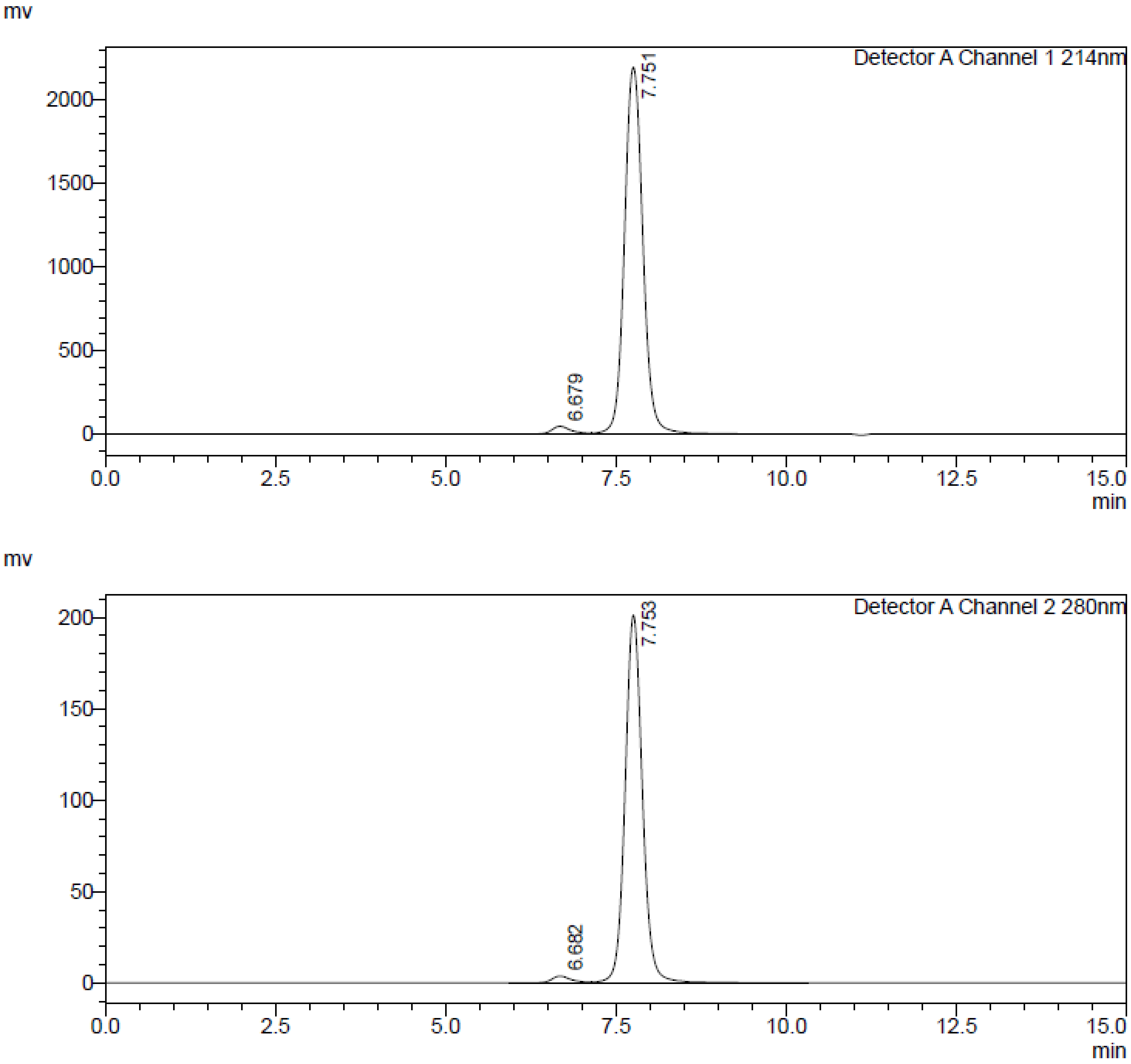

KBA2401_EVO112

Figure 3

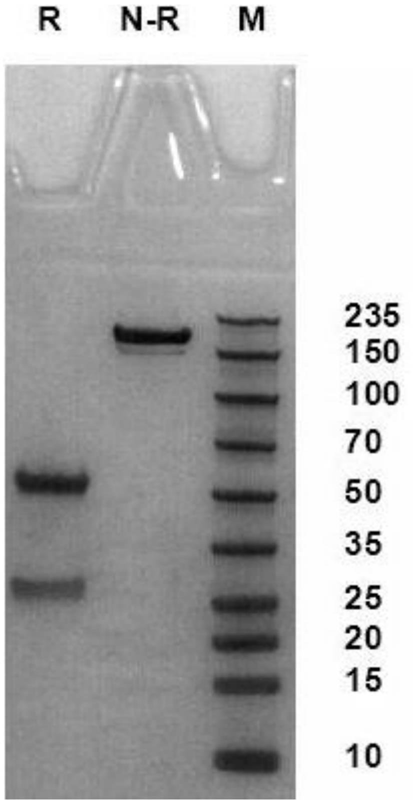

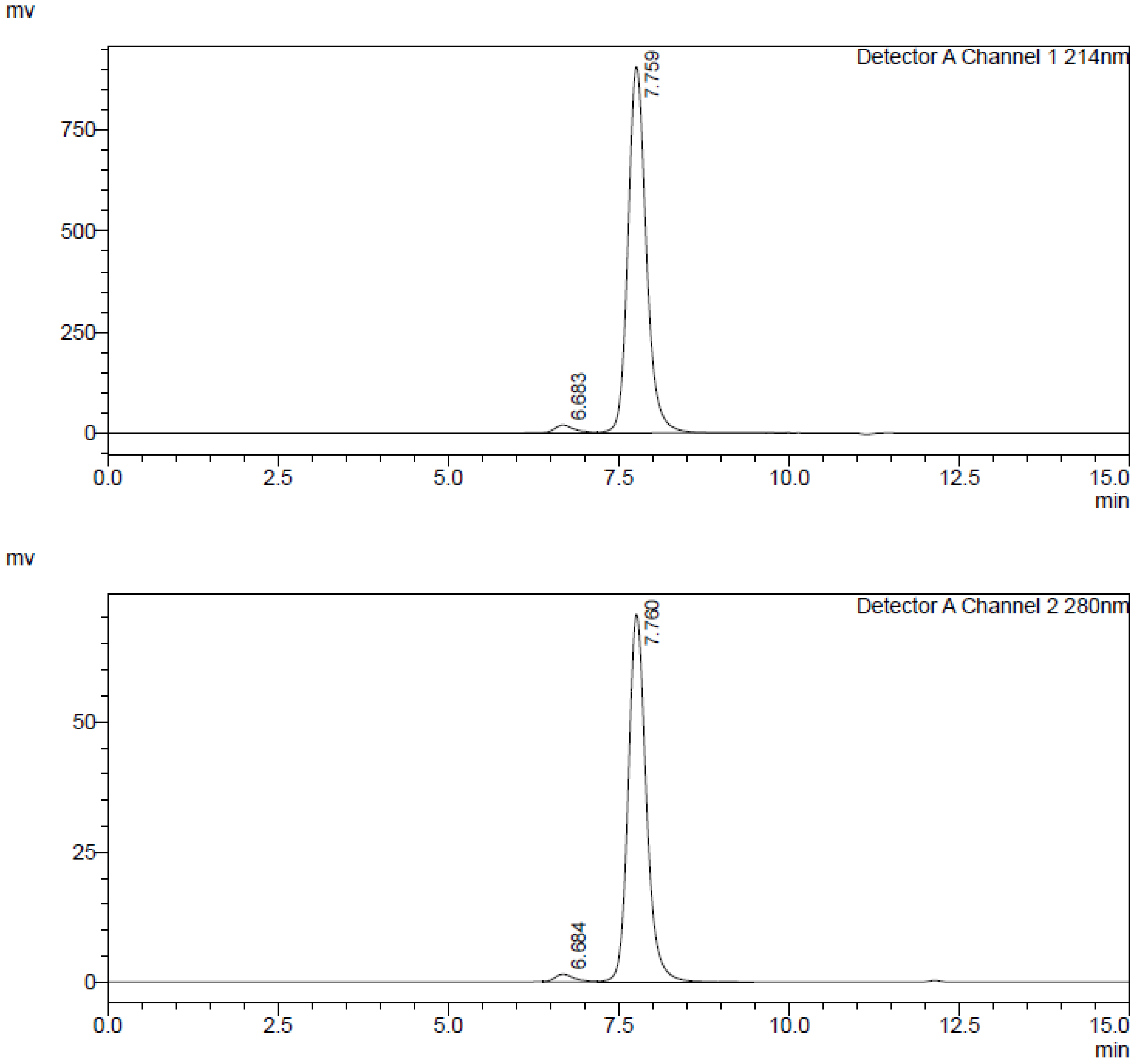

KBA2401_EVO114

Figure 3

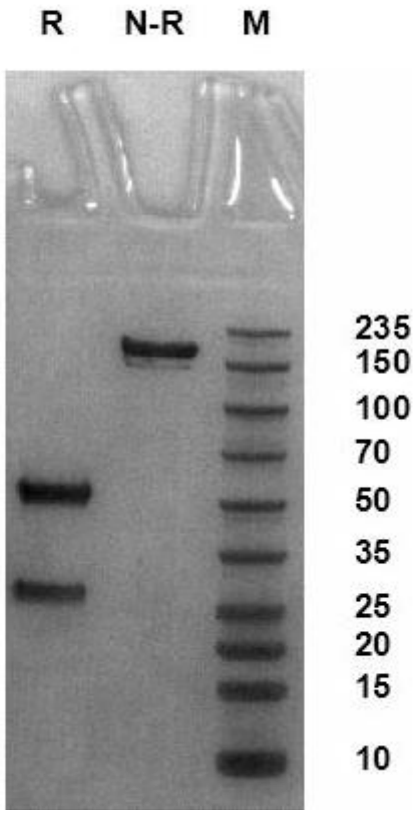

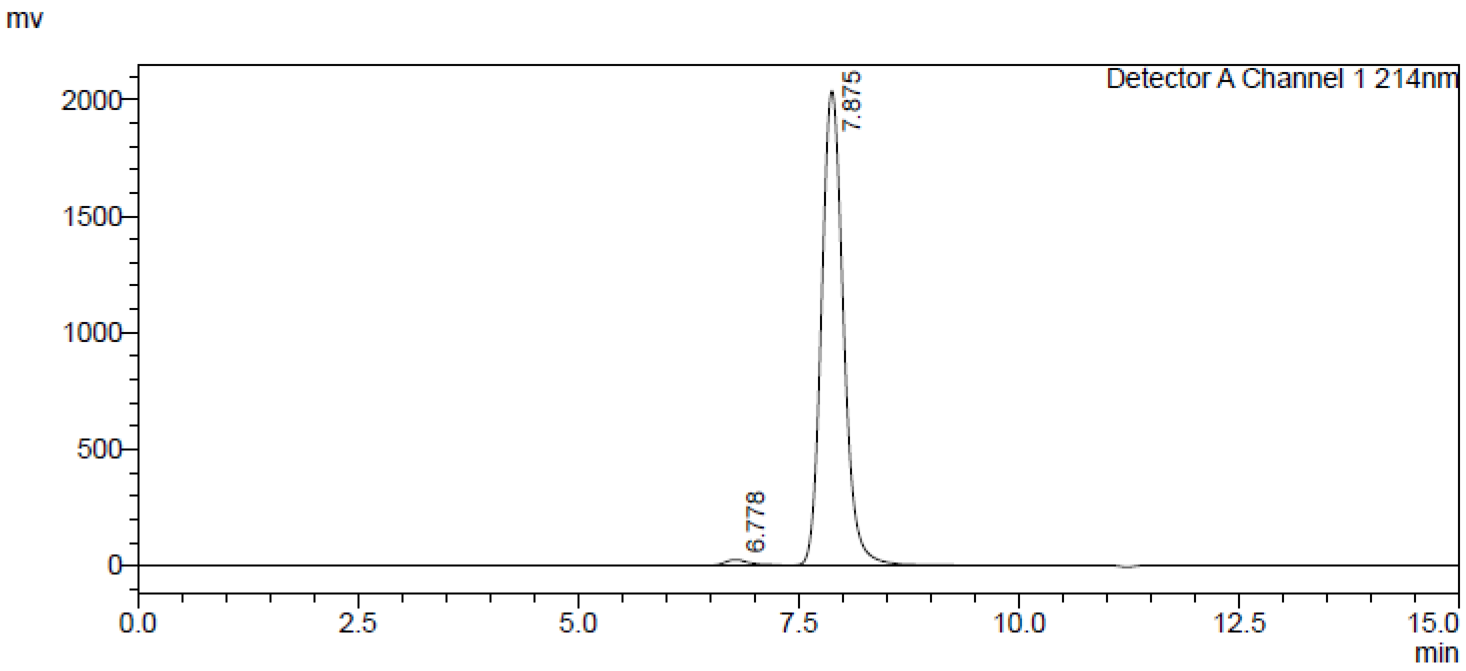

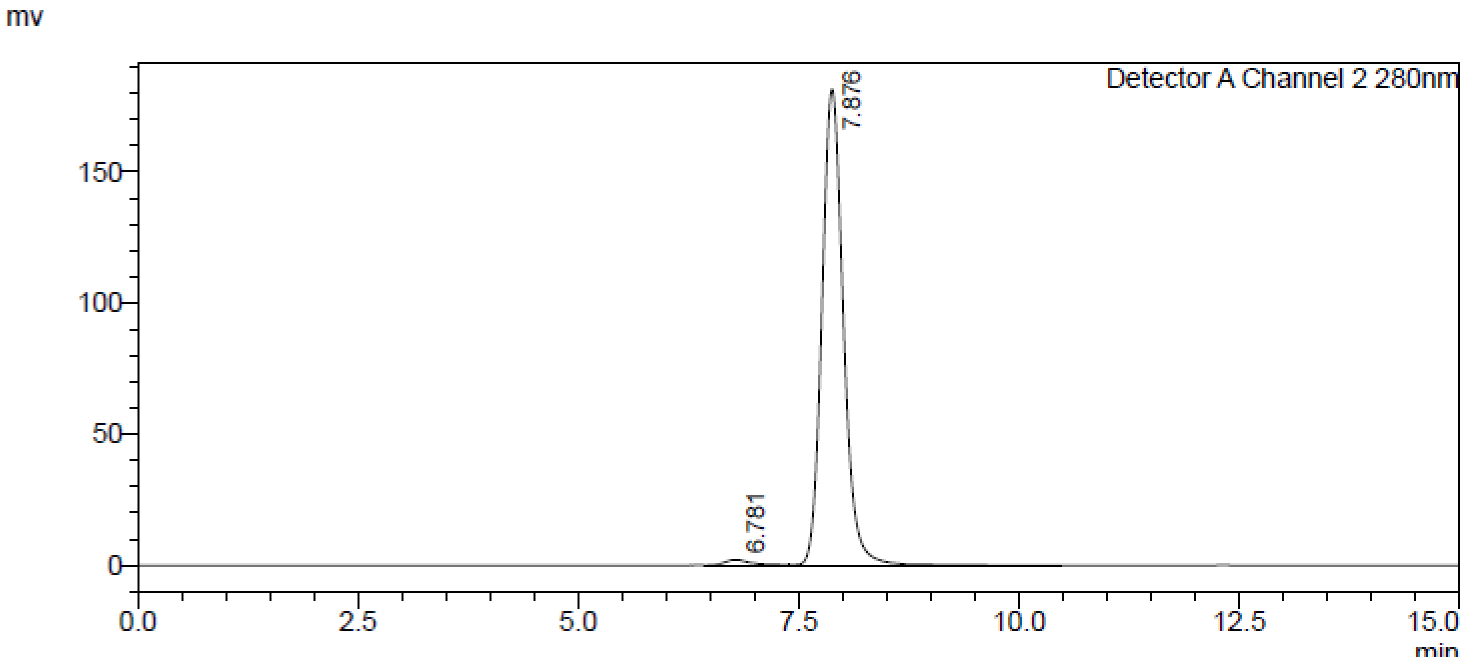

KBA2401_EVO115

Figure 3

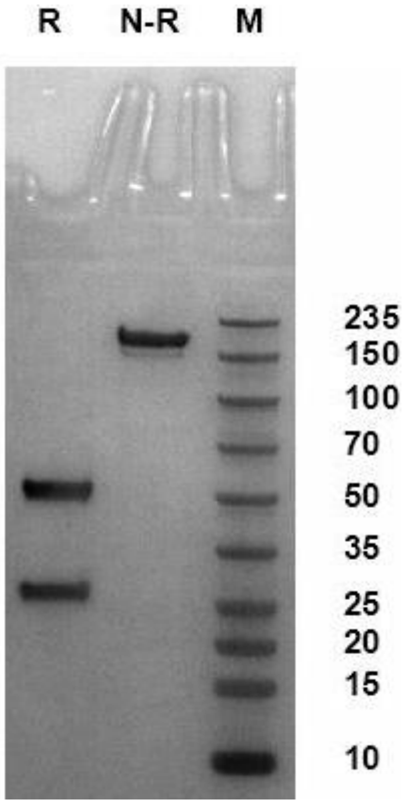

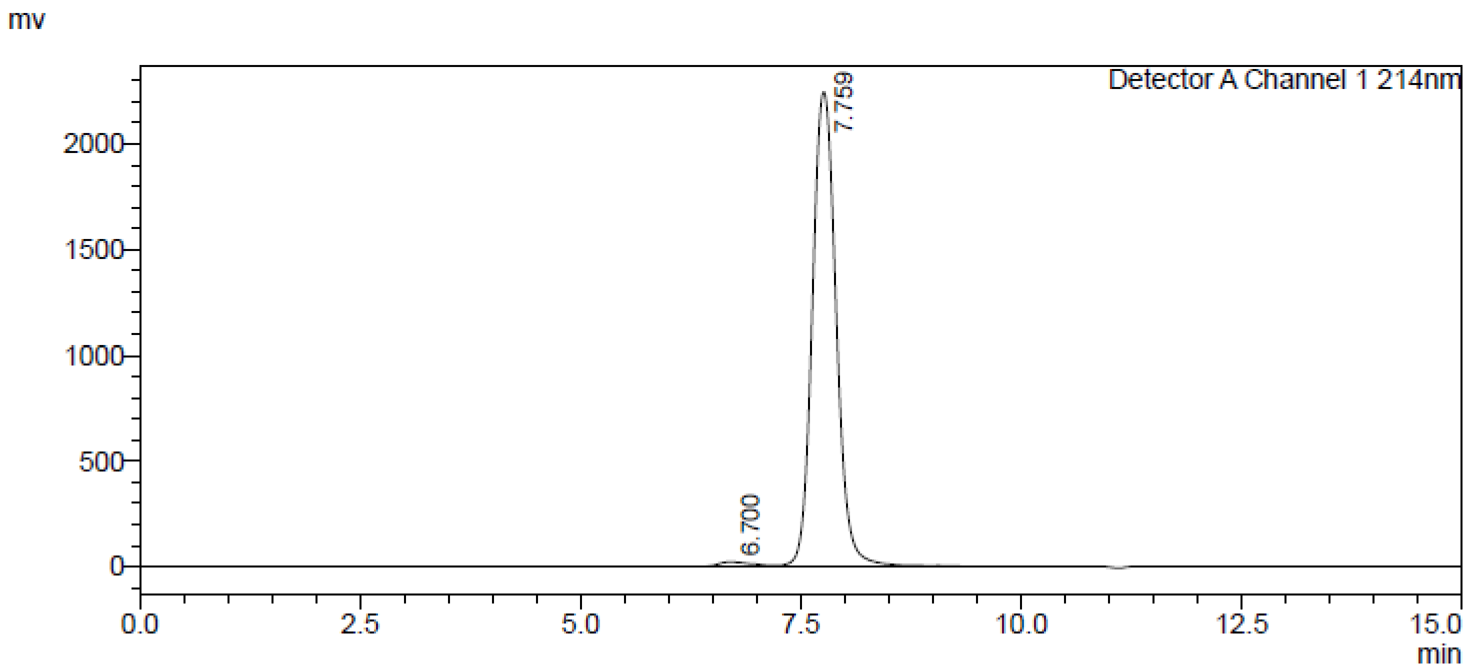

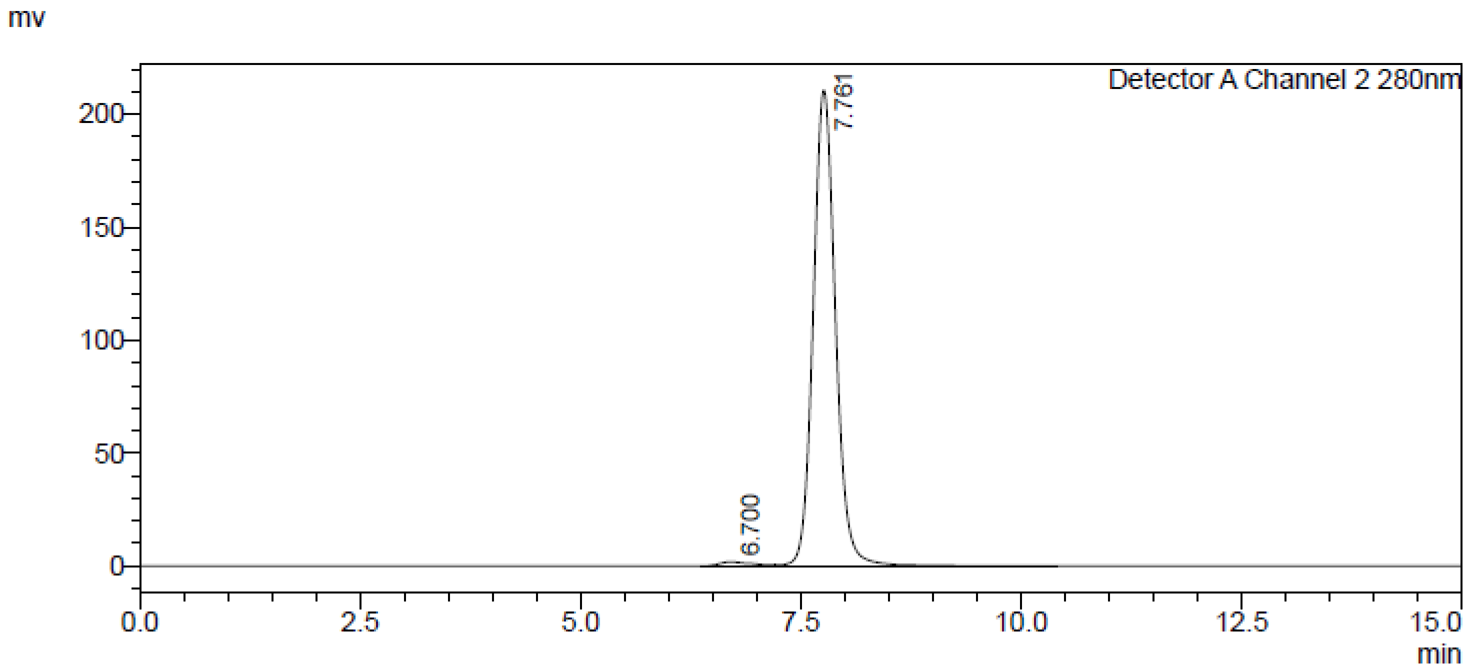

KBA2401_EVO121

Figure 3

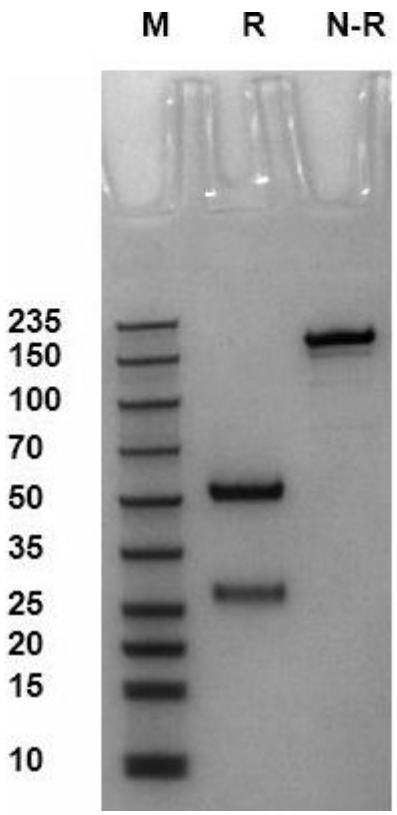

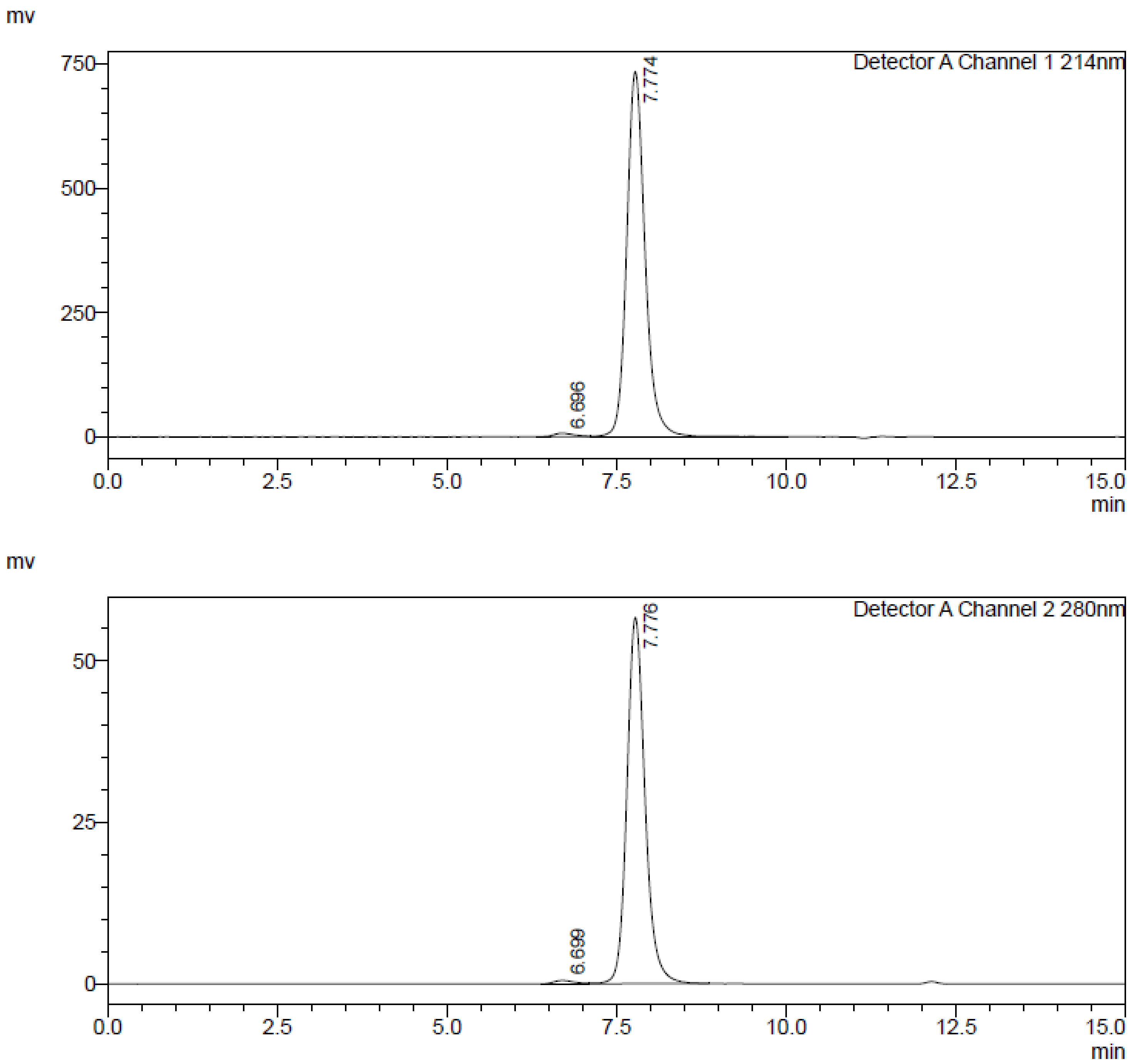

KBA2401_EVO122

Figure 3

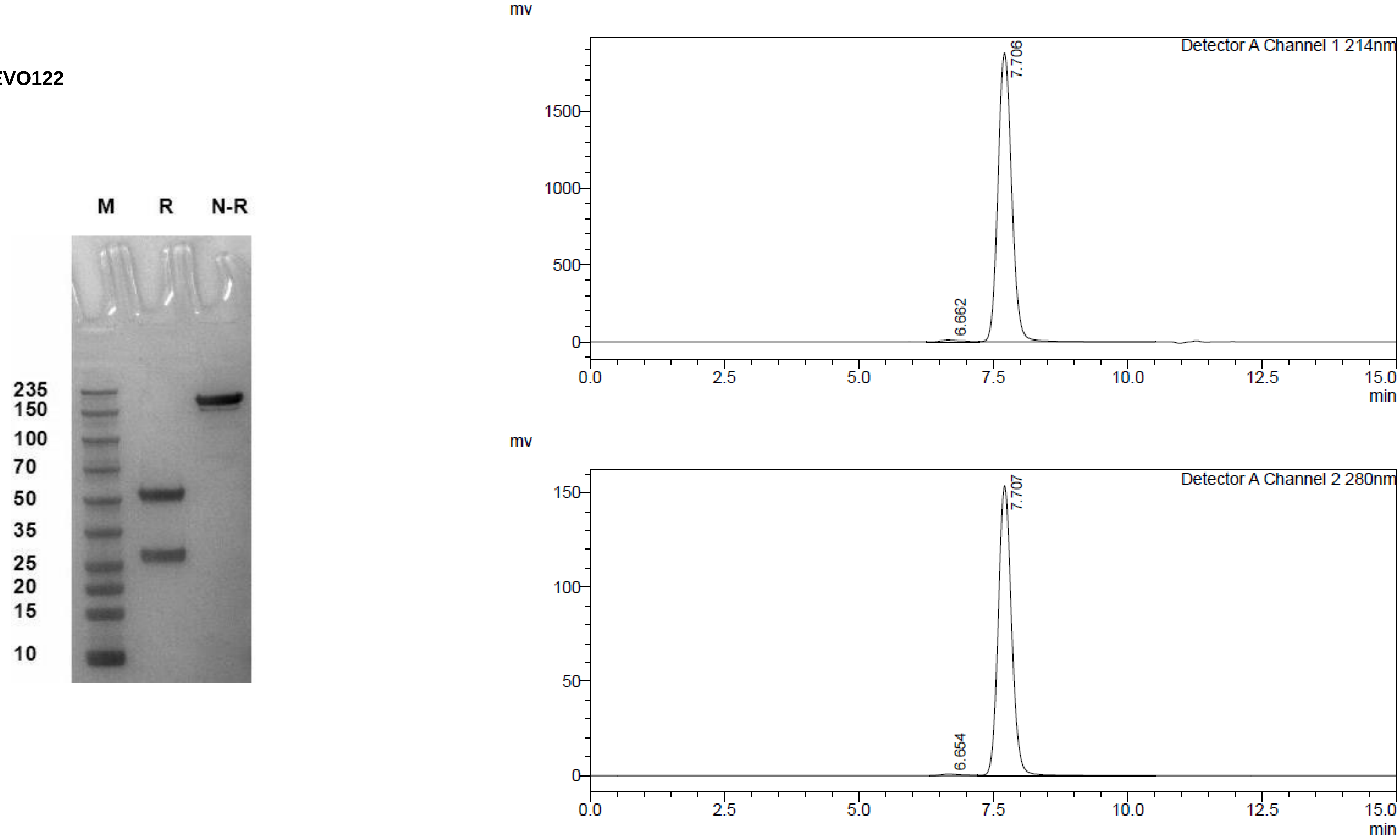

KBA2401_EVO124

Figure 3

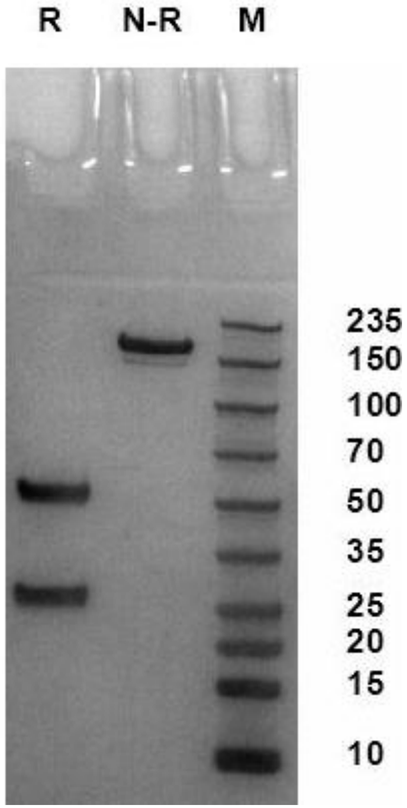

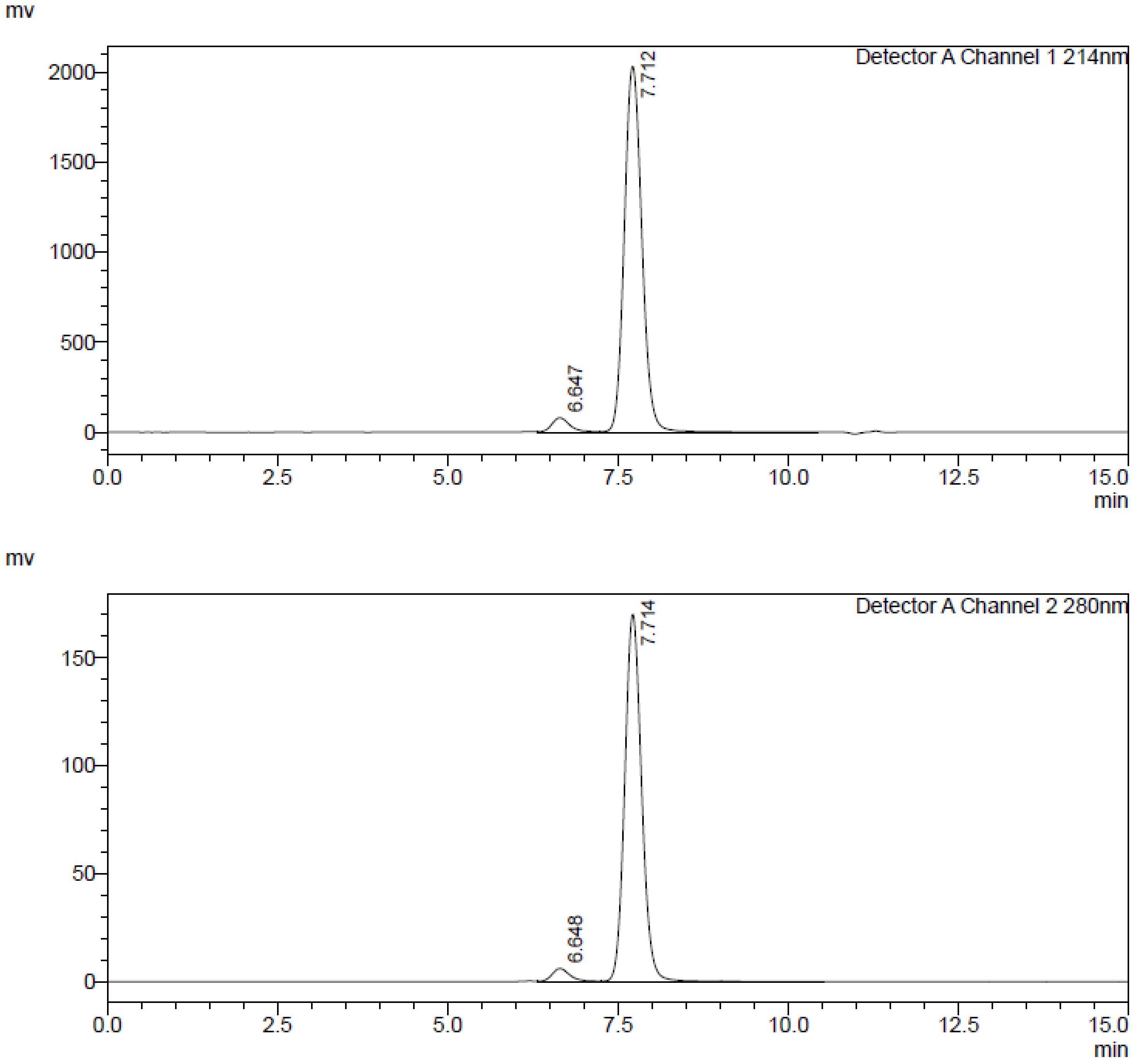

KBA2401_EVO136

Figure 3

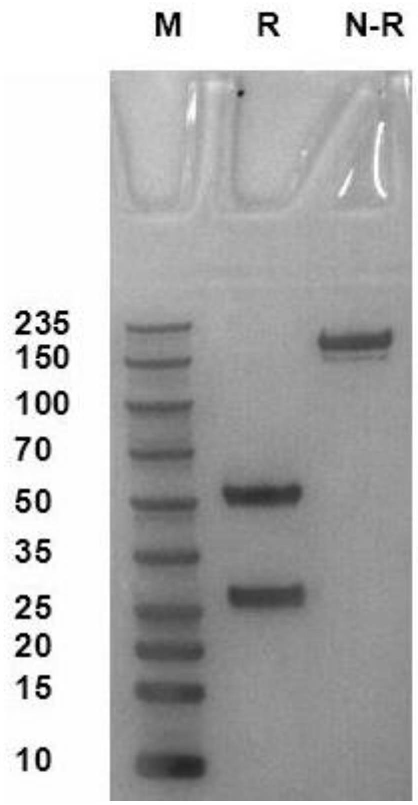

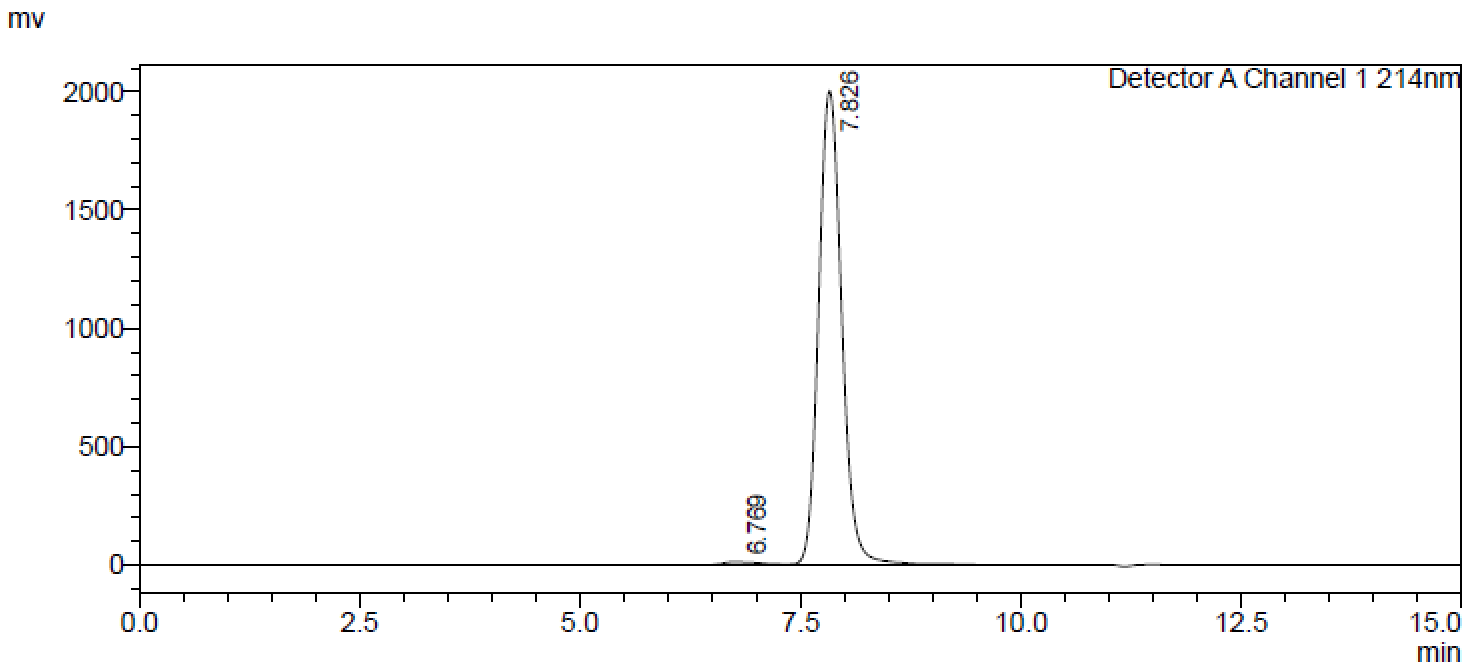

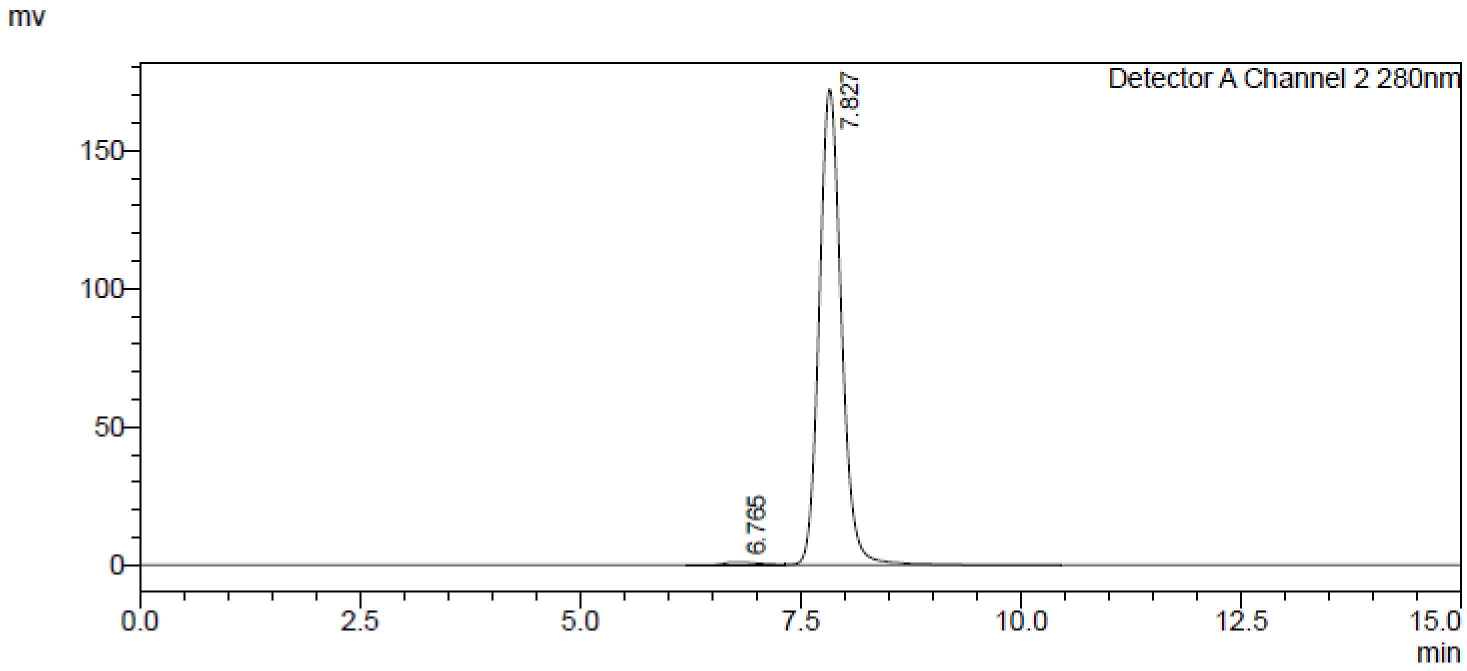

KBA2401_EVO144

Figure 3

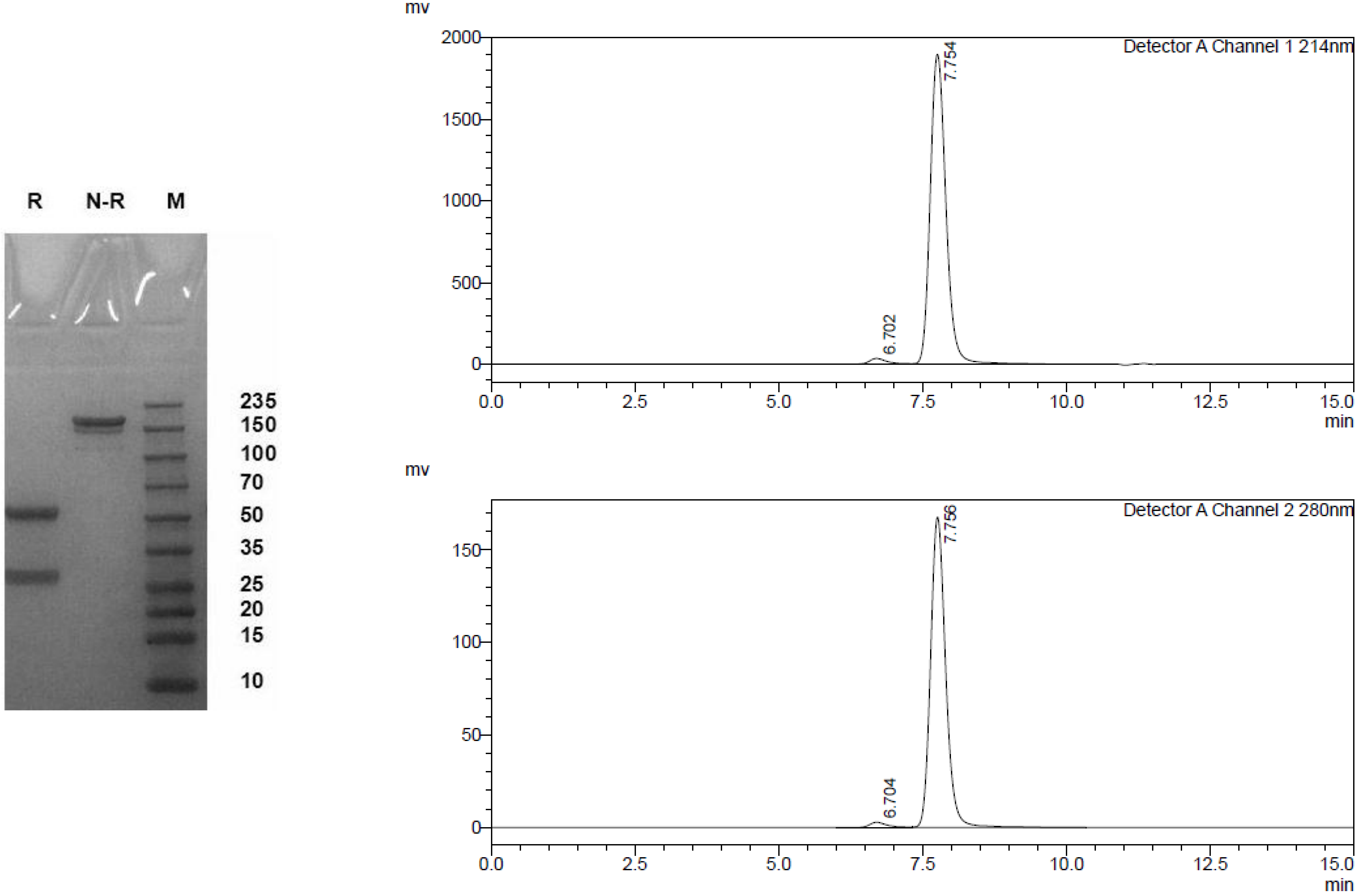

KBA2402 and EVO supernatants, 1 ml CHO supernatant production
Figure 3

Kling Paper name	Concentration (mg/mL)
KBA2402_EVO.001	0.470
KBA2402_EVO.002	0.351
KBA2402_EVO.003	0.234
KBA2402_EVO.004	0.474
KBA2402_EVO.005	0.345
KBA2402_EVO.006	0.384
KBA2402_EVO.007	0.391
KBA2402_EVO.008	0.317
KBA2402_EVO.009	0.416
KBA2402_EVO.010	0.239
KBA2402_EVO.011	0.504
KBA2402_EVO.012	0.493
KBA2402_EVO.013	0.408
KBA2402_EVO.014	0.430
KBA2402_EVO.015	0.432
KBA2402_EVO.016	0.254
KBA2402_EVO.017	0.525
KBA2402_EVO.018	0.517
KBA2402_EVO.019	0.508
KBA2402_EVO.020	0.397
KBA2402_EVO.021	0.466
KBA2402_EVO.022	0.458
KBA2402_EVO.023	0.366
KBA2402_EVO.024	0.446
KBA2402 OC	0.303

2401*OC_2402*EVO14

Figure 5

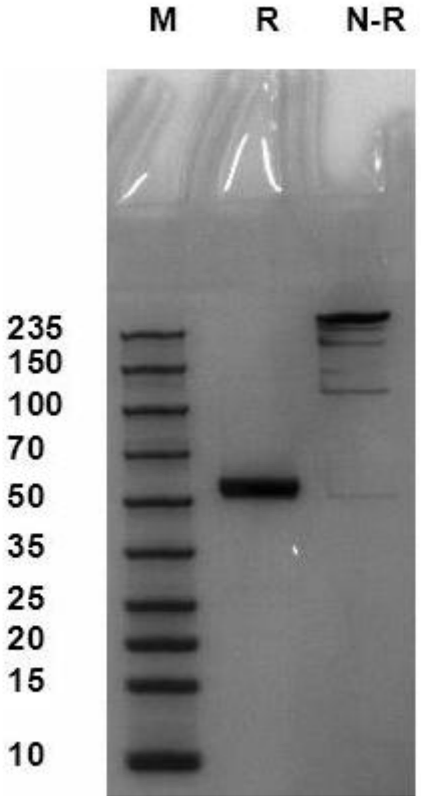

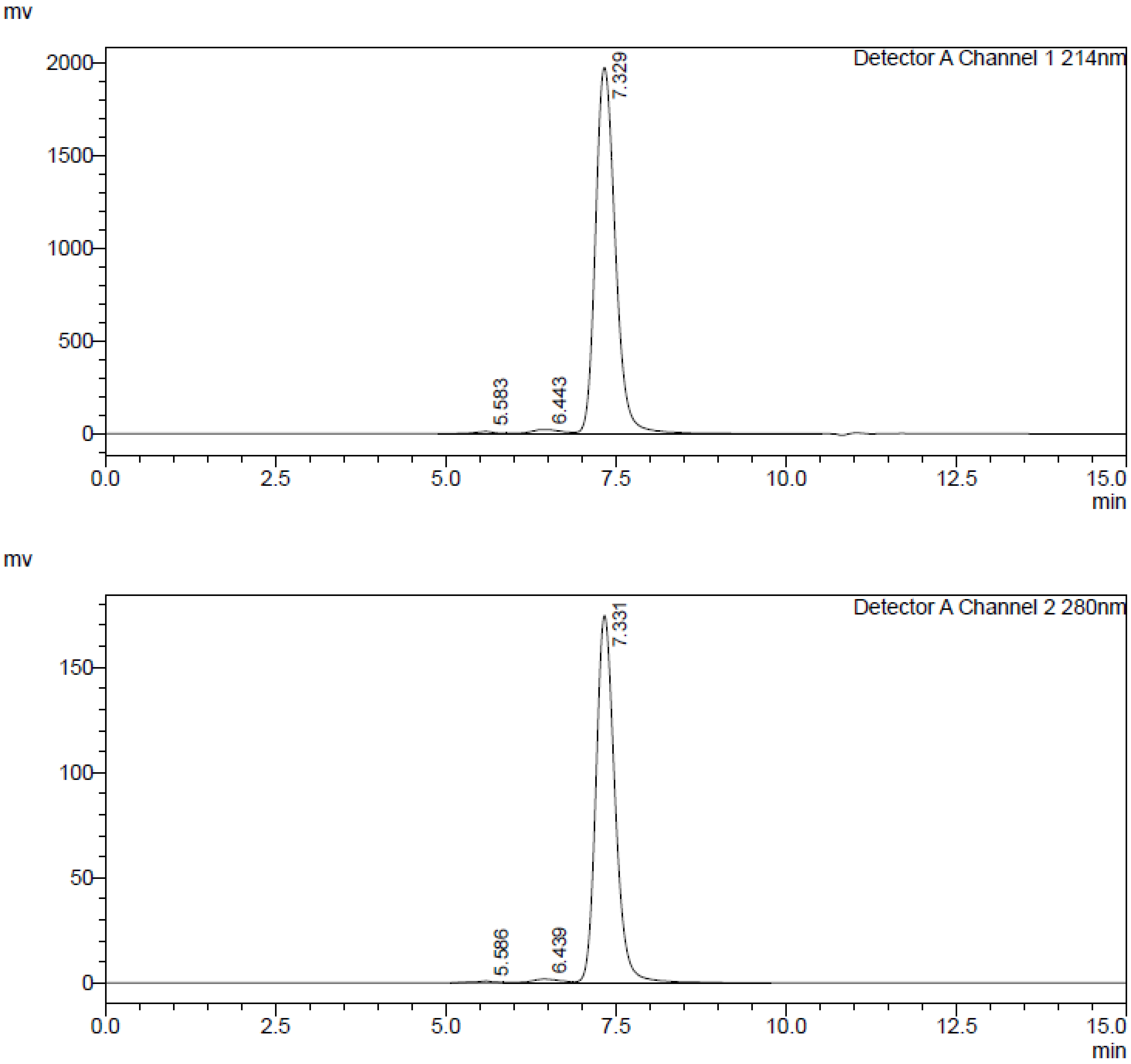

2401*EVO108_2402*EVO14

Figure 5

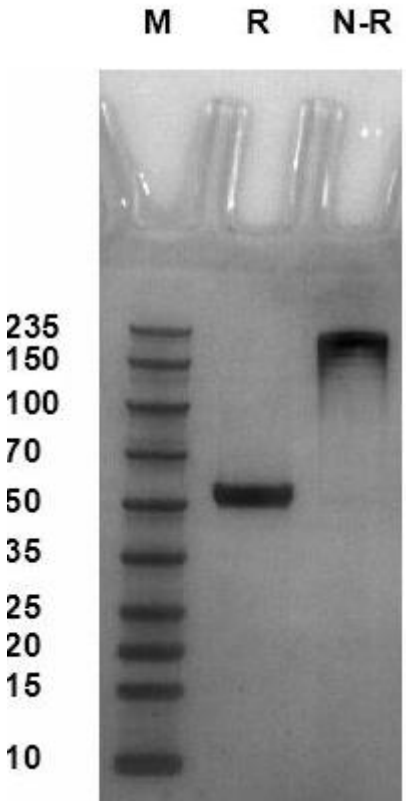

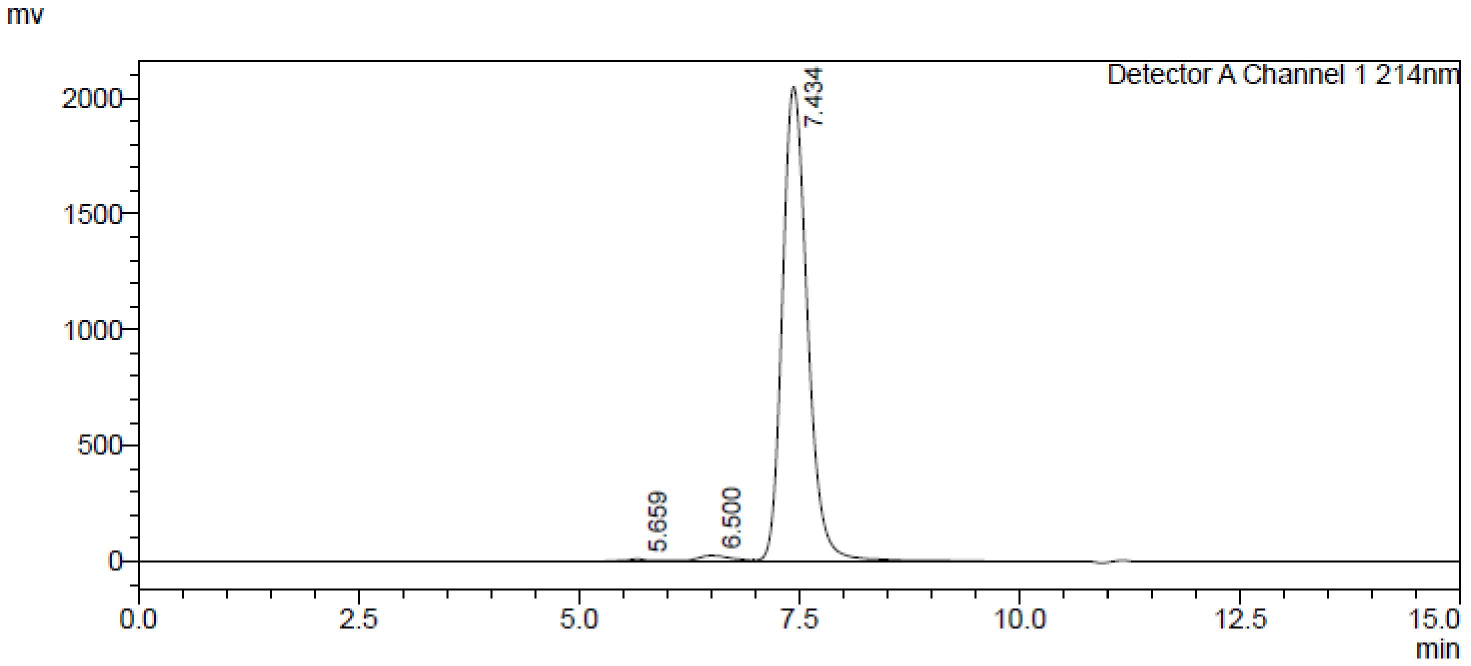

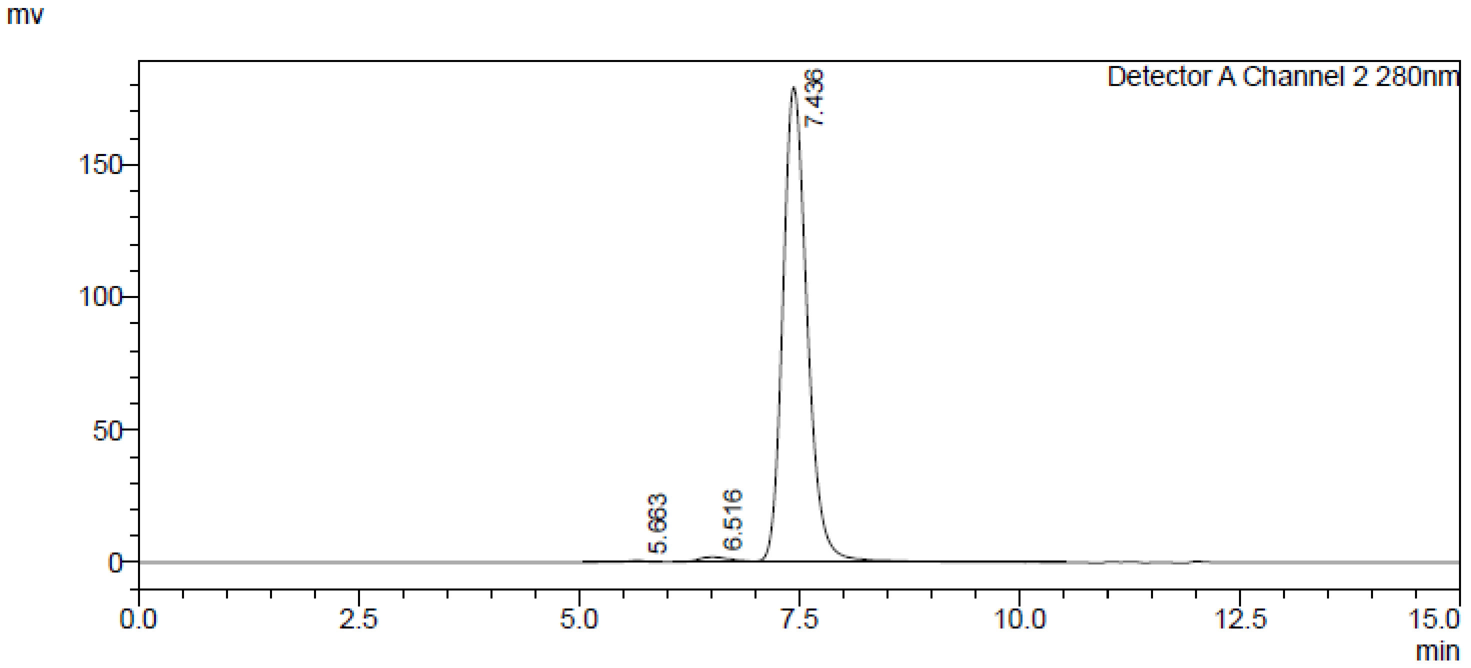

2401*EVO108_2402*EVO25

Figure 5

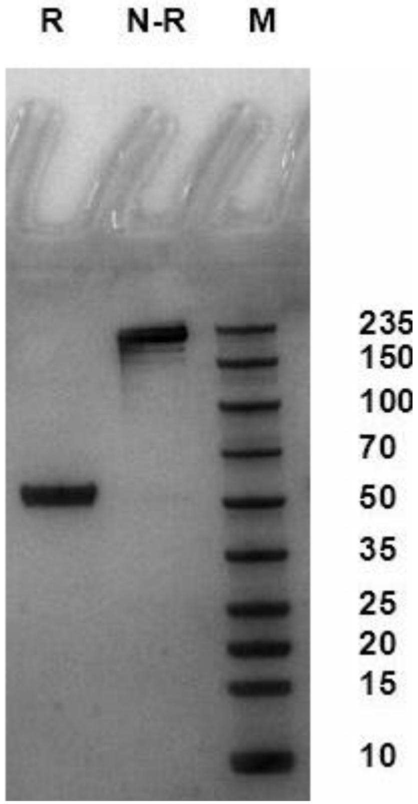

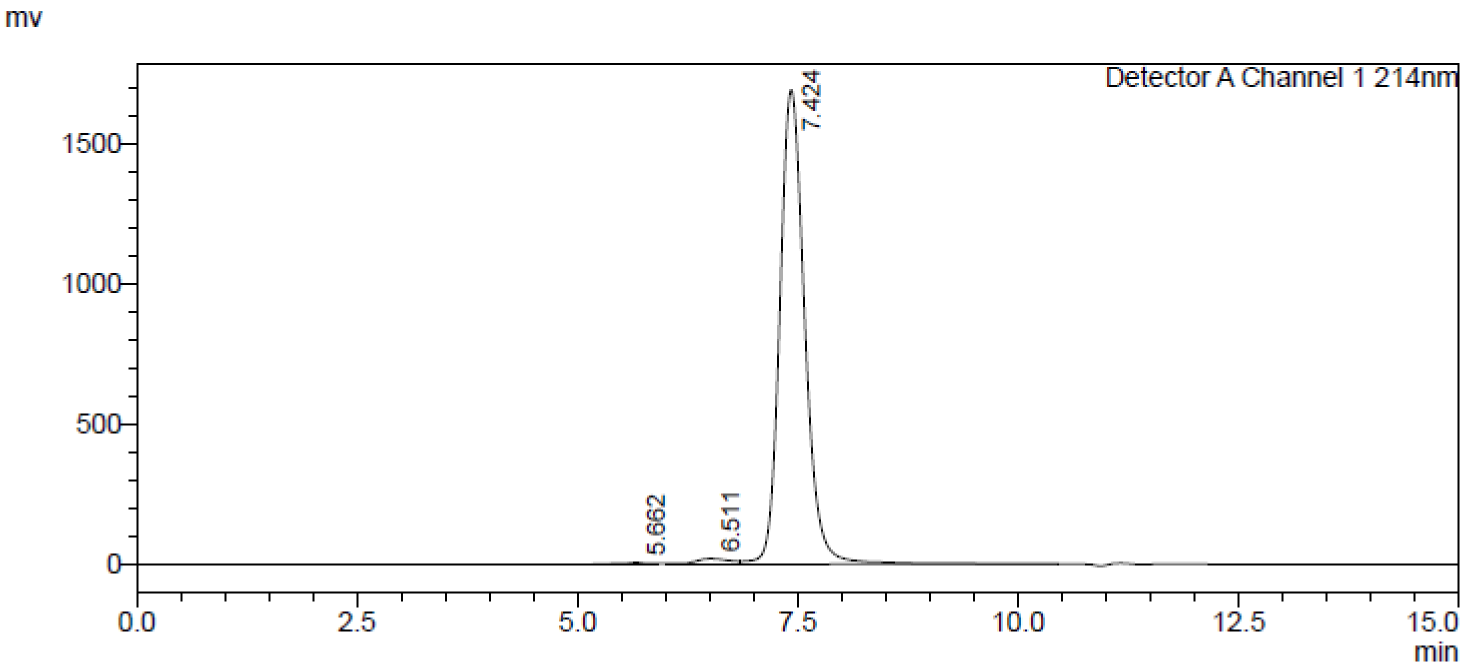

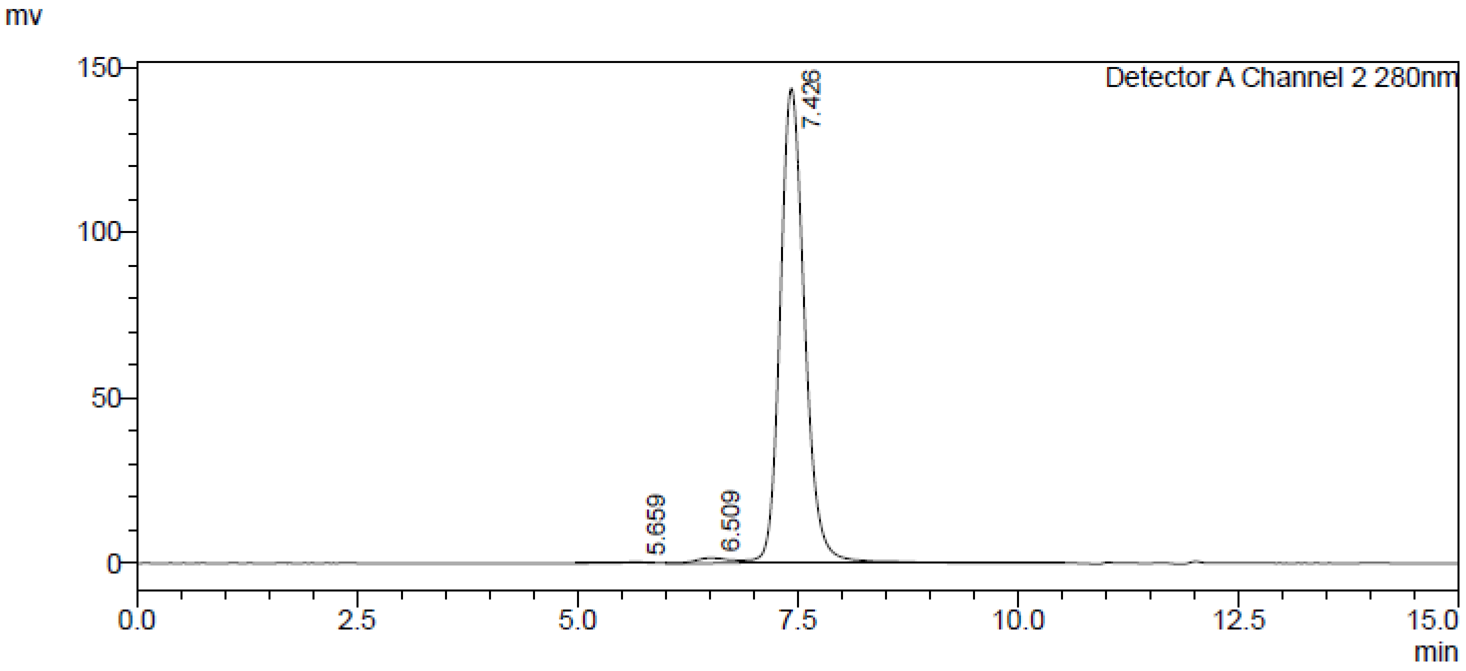

2401*EVO108_2402*EVO26

Figure 5

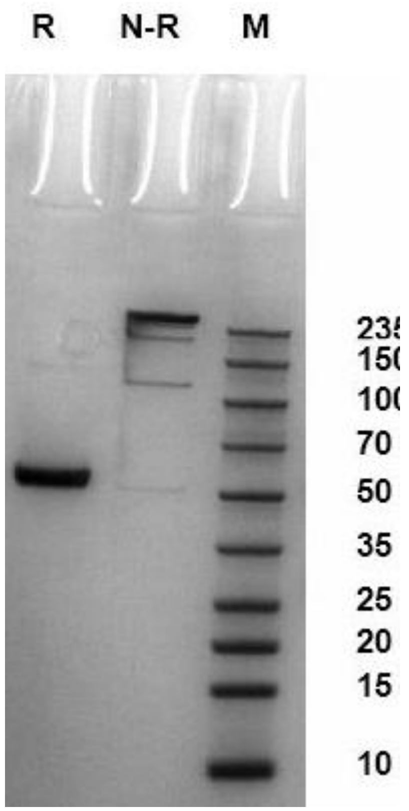

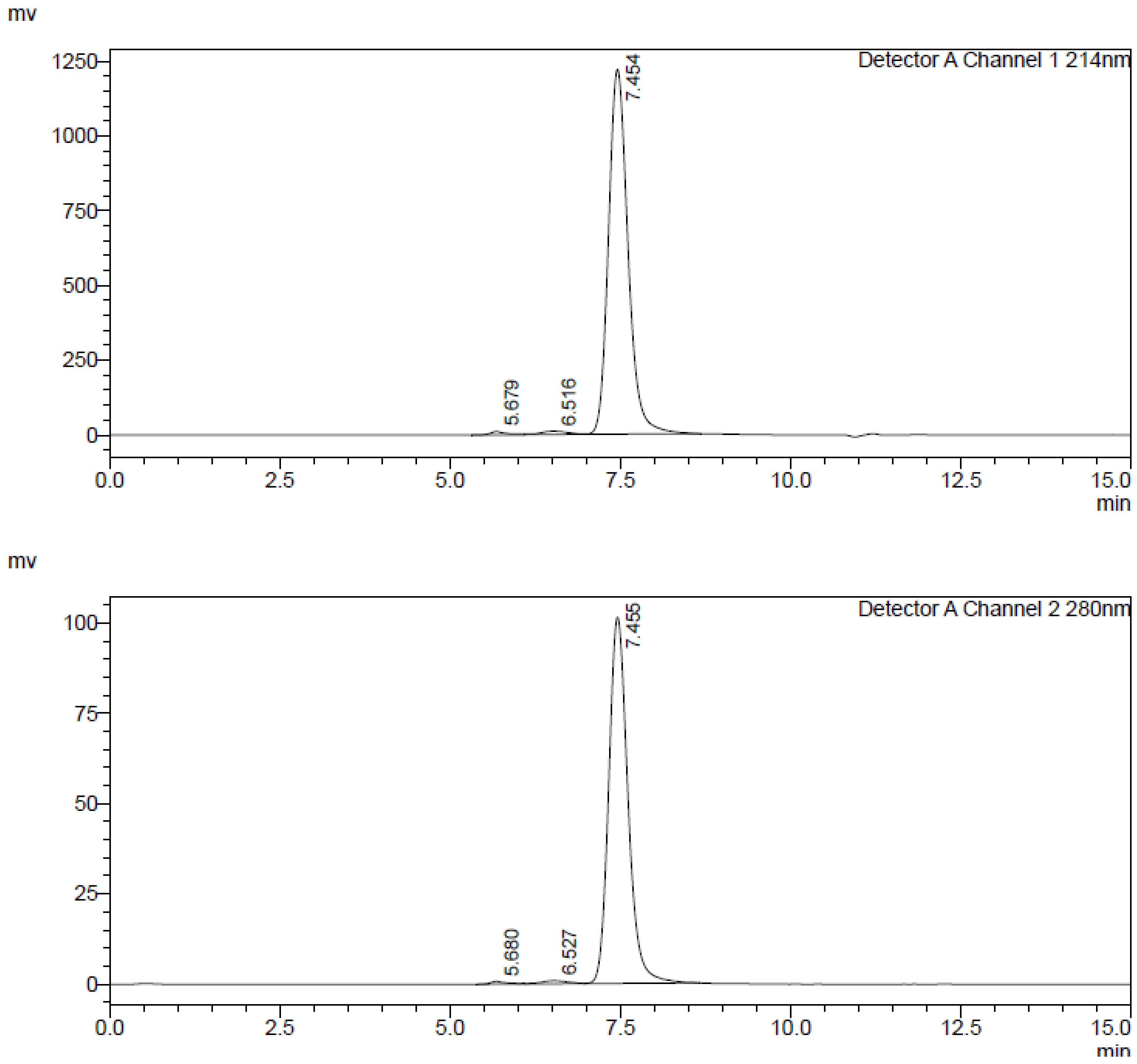

2401*EVO108_2402*EVO27

Figure 5

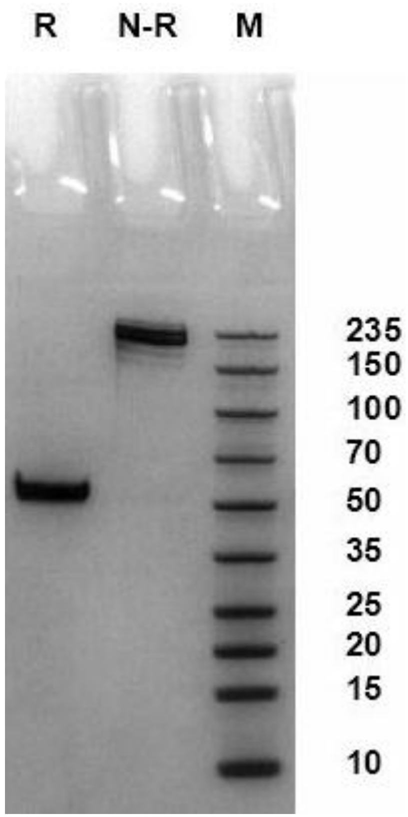

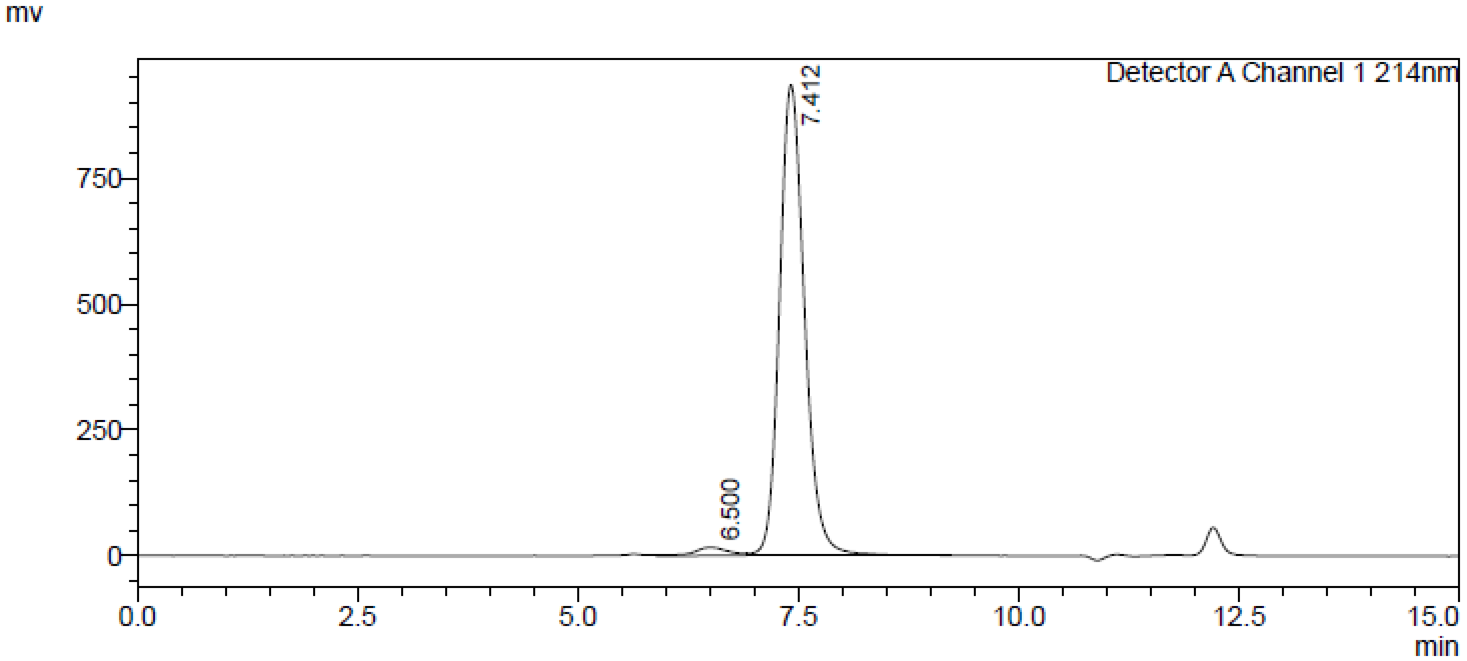

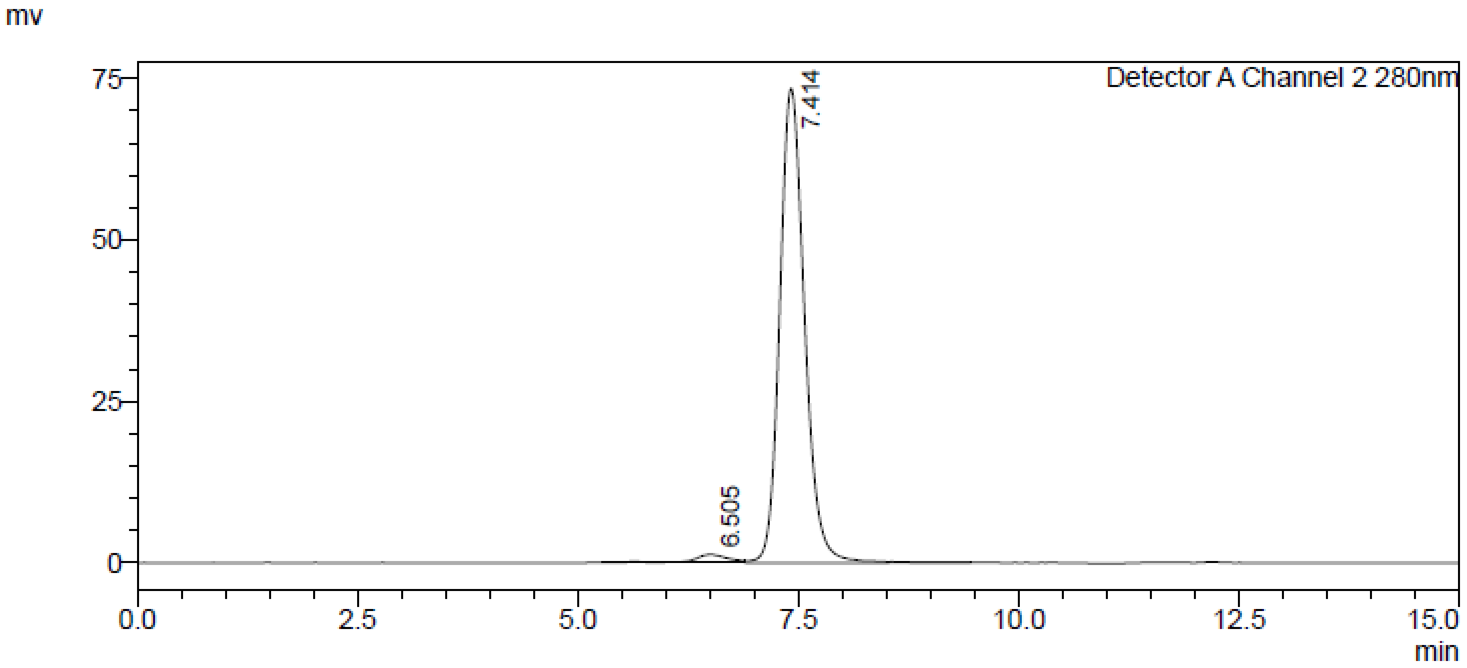

2401*EVO108_2402*EVO28

Figure 5

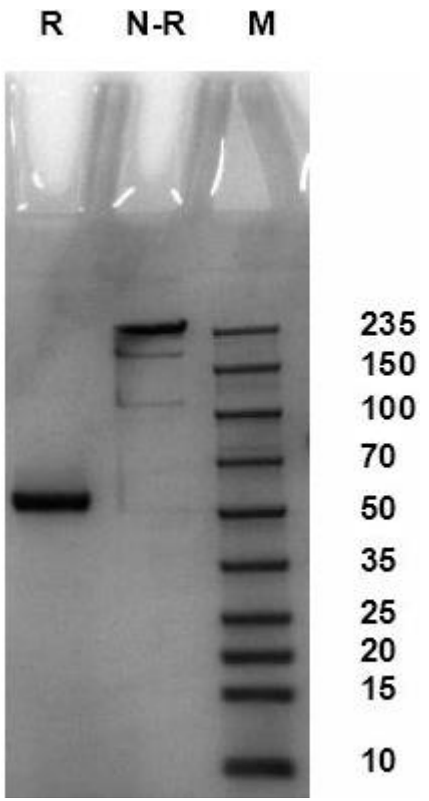

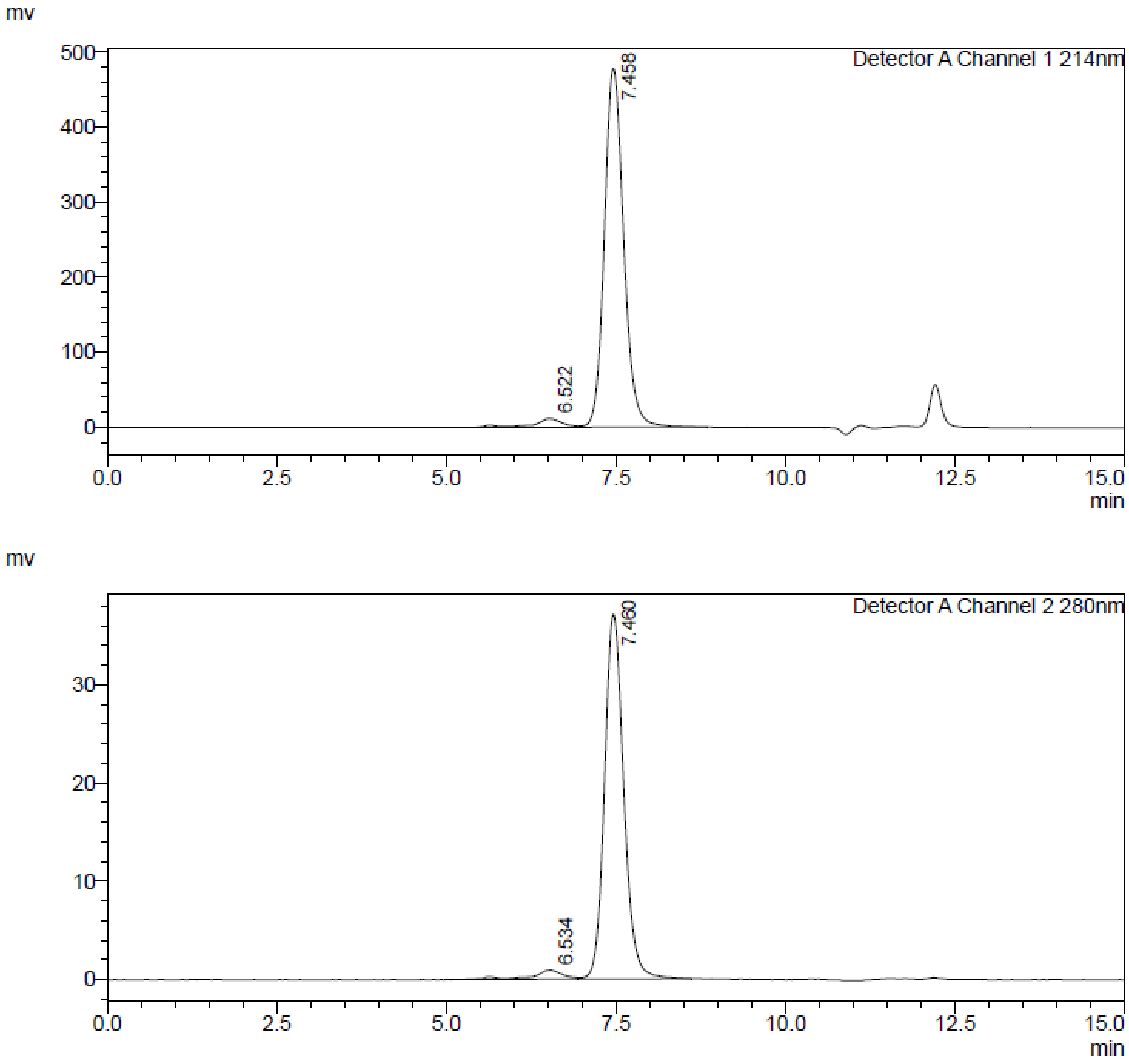

2401*EVO108_2402*EVO29

Figure 5

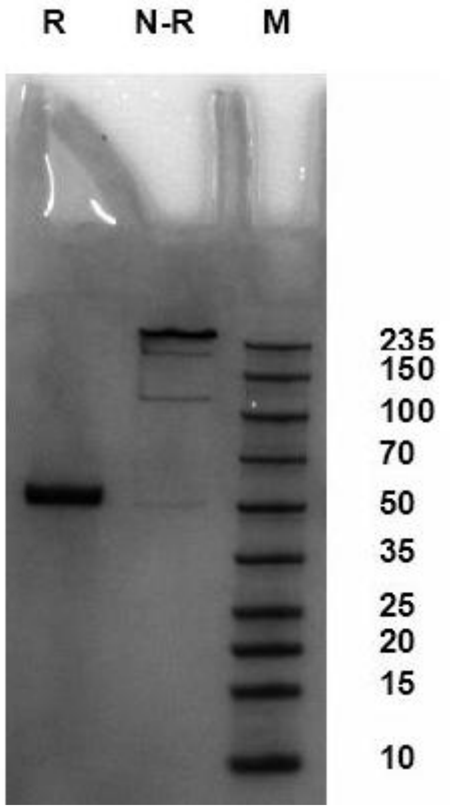

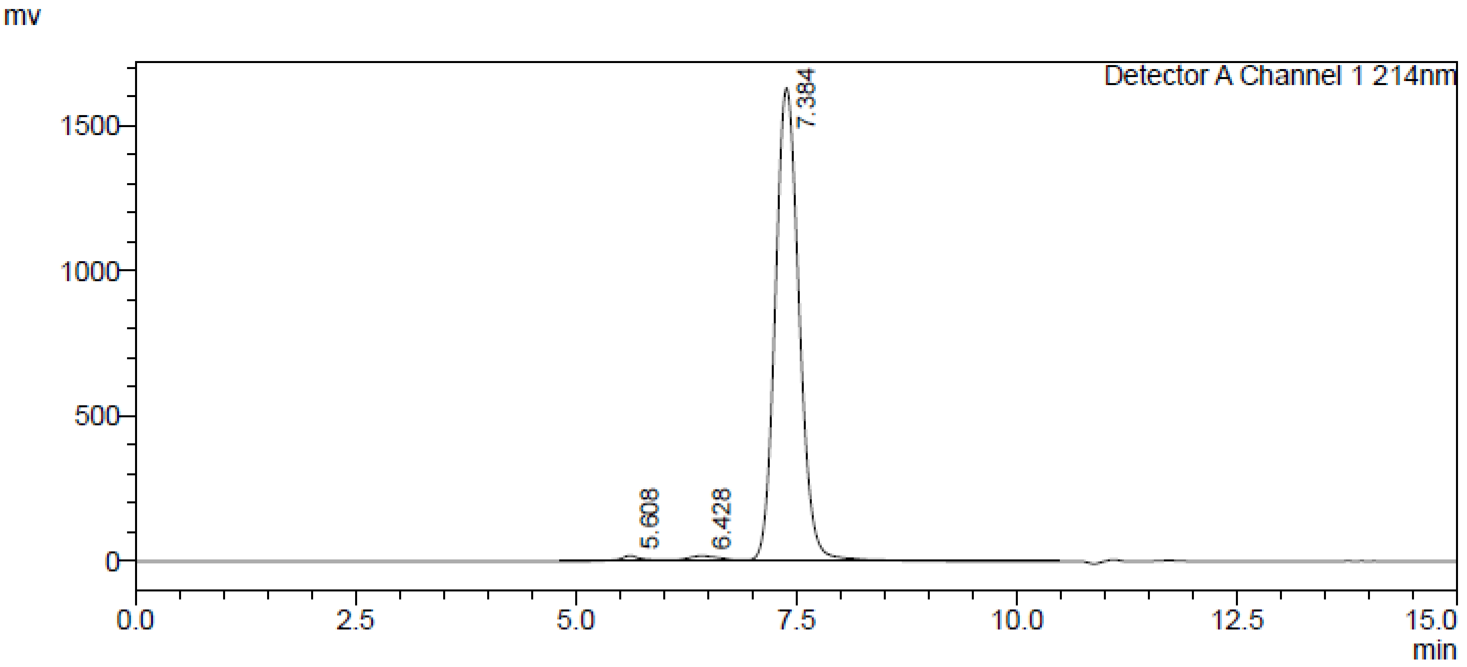

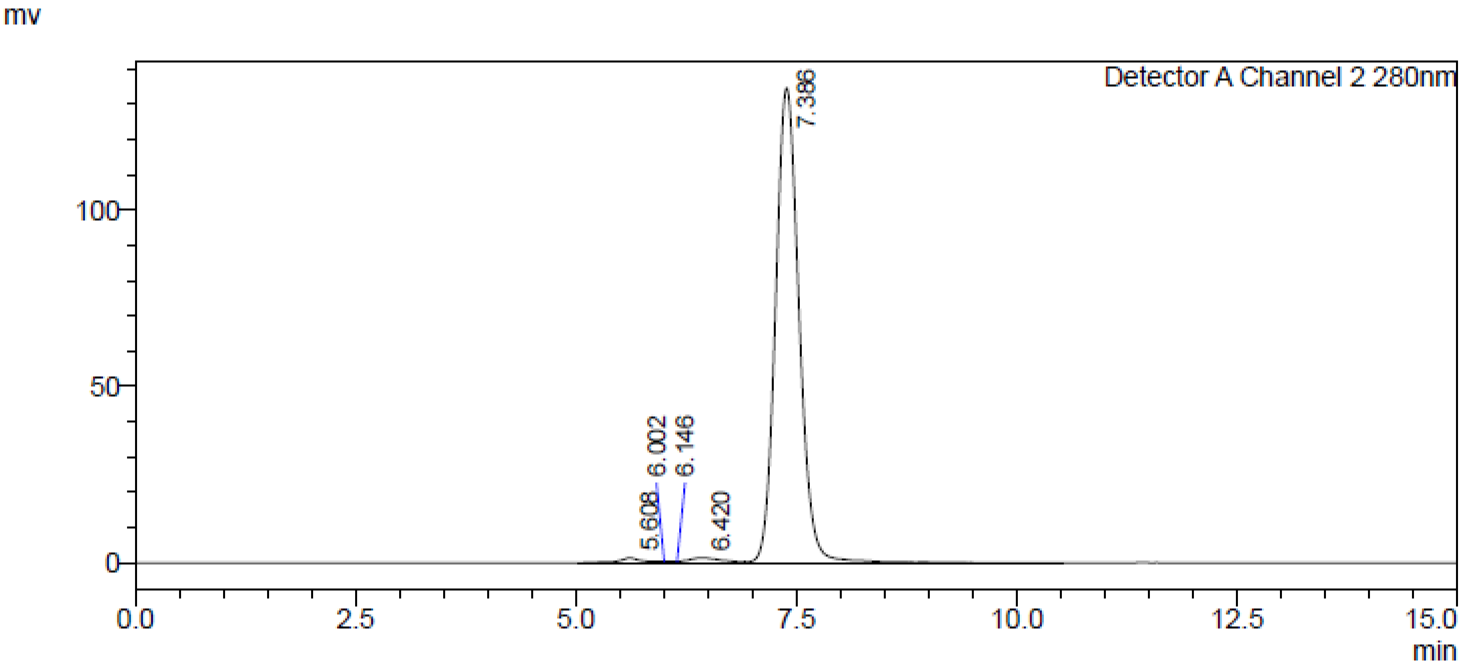

2401*EVO108_2402*EVO30

Figure 5

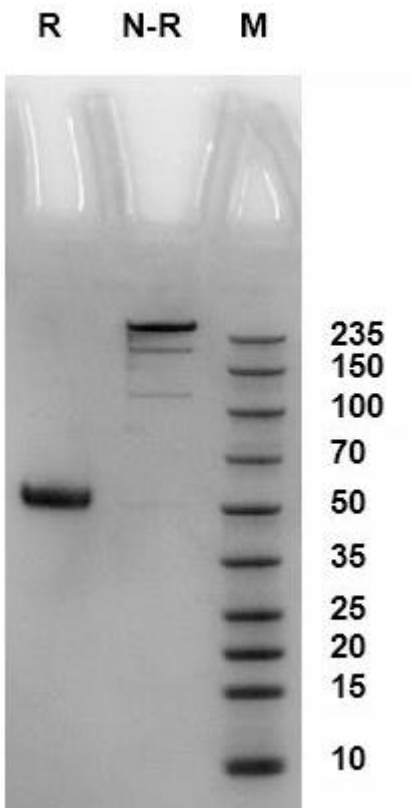

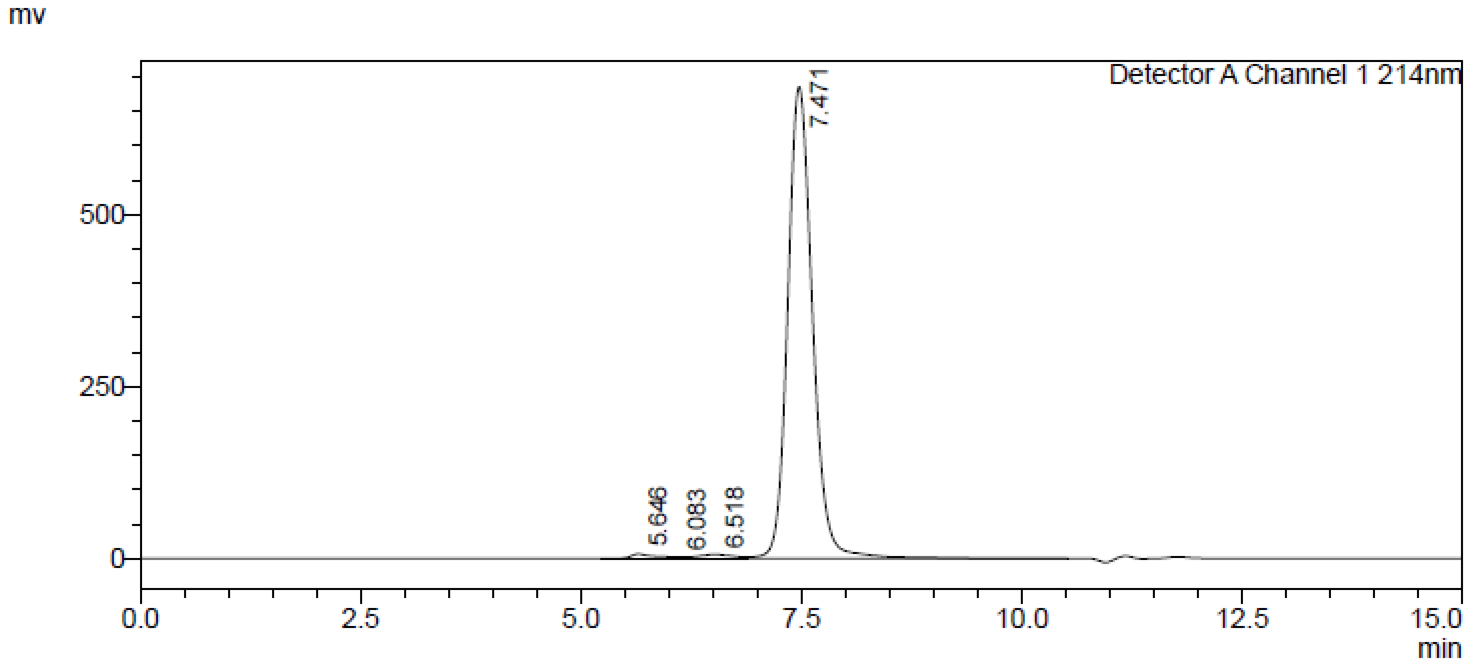

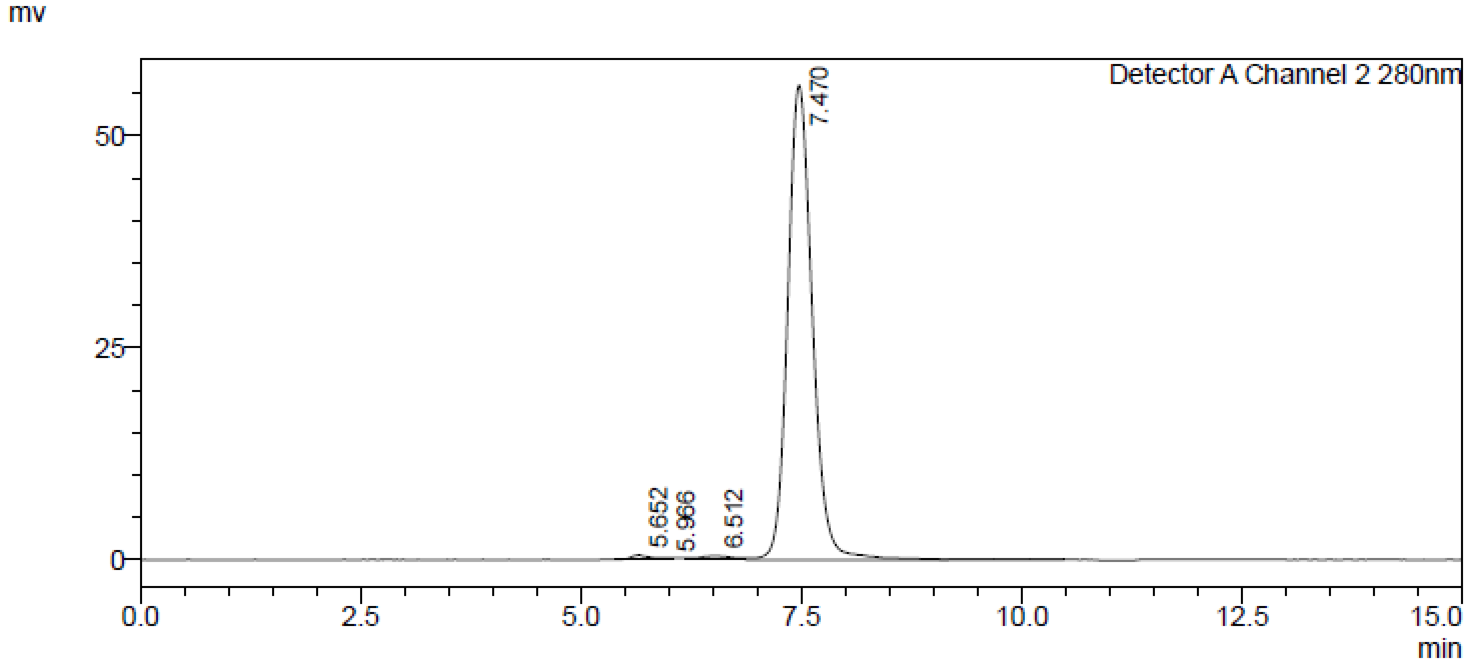

Supplement: Supplementary Figure 5 — List of SDS-Page and SEC results for each antibody production. Includes reference to the main figure where the produced antibody was used. [file DataSheet1.pdf]
